# Supplementary material for: Comprehensive evaluation of deconvolution methods for human brain gene expression
Source: Nat Commun. 2022 Mar 15;13:1358. doi: 10.1038/s41467-022-28655-4 (PMC8924248; doi:10.1038/s41467-022-28655-4)
Supplement: Supplementary file 1 — Supplementary Information [file 41467_2022_28655_MOESM1_ESM.pdf]

SUPPLEMENTARY INFORMATION FOR:

Comprehensive evaluation of deconvolution methods for human brain gene expression

Sutton et al.

Supplementary Note

This supplementary note contains further results on deconvolution of large bulk brain datasets from the GTEx consortium and Parikshak *et al.*

- **Correlation of cell-type composition estimates generated with different methods and signature data**

We found that cell-type composition estimates were highly correlated (*rho* > 0.8) across partial deconvolution methods applied with an appropriate signature, but less so when using the F5 signature, or enrichment algorithms (Supplementary Figures 44-48). For neurons, cell-type proportion estimates showed *rho* > 0.8 for GTEx (except when using the NG or F5 signatures; Supplementary Figure 44), while for the Parikshak et al. data, *rho* was greater than 0.8 in 88.7% of pairwise comparisons of estimates from a partial deconvolution algorithm not using F5 (908/1024). Astrocyte and oligodendrocytes estimates showed similarly high correlations, with the exception of those generated with *xCell* and the F5 signature for astrocytes, further emphasizing the importance of cell-type signature data (Supplementary Figure 45-46). However, composition estimates for lowly-abundant cell-types such as microglia and endothelia showed lower and highly variable correlation coefficients across methods (Supplementary Figure 47-48).

- **Distribution of composition estimates in bulk brain datasets across algorithms, signatures, and brain regions.**

Despite the high correlation of composition estimates for abundant cell-types, when considering the absolute values of estimated cell-type composition, we found that algorithm choice had a substantial impact (Supplementary Figures 49-53). For example, across signatures, *cib* consistently estimated a higher proportion of neurons than either *drs, music,* or *dtangle* (Supplementary Figure 49). We also found that the spinal cord had a substantially higher estimated proportion of oligodendrocytes than other brain regions, holding across algorithms and signatures, consistent with expectations based on the known abundance of oligodendrocytes in the spinal cord. These data suggest that studies where absolute values of composition estimates are required, such as quantitative trait loci for cell-type composition, need careful benchmarking of the choice of deconvolution algorithm.

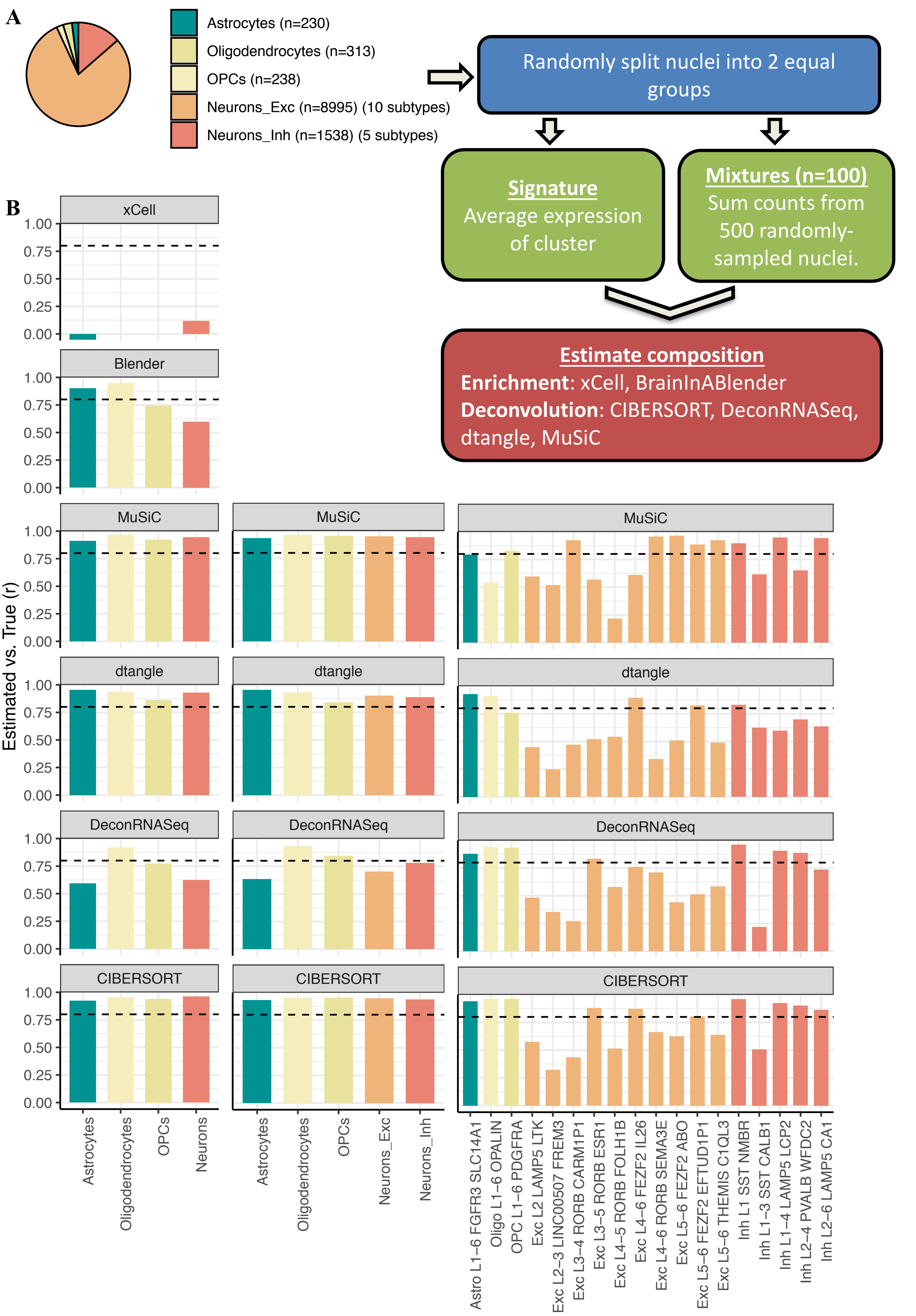

Supplementary Figure 1. Generation and deconvolution of a replication simulated dataset using single-nuclei from the CA dataset. **A.** Process for generating 100 *in silico* mixtures. *Neurons_Exc*: Excitatory Neurons. *Neurons_Inh*: Inhibitory Neurons. *OPCs*: oligodendrocyte precursor cells. **B.** Barplots show Pearson correlation coefficients (r) between true and deconvolution-estimated proportions in 100 *in silico* mixtures. The left column shows results when only major cell-type labels are used in the signature; the middle column shows results when a mix of major cell-type and cell-subtype labels are used in the signature; the right column shows results when only cell-subtype labels are used in the signature. *Dotted line*: *r*=0.8. *DRS*: DeconRNASeq. *CIB*: CIBERSORT. *Blender*: *BrainInABlender*.

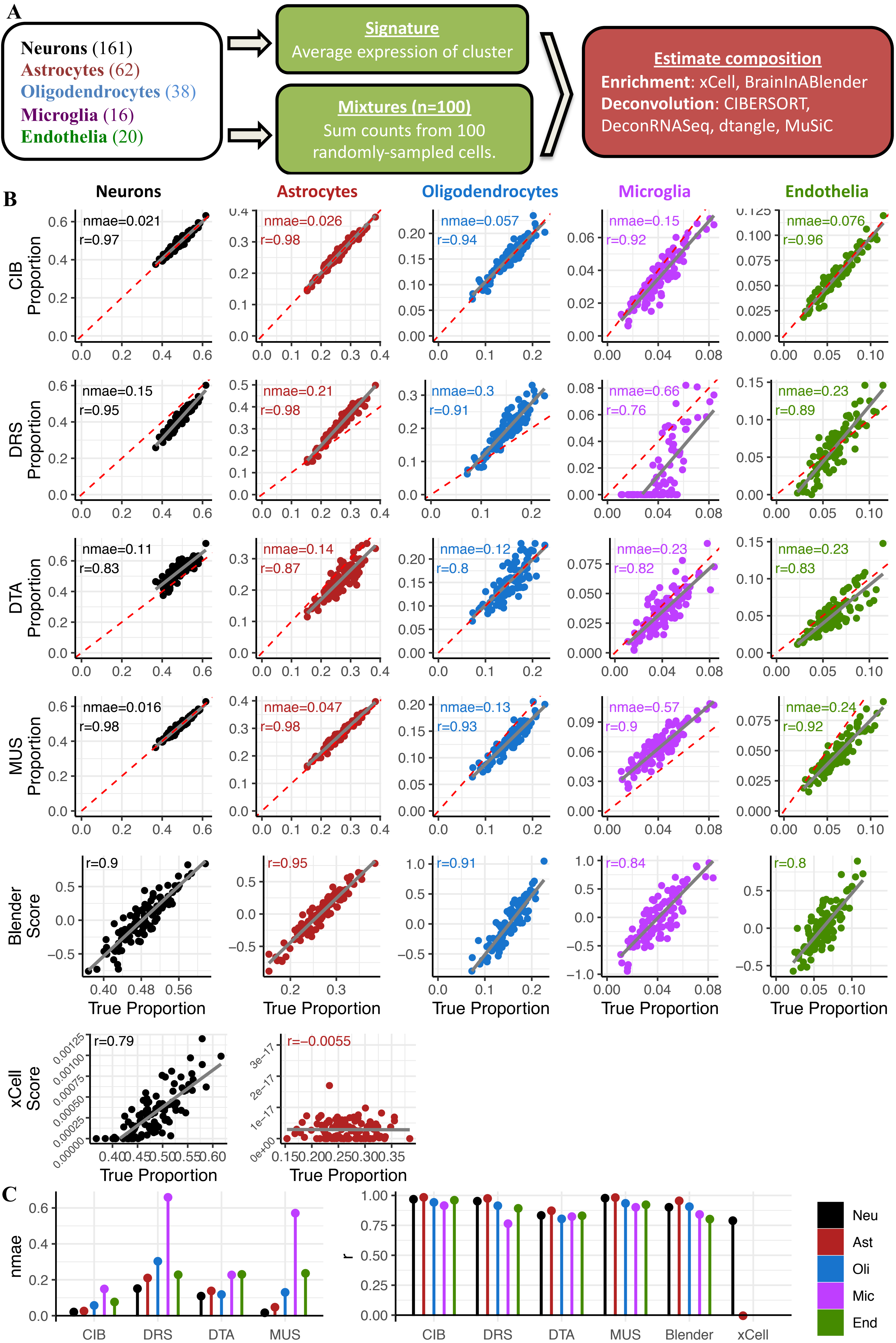

Supplementary Figure 2. Benchmarking deconvolution algorithms on simulated mixtures using data from Darmanis *et al.* **A.** Simulation design. **B.** Scatterplots of estimated proportion (or enrichment score) and true proportion for each cell-type. Columns and rows represent cell-types and algorithms, respectively. *Red dotted line*: y=x. *Grey line*: regression line. *CIB*: CIBERSORT. *DRS*: DeconRNASeq. *DTA*: dtangle. *MUS*: MuSiC. *Blender*: BrainInABlender. *r*: Pearson correlation. *nmae*: normalised mean absolute error. **C.** Barplots of normalised mean absolute error *(nmae*; left) and Pearson correlation coefficients between true and estimated proportions (*r*; right) based on 100 *in silico* mixtures. The x-axis denotes different algorithms.

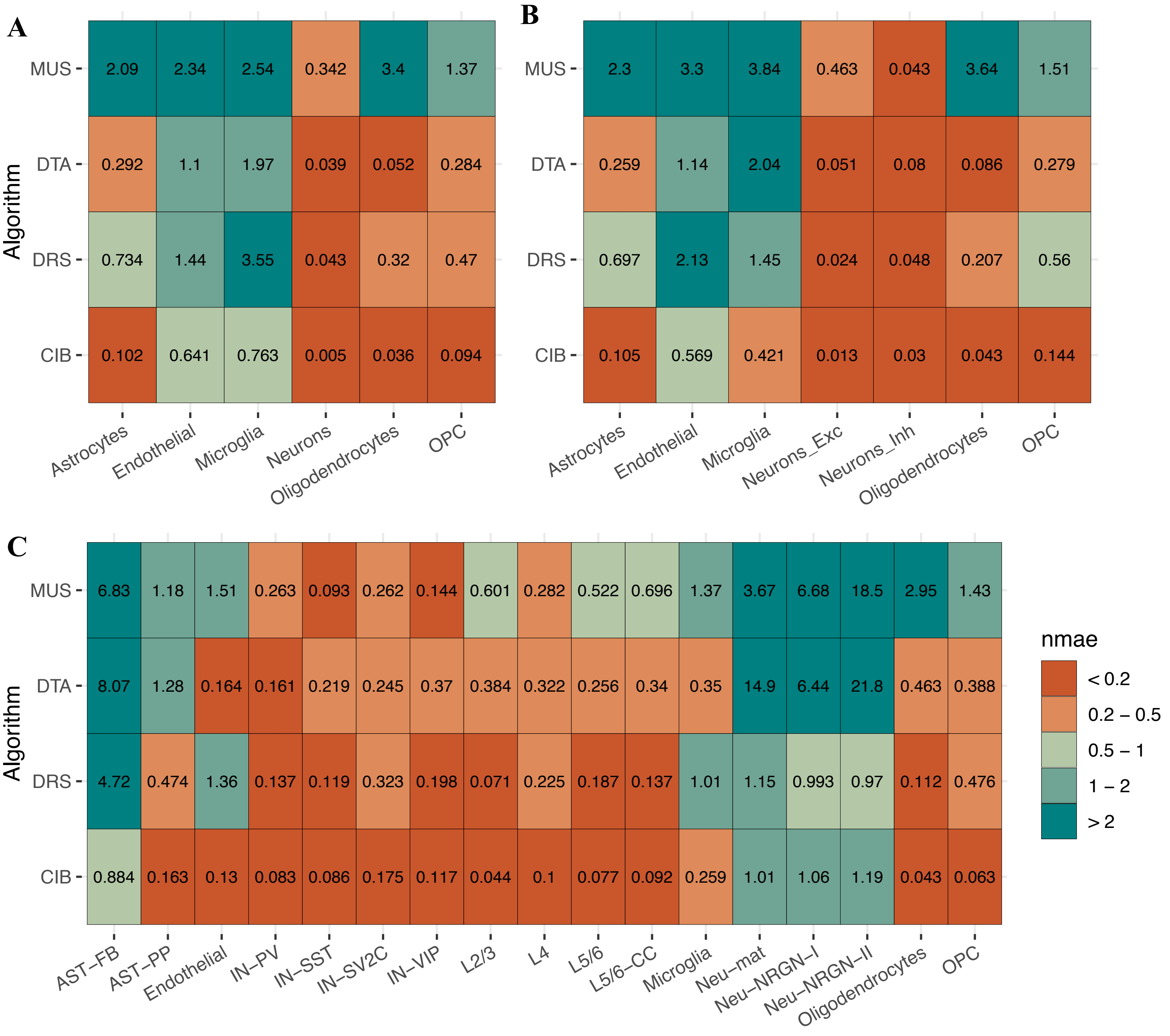

Supplementary Figure 3. Heatmap of normalised mean absolute error (*nmae*) between true and deconvolution-estimated proportions in 100 VL-derived *in silico* mixtures. *Nmae* was calculated as the average error divided by the average true value. Rows and columns represent different algorithms and cell-types, respectively. Entries state the nmae for a given cell-type from a given algorithm. *CIB*: CIBERSORT. *DRS*: DeconRNASeq. *DTA*: dtangle. *MUS*: MuSiC. **A.** Deconvolution using only major cell-type labels in the signature. Legend is per C. **B.** Deconvolution using a mix of major cell-type and cell-subtype labels in the signature. Legend is per C. **C.** Deconvolution using all cell-subtype labels in the signature.

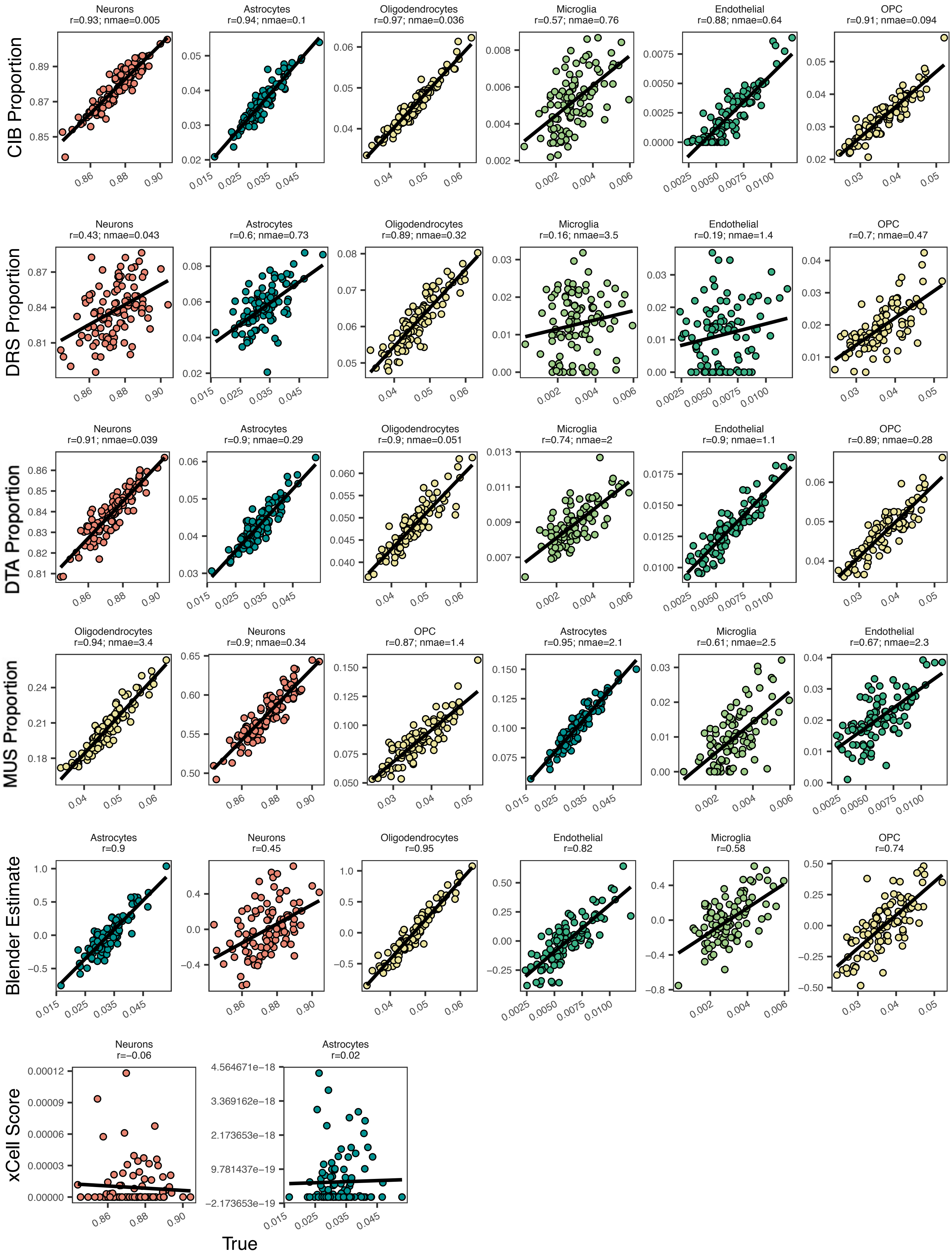

Supplementary Figure 4. Scatterplots of true and deconvolution-estimated proportions in 100 VL-derived *in silico* mixtures. Each row represents a different algorithm. The signature used only major cell-types. *Solid black line*: regression line. *Nmae*: normalised mean absolute error. *r*: Pearson correlation coefficient. Note that nmae was not calculated for xCell and Blender as their output is an enrichment score rather than a proportion. *CIB*: CIBERSORT. *DRS*: DeconRNASeq. *MUS*: MuSiC. *DTA*: dtangle. *Blender:* BrainInABlender.

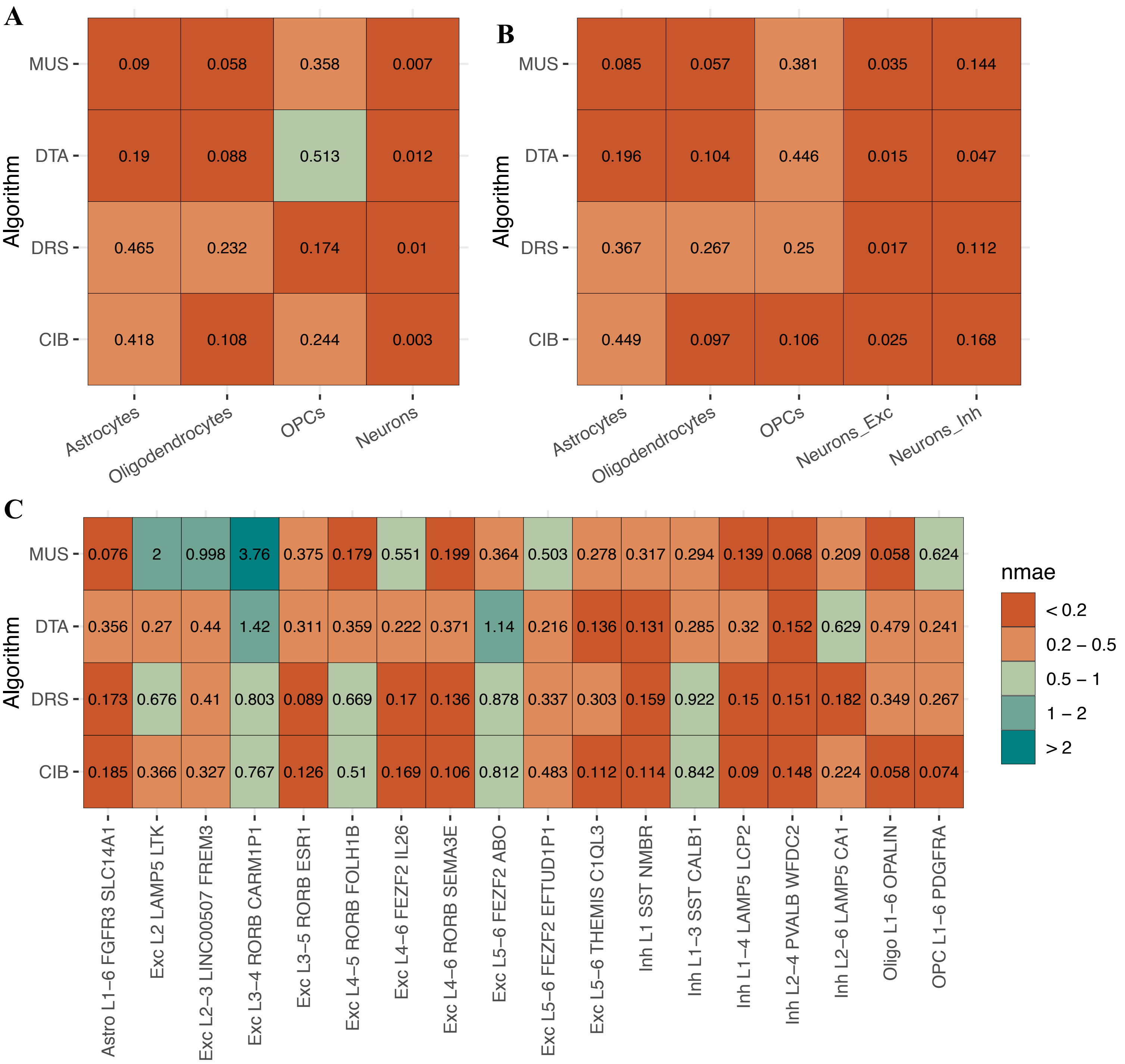

Supplementary Figure 5. Heatmap of normalised mean absolute error (*nmae*) between true and deconvolution-estimated proportions in 100 CA-derived *in silico* mixtures. *Nmae* was calculated as the average error divided by the average true value. Rows and columns represent different algorithms and cell-types, respectively. Entries state the nmae for a given cell-type from a given algorithm. *CIB*: CIBERSORT. *DRS*: DeconRNASeq. *DTA*: dtangle. *MUS*: MuSiC. **A.** Deconvolution using only major cell-type labels in the signature. Legend is per C. **B.** Deconvolution using a mix of major cell-type and cell-subtype labels in the signature. Legend is per C. **C.** Deconvolution using all cell-subtype labels in the signature.

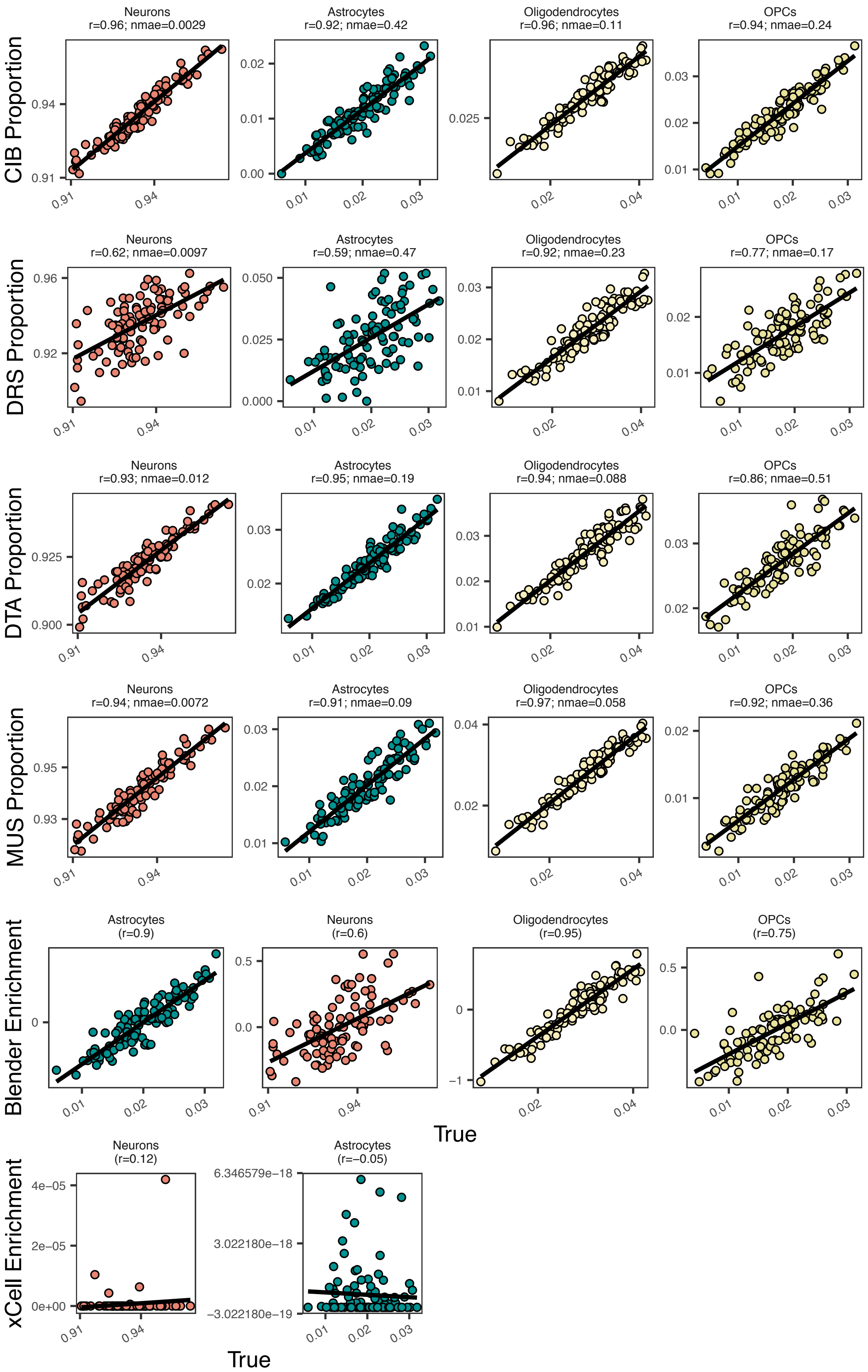

Supplementary Figure 6. Scatterplots of true and deconvolution-estimated proportions in 100 CA-derived *in silico* mixtures. Each row represents a different algorithm. The signature used only major cell-types. *Solid black line*: regression line. *Nmae*: normalised mean absolute error. *r*: Pearson correlation coefficient. Note that nmae was not calculated for xCell and Blender as their output is an enrichment score rather than a proportion. *CIB*: CIBERSORT. *DRS*: DeconRNASeq. *Blender:* BrainInABlender. *DTA*: dtangle. *MUS*: MuSiC.

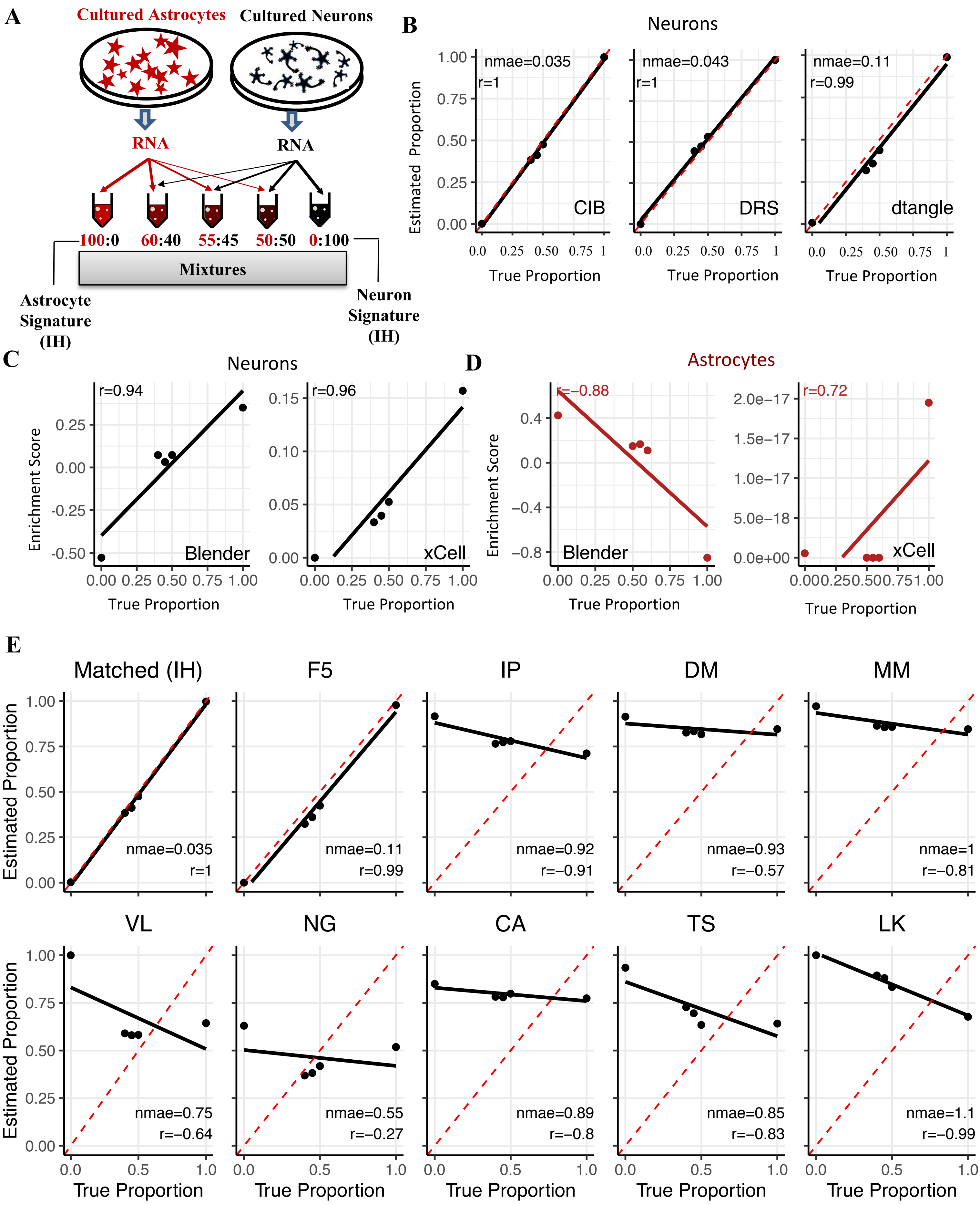

Supplementary Figure 7. Deconvolving mixtures of RNA from cultured neurons and astrocytes. A. Outline of RNA mixtures and its corresponding in-house (IH) signature. **B.** Scatterplots of estimated and true proportions of neurons in the RNA mixtures. Neuronal proportion was estimated using three deconvolution algorithms combined with the matching IH signature. Note that the MuSiC algorithm was not used, as the algorithm is only compatible with single-cell-level signature data. *nmae*: normalised mean absolute error. *r*: Pearson correlation. *CIB*: CIBERSORT. *DRS*: DeconRNASeq. **C.** Scatterplots of true neuronal proportion versus neuron enrichment scores obtained with BrainInABlender (left) and xCell (right) in the RNA mixtures. **D.** Scatterplots of true astrocyte proportion versus astrocyte enrichment scores obtained with BrainInABlender (left) and xCell (right) in the RNA mixtures. **E.** Scatterplots of true versus estimated neuronal proportion in the RNA mixture as a function of signature choice. The title of each plot denotes which signature was used in deconvolution; for further details about signatures, see methods. Deconvolution was performed using CIBERSORT. All signatures were filtered to include only neuronal and astrocyte expression values. Note that the top-left panel ("Matched (IH)") is the same as in the left panel of B.

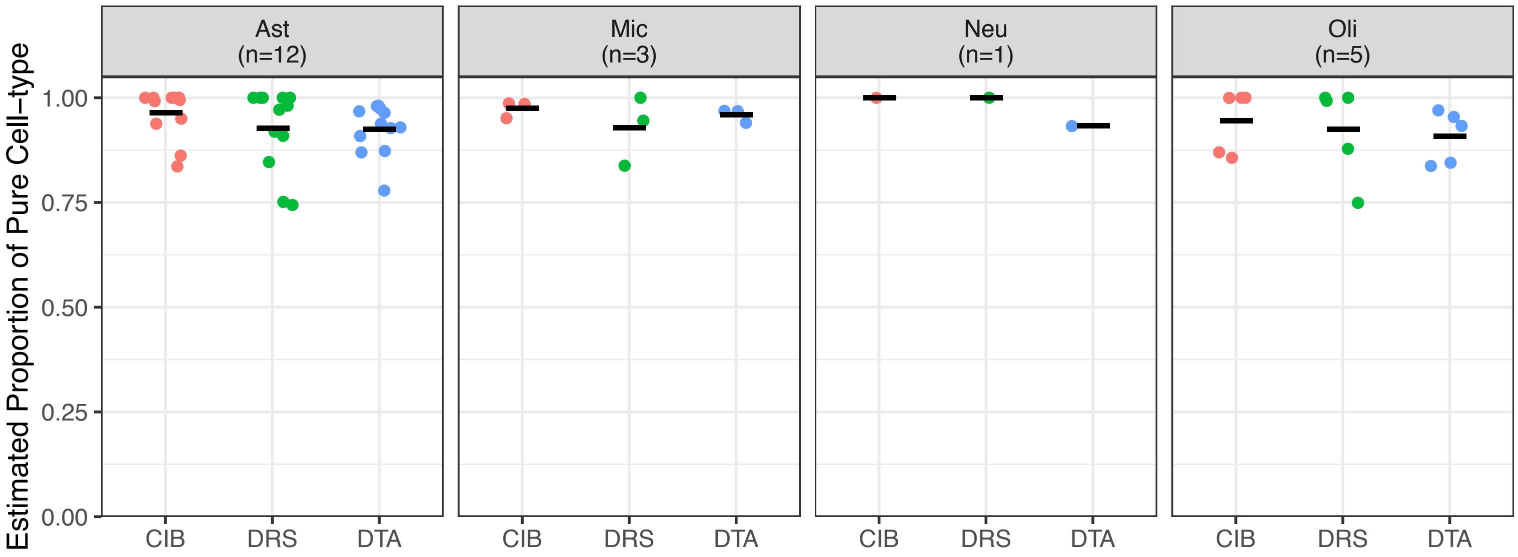

Supplementary Figure 8. Estimated proportion in immuno-panned purified brain cell-types. Data from Zhang *et al.* (2016) were deconvolved using three algorithms (x-axis) combined with the IP signature (which is derived from the Zhang data). *Thick horizontal line*: mean. *Neu*: neurons. *Ast*: astrocytes. *Oli*: oligodendrocytes. *Mic*: microglia. *CIB*: CIBERSORT. *DRS*: DeconRNASeq. *DTA*: dtangle. Note that MuSiC was not applied as it requires single-cell or –nucleus data for its signature.

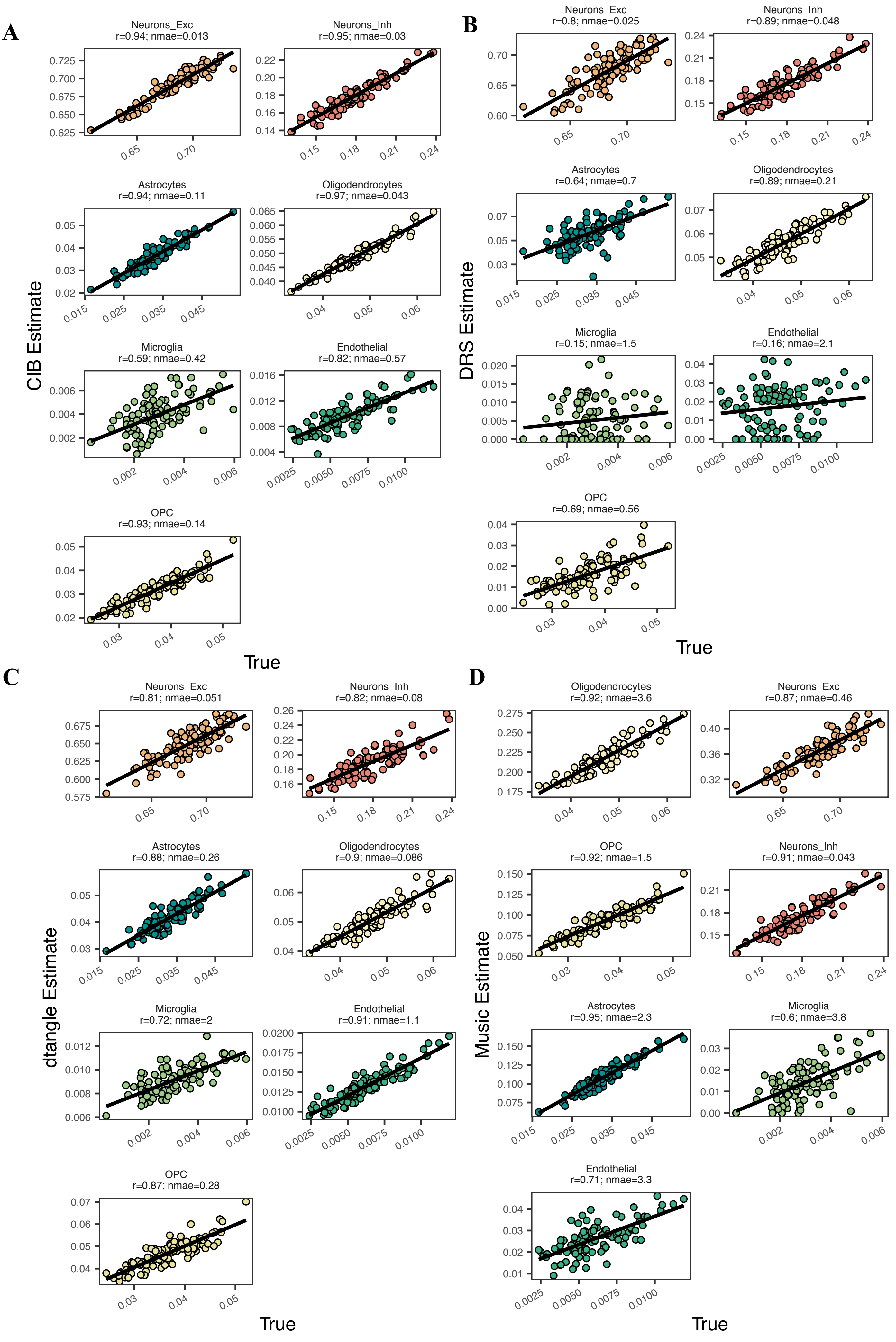

Supplementary Figure 9. Scatterplots of true and deconvolution-estimated proportions in 100 VL-derived *in silico* mixtures. The signature used a range of cell-subtypes and major cell-types. **A.** CIBERSORT deconvolution. **B.** DeconRNASeq. **C.** dtangle. **D.** MuSiC. *Solid black line*: regression line. *Nmae*: normalised mean absolute error. *r*: Pearson correlation coefficient. *Neurons_Inh and Neurons_Exc*: Inhibitory and excitatory neurons, respectively. *CIB*: CIBERSORT. *DRS*: DeconRNASeq.

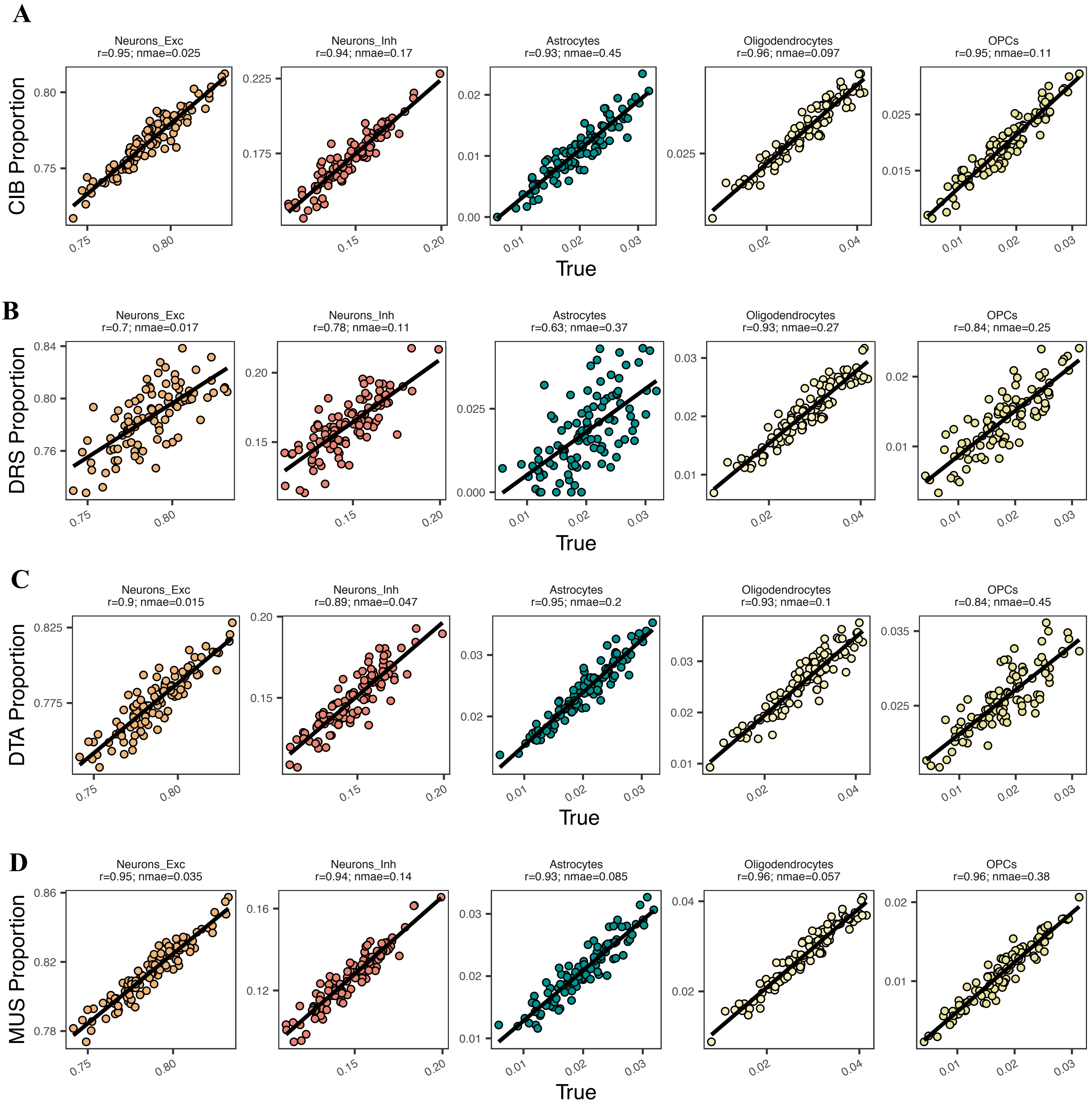

Supplementary Figure 10. Scatterplots of true and deconvolution-estimated proportions in 100 CA-derived *in silico* mixtures. Each row represents a different algorithm. The signature used a range of cell-subtypes and major cell-types. **A.** CIBERSORT deconvolution. **B.** DeconRNASeq. **C.** dtangle. **D.** MuSiC. *Solid black line*: regression line. *Nmae*: normalised mean absolute error. *r*: Pearson correlation coefficient. *Neurons_Inh and Neurons_Exc*: Inhibitory and excitatory neurons, respectively. *CIB*: CIBERSORT. *DRS*: DeconRNASeq. *DTA*: dtangle. *MUS*: MuSiC.

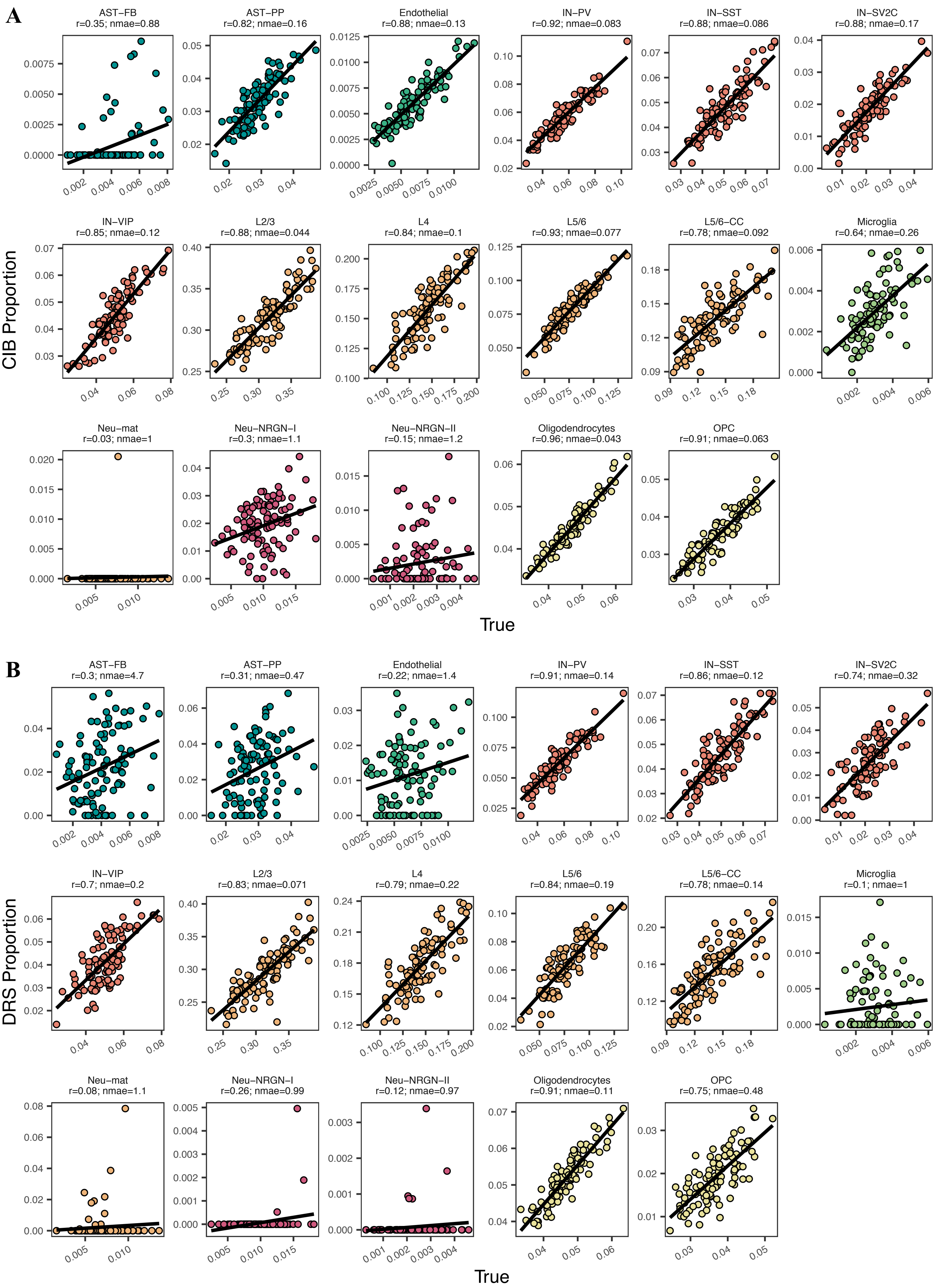

Supplementary Figure 11. Scatterplots of true and deconvolution-estimated proportions in 100 VL-based *in silico* mixtures. The signature used all cell-subtypes from the original publication by Velmeshev *et al.* (2019). **A.** CIBERSORT deconvolution. **B.** DeconRNASeq. *Solid black line*: regression line. *Nmae*: normalised mean absolute error. *r*: Pearson correlation coefficient. *CIB*: CIBERSORT. *DRS*: DeconRNASeq.

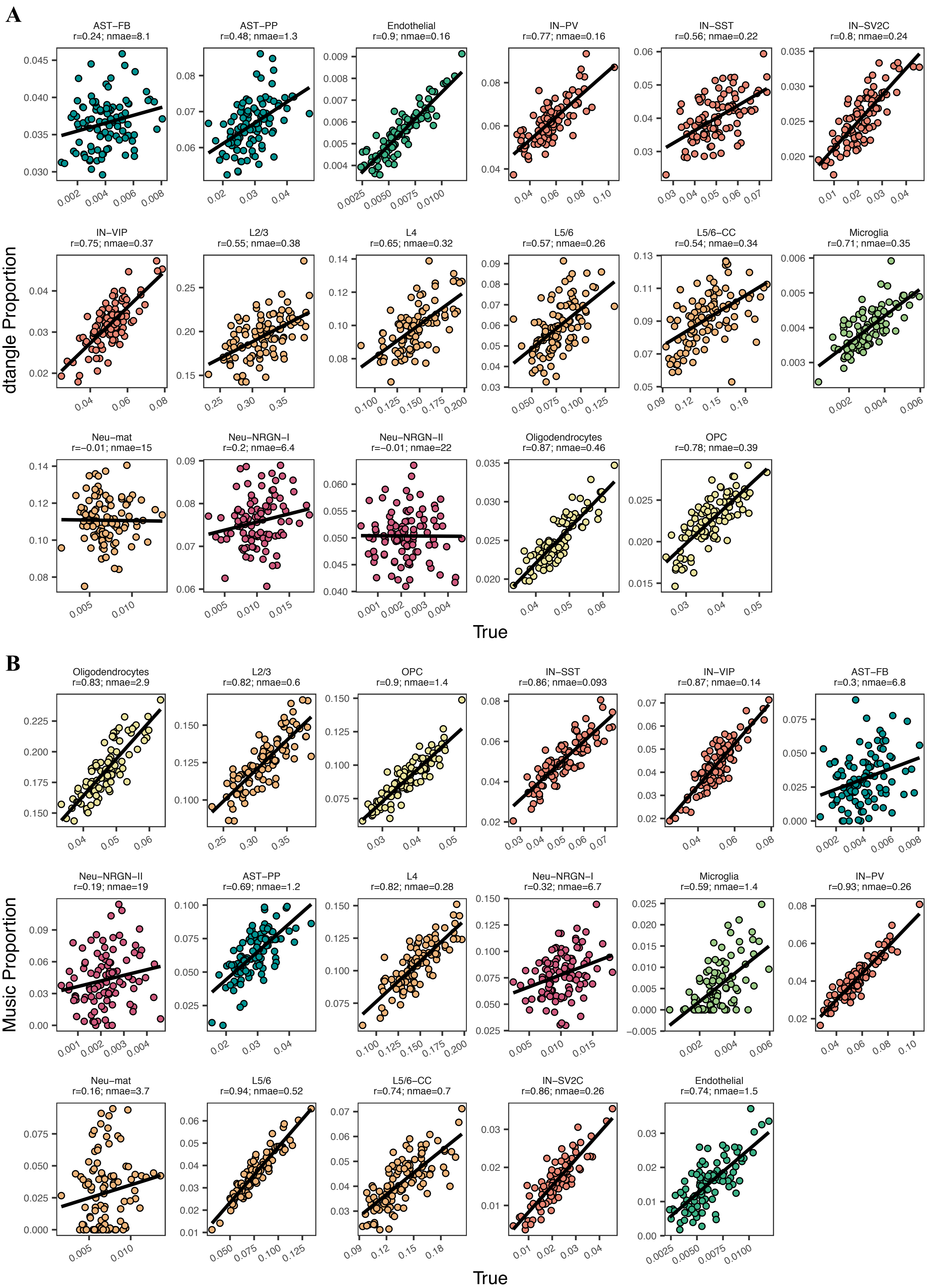

Supplementary Figure 12. Scatterplots of true and deconvolution-estimated proportions in 100 VL-based *in silico* mixtures. The signature used all cell-subtypes from the original publication by Velmeshev *et al.* (2019). **A.** dtangle deconvolution. **B.** MuSiC. *Solid black line*: regression line. *Nmae*: normalised mean absolute error. *r*: Pearson correlation coefficient.

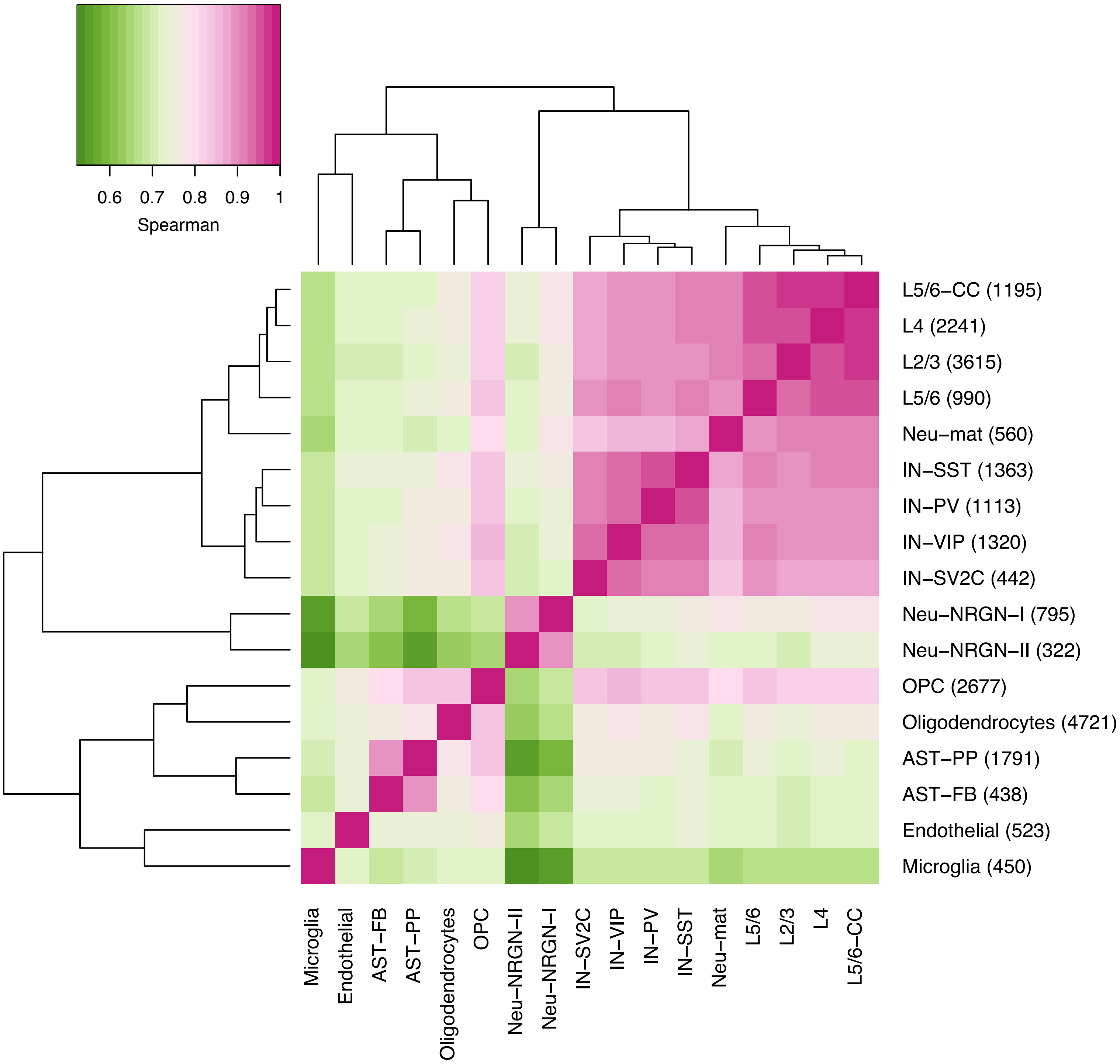

Supplementary Figure 13. Heatmap of Spearman correlations between cell-subtypes in the VL dataset. Correlations were calculated using all expressed genes. Labels are taken from the original publication**.** Numbers in brackets on the right axis labels indicate the number of nuclei in that class.

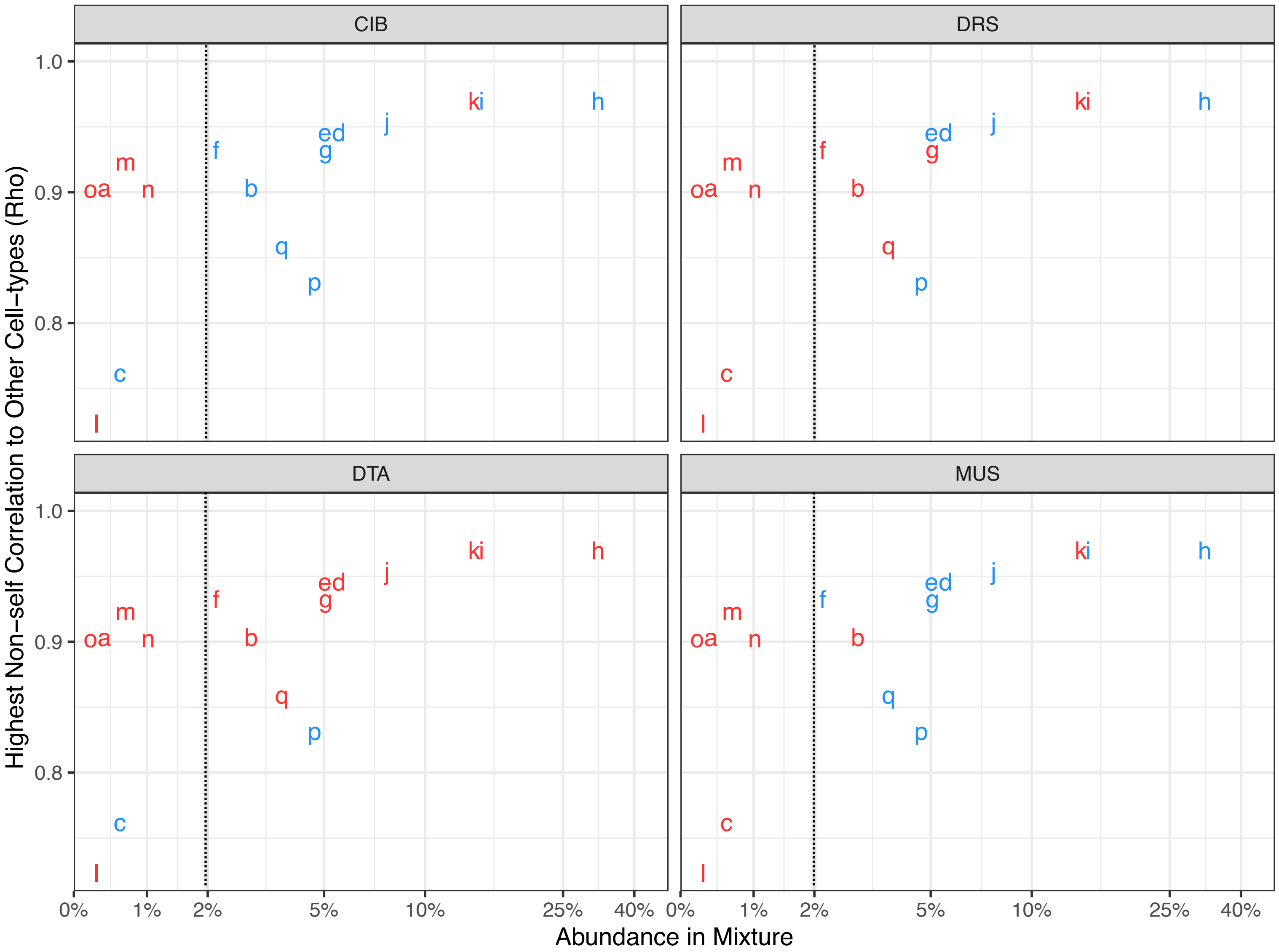

Label	Celltype
a	AST-FB
b	AST-PP
c	Endothelial
d	IN-PV
e	IN-SST
f	IN-SV2C
g	IN-VIP
h	L2/3
i	L4
j	L5/6
k	L5/6-CC
l	Microglia
m	Neu-mat
n	Neu-NRGN-I
o	Neu-NRGN-II
p	Oligodendrocytes
q	OPC

Deconvolution accuracy

- Poor ($r < 0.8$)
- Good ($r > 0.8$)

Supplementary Figure 14. Effect of cell-type abundance and collinearity on deconvolution accuracy in VL-based simulations. Each point represents a cell-subtype in the Velmeshev dataset. Points are labelled by text indicating the cell-subtype classification. Colours represent a binary code for good and poor deconvolution performance based on the Pearson correlation (r) between estimated and true proportion using the titular algorithm with the VL signature. *Rho*: Spearman correlation coefficient. *X axis*: mean abundance across the 100 simulated mixtures. *Y axis*: the highest correlation a cell-subtype has to any of the other cell-subtypes in the dataset, indicating collinearity. Note that “o” and “a” are partially overlapping at x=0.5, y = 0.9, as are “k” and “I” at x=15 and y = 0.96. *CIB*: CIBERSORT. *DRS*: DeconRNASeq. *DTA*: dtangle. *MUS*: MuSiC.

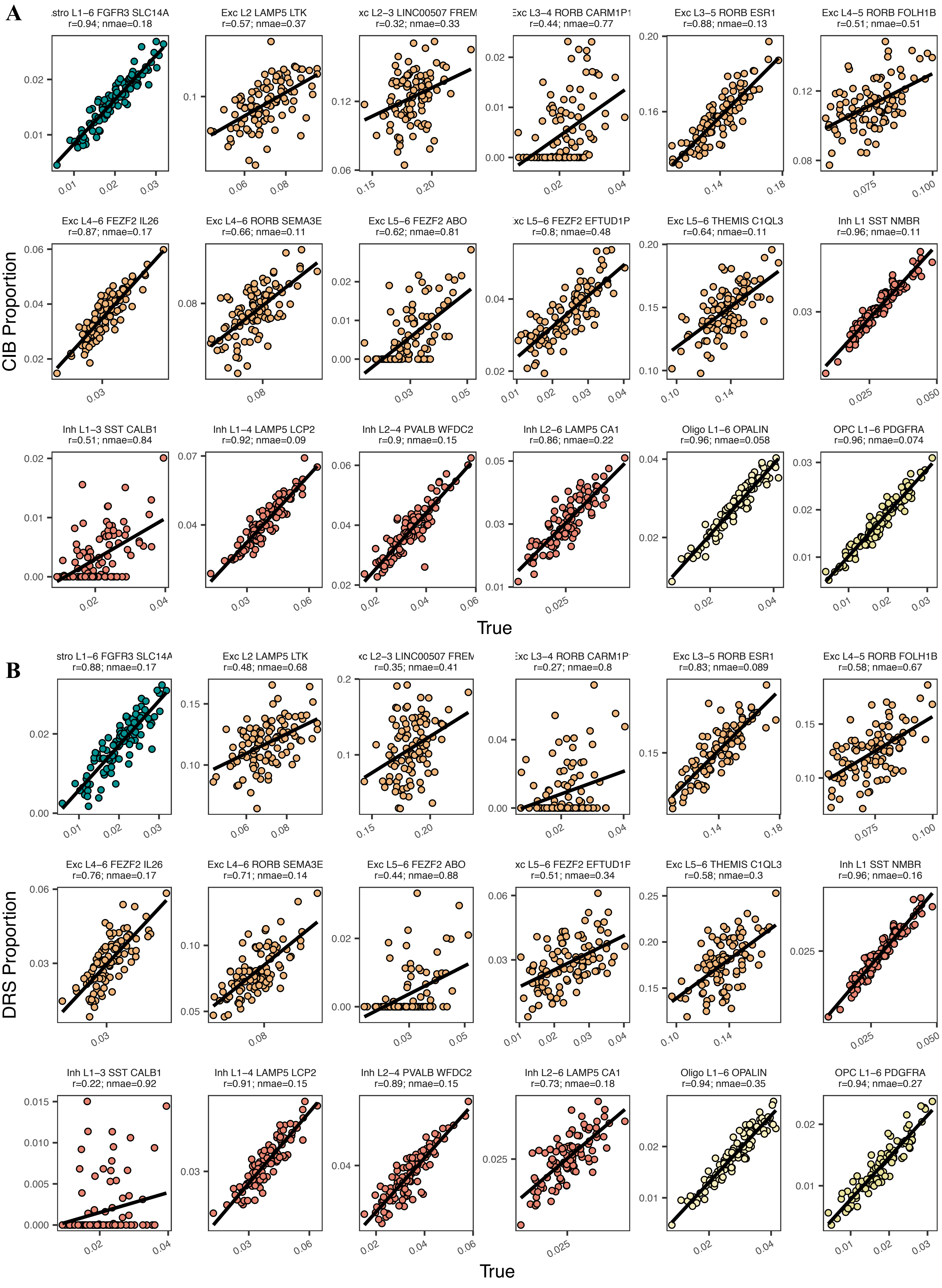

Supplementary Figure 15. Scatterplots of true and deconvolution-estimated proportions in 100 CA-based *in silico* mixtures. The signature used all cell-subtypes from the original publication by Hodge *et al.* (2019). **A.** CIBERSORT deconvolution. **B.** DeconRNASeq. *Solid black line*: regression line. *Nmae*: normalised mean absolute error. *r*: Pearson correlation coefficient. *CIB*: CIBERSORT. *DRS*: DeconRNASeq.

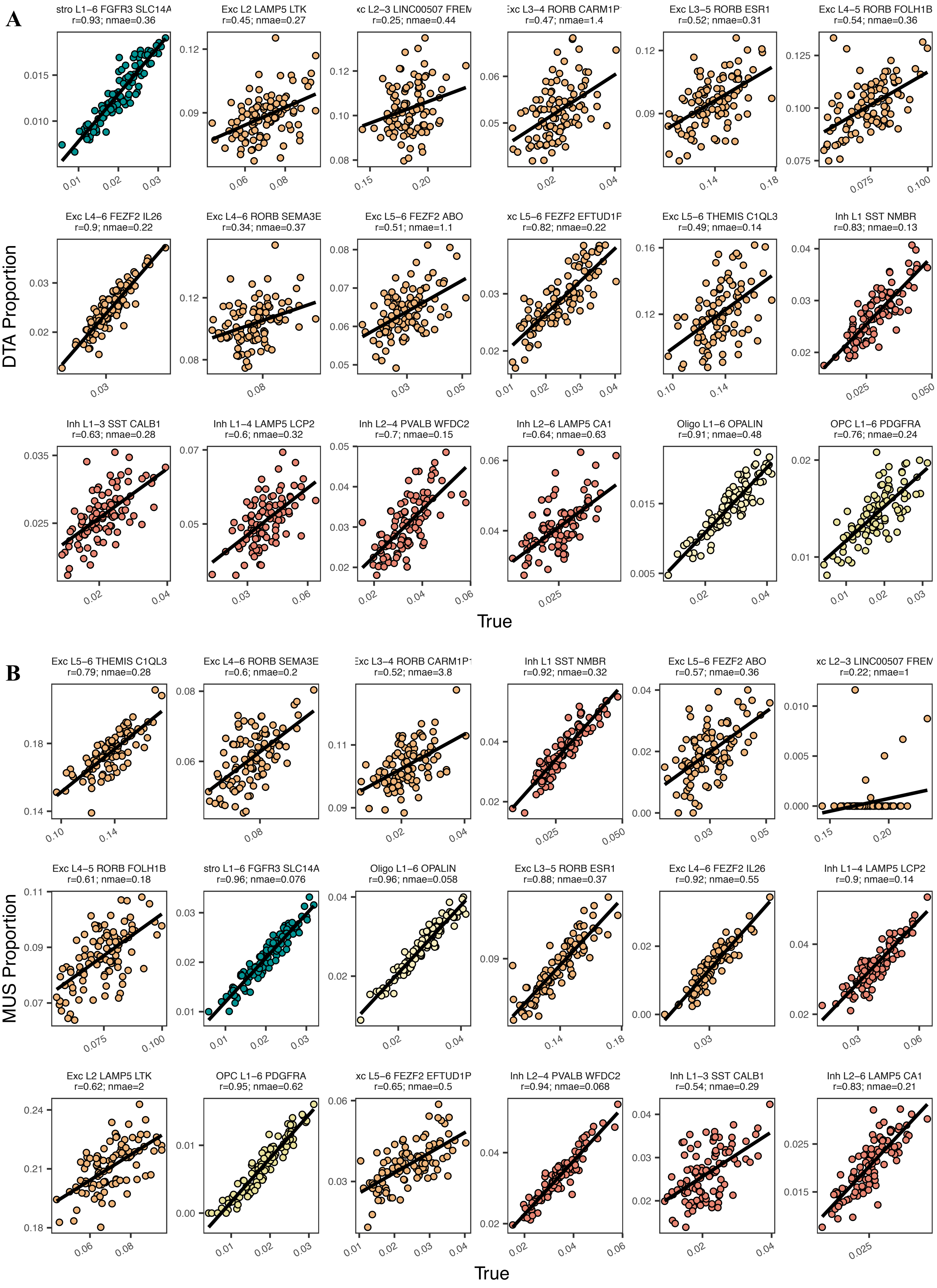

Supplementary Figure 16. Scatterplots of true and deconvolution-estimated proportions in 100 CA *in silico* mixtures. The signature used all cell-subtypes from the original publication by Hodge *et al.* (2019). **A.** dtangle deconvolution. **B.** MuSiC deconvolution. *Solid black line*: regression line. *Nmae*: normalised mean absolute error. *r*: Pearson correlation coefficient. *DTA*: dtangle. *MUS*: MuSiC.

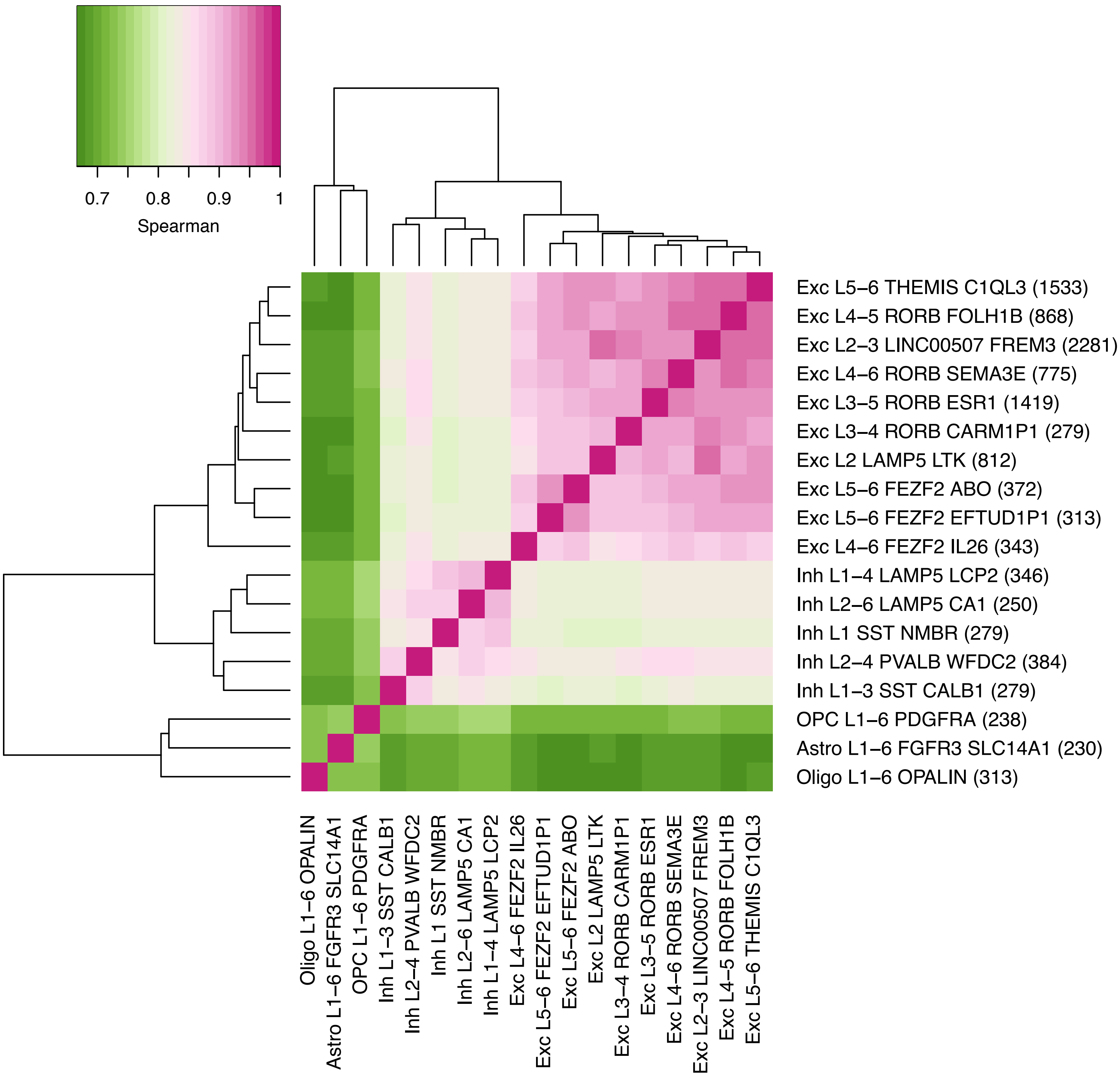

Supplementary Figure 17. Heatmap of Spearman correlations between cell-subtypes in the CA dataset. Correlations were calculated using all expressed genes. Labels are taken from the original publication. Numbers in brackets on the right axis labels indicate the number of nuclei in that class.

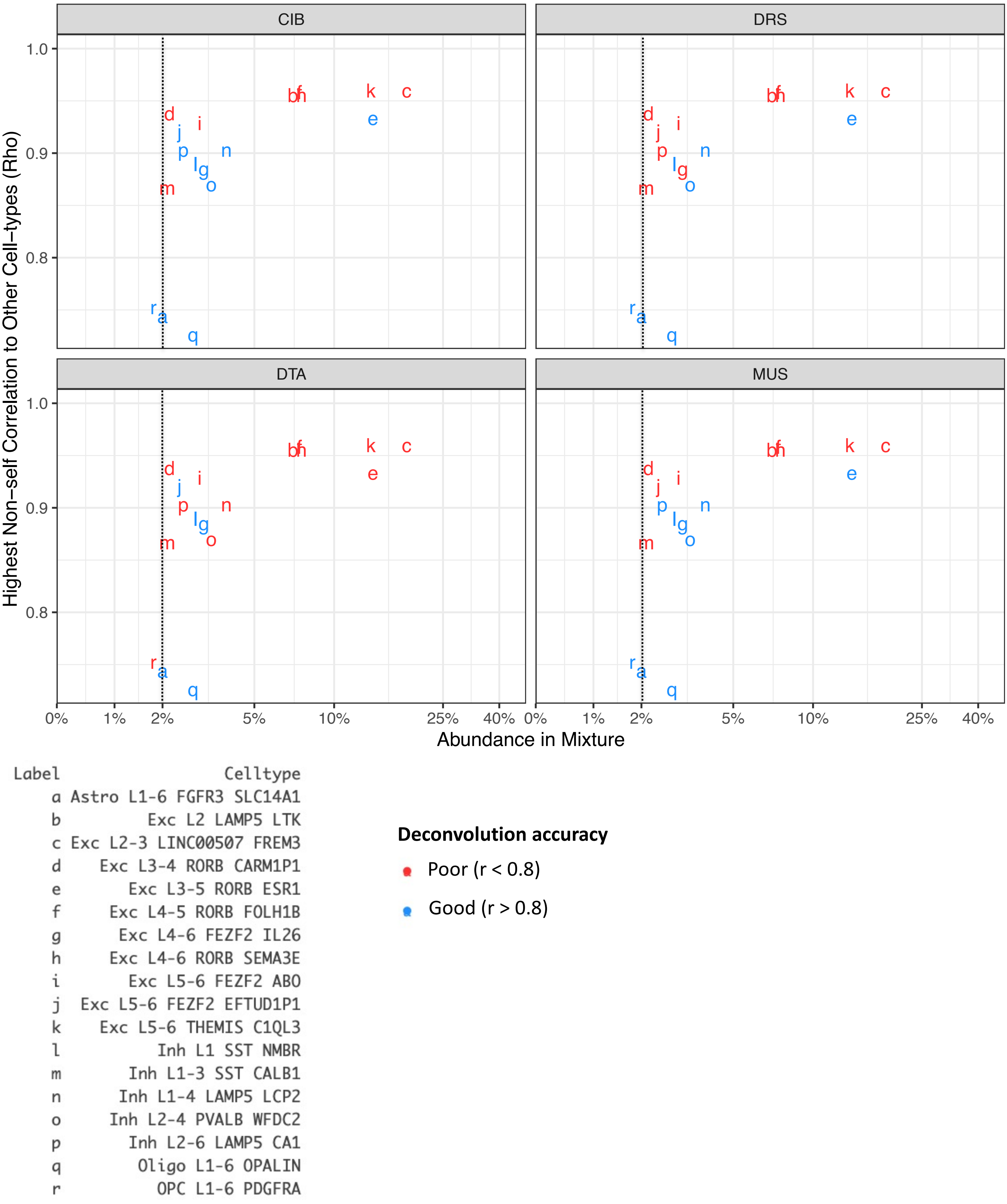

Supplementary Figure 18. Effect of cell-type abundance and collinearity on deconvolution accuracy in CA-based *in silico* simulations. Each point represents a cell-subtype in the CA dataset. Points are labelled by text indicating the cell-subtype classification. Colours represent a binary code for good and poor deconvolution performance based on the Pearson correlation (r) between estimated and true proportion using the titular algorithm with the CA signature. *Rho*: Spearman correlation coefficient. *X axis*: mean abundance across the 100 simulated mixtures. *Y axis*: the highest correlation a cell-subtype has to any of the other cell-subtypes in the dataset, indicating collinearity. Note that the following labels are partially overlapping: "l", "g", and "o" at x=3, y=0.87; and "b", "f", and "h" at x=7, y=0.95. *CIB*: CIBERSORT. *DRS*: DeconRNASeq. *DTA*: dtangle. *MUS*: MuSiC.

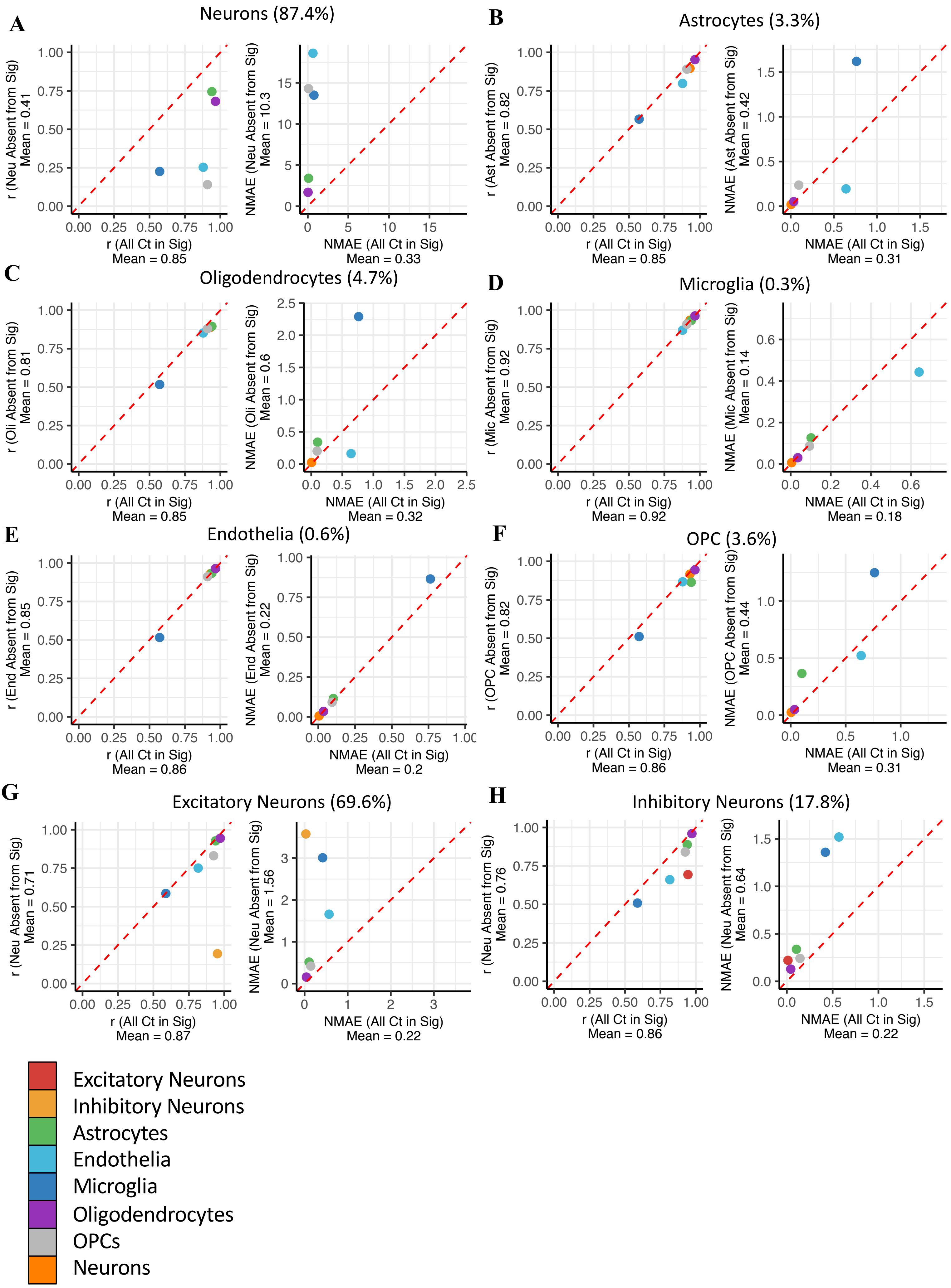

Supplementary Figure 19. Effect of removing cell-types or cell-subtypes from the signature matrix. For each cell-type, its mean abundance in the mixtures is shown in backets, and scatterplots display the deconvolution accuracy when all cell-types are present in the signature (x-axis) *vs.* when the cell-type is absent from signature (y-axis). Accuracy is measured as either Pearson correlation coefficient (*r*; left panel) or normalised mean absolute error (NMAE; right panel). Calculations of mean *r* and mean NMAE for the x-axis label do not include the absent cell-type, and thus differ across plots. *Dotted red line:* y = x. **A.** Removing neurons. **B.** Removing astrocytes. **C.** Removing oligodendrocytes. **D.** Removing microglia. **E.** Removing endothelia. **F.** Removing OPCs. **G.** Removing excitatory neurons. **H.** Removing inhibitory neurons.

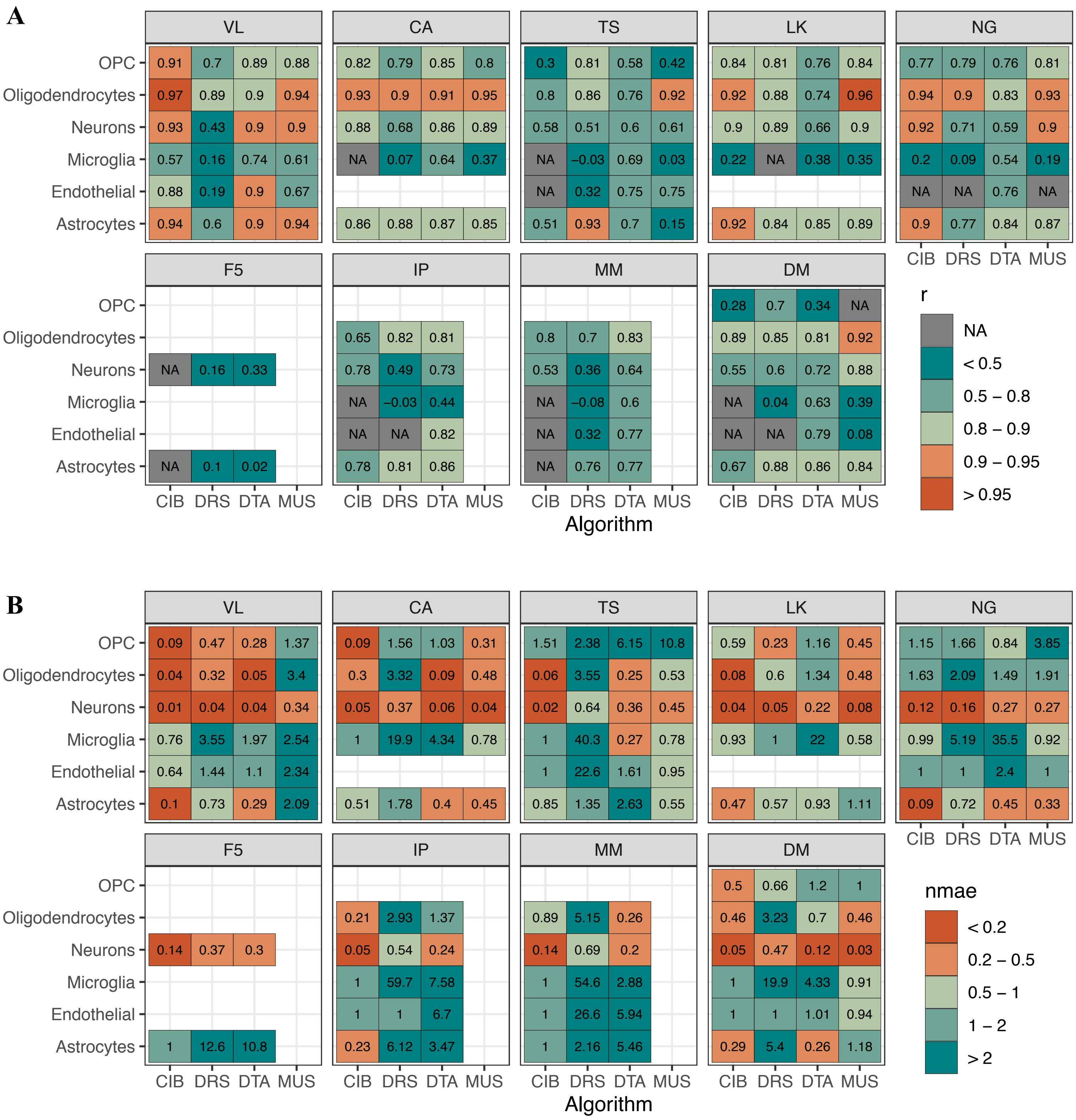

Supplementary Figure 20. Effect of varying the signature in VL-based simulated mixtures. Heatmaps of Pearson correlation (*r*; **A.**) and normalised mean absolute error (nmae; **B.**) for estimated versus true proportion when varying the reference signature (grouped by grey boxes). Columns represent different algorithms with which the signature was combined, while rows represent different cell-types. Thus, each entry represents the statistic for a cell-type when deconvolution is performed with a given signature/algorithm combination. The mixtures are 100 *in silico* VL simulations. Signatures only included a pan-neuronal expression profile, rather than excitatory or inhibitory sub-types. Blank squares indicate that the cell-type was not present in the signature, and thus no statistic was calculated. Grey squares indicate NA, indicating that the cell-type was present in the signature but the statistic could not be calculated; for *r*, this means there was no variance in the composition estimates, typically meaning all 100 samples' estimates were 0 or 1. For more details about signature characteristics, see methods. *CIB*: CIBERSORT. *DRS*: DeconRNASeq. *DTA*: dtangle. *MUS*: MuSiC.

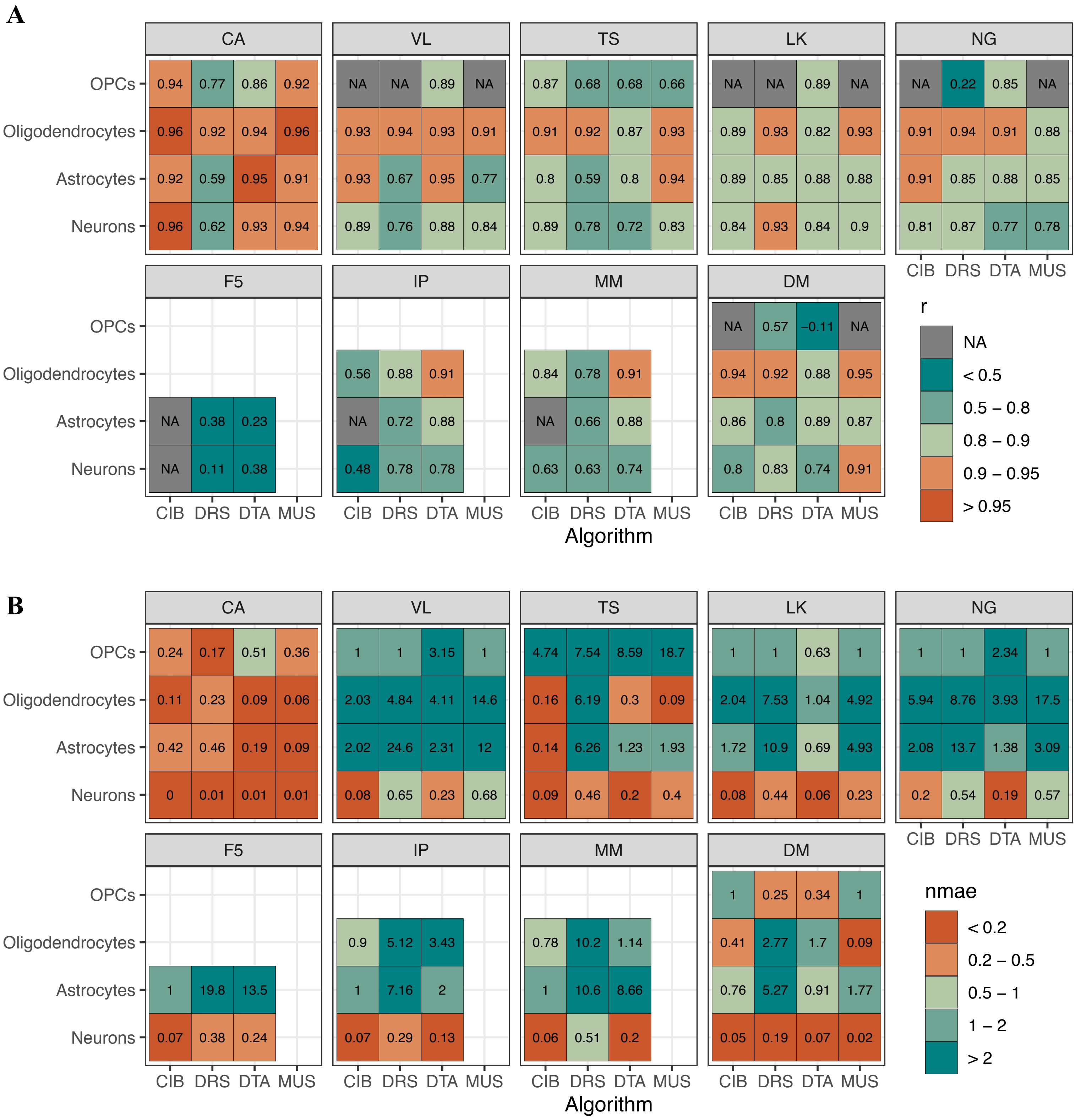

Supplementary Figure 21. Effect of varying the signature in CA-based simulated mixtures. Heatmaps of Pearson correlation (*r*; **A.**) and normalised mean absolute error (nmae; **B.**) for estimated versus true proportion when varying the reference signature (grouped by grey boxes). Columns represent different algorithms with which the signature was combined, while rows represent different cell-types. Thus, each entry represents the statistic for a cell-type when deconvolution is performed with a given signature/algorithm combination The mixtures are 100 *in silico* CA simulations. Signatures only included a pan-neuronal expression profile, rather than excitatory or inhibitory sub-types. Blank squares indicate that the cell-type was not present in the signature, and thus no statistic was calculated. Grey squares indicate NA, indicating that the cell-type was present in the signature but the statistic could not be calculated; for *r*, this means there was no variance in the composition estimates, typically meaning all 100 samples' estimates were 0 or 1. For more details about signature characteristics (grouped by boxes), see methods. *CIB*: CIBERSORT. *DRS*: DeconRNASeq. *DTA*: dtangle. *MUS*: MuSiC.

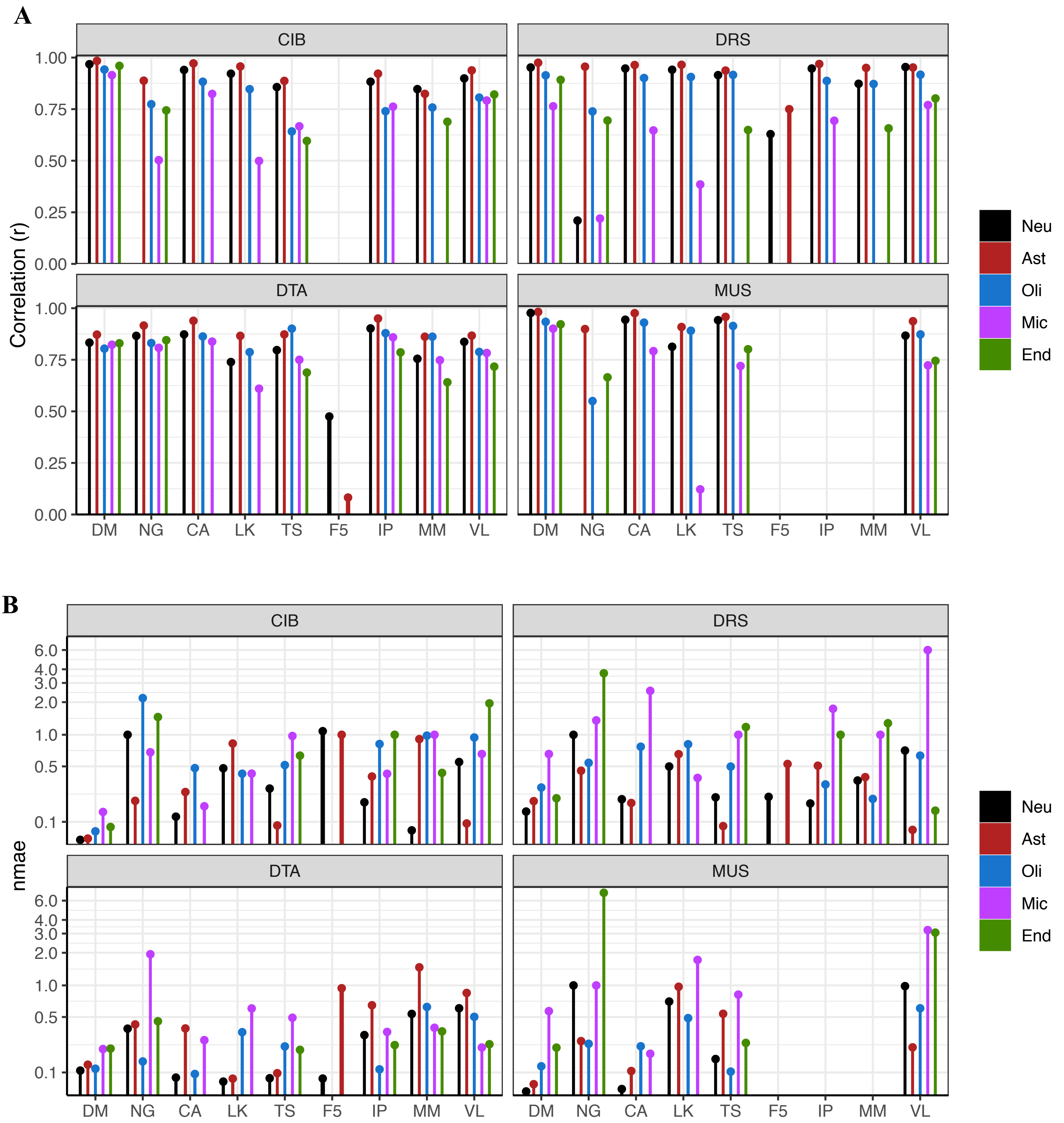

Supplementary Figure 22. Effect of varying the signature in DM-based simulated mixtures. Barplots of Pearson correlation (*r;* **A.**) and normalised mean absolute error (nmae; **B.**) for estimated versus true proportion when varying the reference signature. Panels represent algorithms, with different signatures on the x-axis. Each coloured bar represents the statistic for a cell-type when using a given signature/algorithm combination. Signatures only included a pan-neuronal expression profile, rather than excitatory or inhibitory sub-types. *Neu*: Neurons. *Ast*: Astrocytes. *Oli*: Oligodendrocytes. *Mic*: Microglia. *End*: Endothelia. *CIB*: CIBERSORT. *DRS*: DeconRNASeq. *DTA*: dtangle. *MUS*: MuSiC. For more details about signature characteristics, see methods.

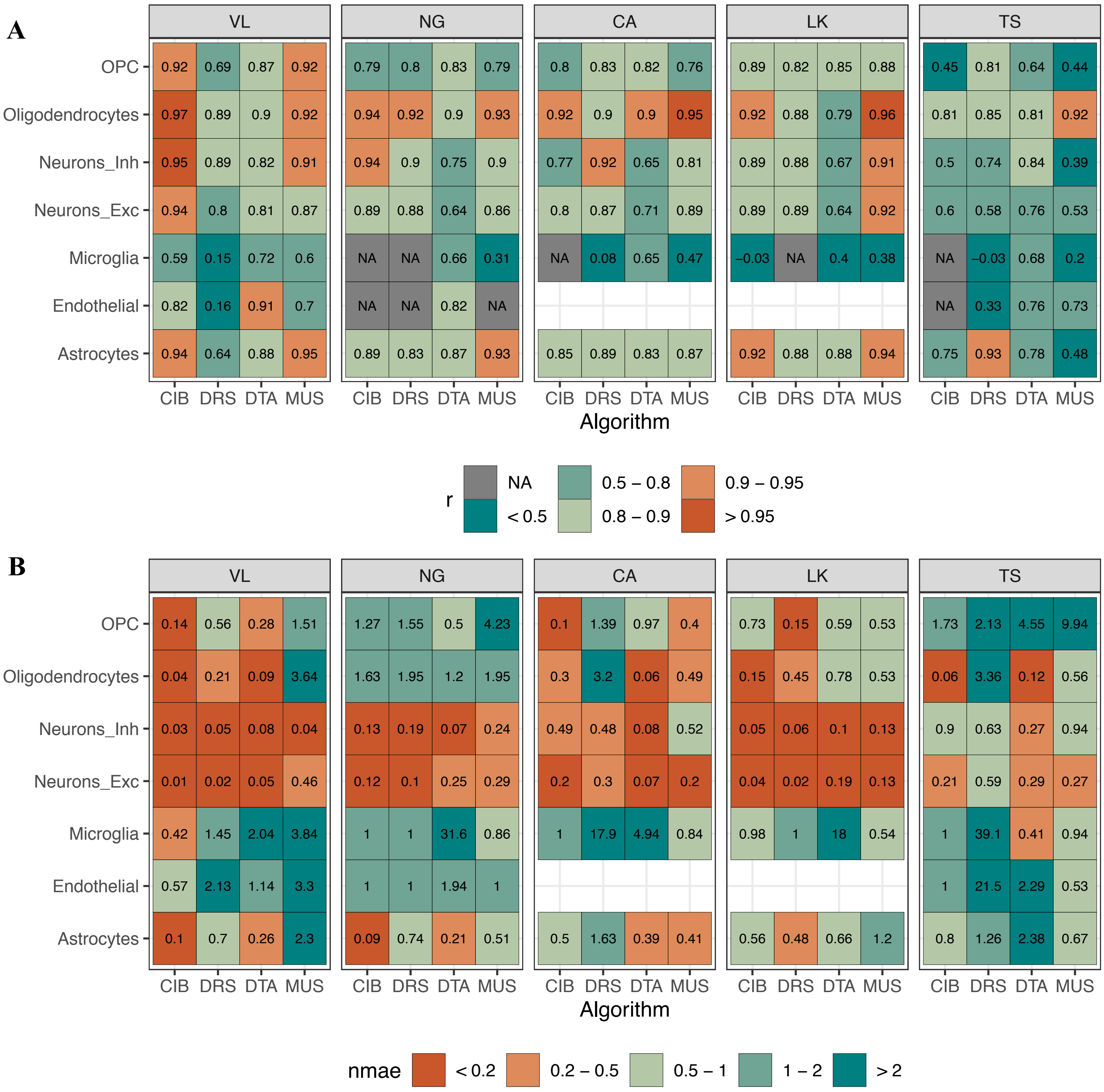

Supplementary Figure 23. Effect of varying the signature while including neuronal subtypes in VL-based simulated mixtures. Heatmaps of Pearson correlation (*r*; **A.**) and normalised mean absolute error (nmae; **B.**) for estimated versus true proportion when varying the reference signature (grouped by grey boxes). Columns represent different algorithms with which the signature was combined, while rows represent different cell-types. Thus, each entry represents the statistic for a cell-type when deconvolution is performed with a given signature/algorithm combination. The mixtures are 100 *in silico* VL simulations. All signatures here include information about the broad neuronal subtype (excitatory or inhibitory). Blank squares indicate that the cell-type was not present in the signature, and thus no statistic was calculated. Grey squares indicate NA, indicating that the cell-type was present in the signature but the statistic could not be calculated; for *r*, this means there was no variance in the composition estimates, typically meaning all 100 samples' estimates were 0 or 1. *Neurons_Inh:* Inhibitory Neurons. *Neurons_Exc:* Excitatory Neurons. *OPC:* Oligodendrocyte Precursor Cells. *CIB*: CIBERSORT. *DRS*: DeconRNASeq. *DTA*: dtangle. *MUS*: MuSiC. For more details about signature characteristics (represented by boxes), see methods.

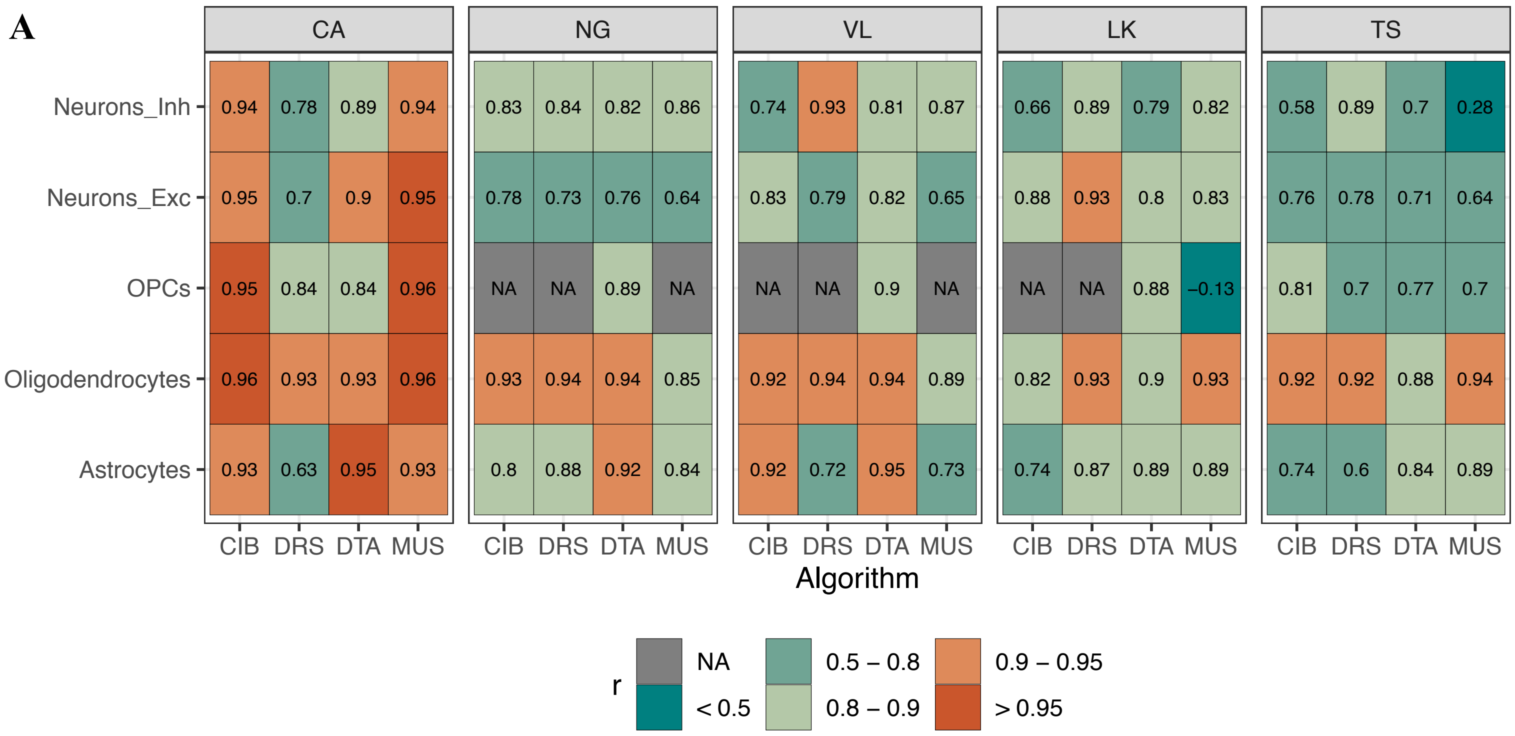

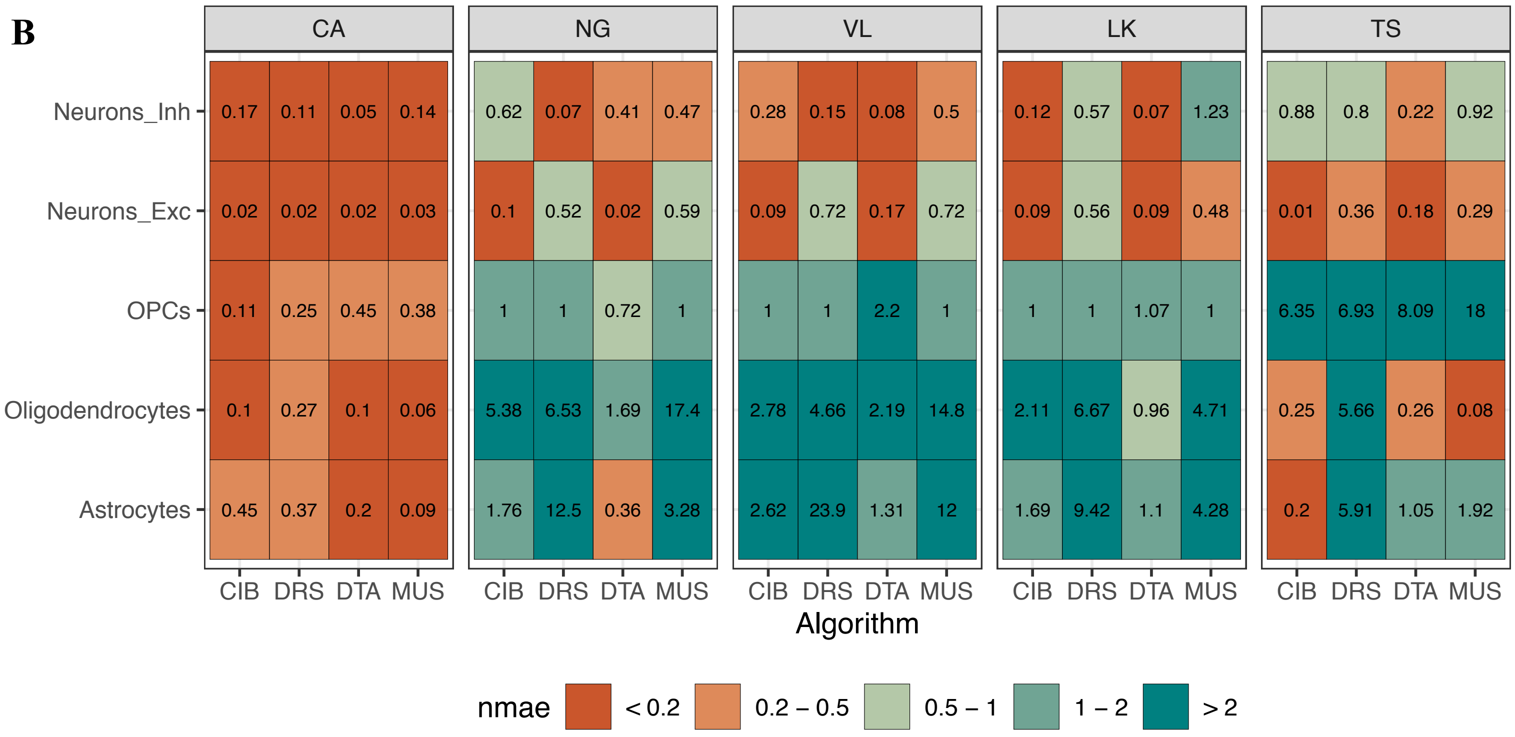

Supplementary Figure 24. Effect of varying the signature while including neuronal subtypes in CA-based simulated mixtures. Heatmaps of Pearson correlation (*r*; **A.**) and normalised mean absolute error (nmae; **B.**) for estimated versus true proportion when varying the reference signature (grouped by grey boxes). Columns represent different algorithms with which the signature was combined, while rows represent different cell-types. Thus, each entry represents the statistic for a cell-type when deconvolution is performed with a given signature/algorithm combination. The mixtures are 100 *in silico* CA-derived simulations. All signatures here include information about the broad neuronal subtype (excitatory or inhibitory). Blank squares indicate that the cell-type was not present in the signature, and thus no statistic was calculated. Grey squares indicate NA, indicating that the cell-type was present in the signature but the statistic could not be calculated; for *r*, this means there was no variance in the composition estimates, typically meaning all 100 samples' estimates were 0 or 1. *Neurons_Inh:* Inhibitory Neurons. *Neurons_Exc:* Excitatory Neurons. *OPCs:* Oligodendrocyte Precursor Cells. *CIB*: CIBERSORT. *DRS*: DeconRNASeq. *DTA*: dtangle. *MUS*: MuSiC. For more details about signature characteristics, see methods.

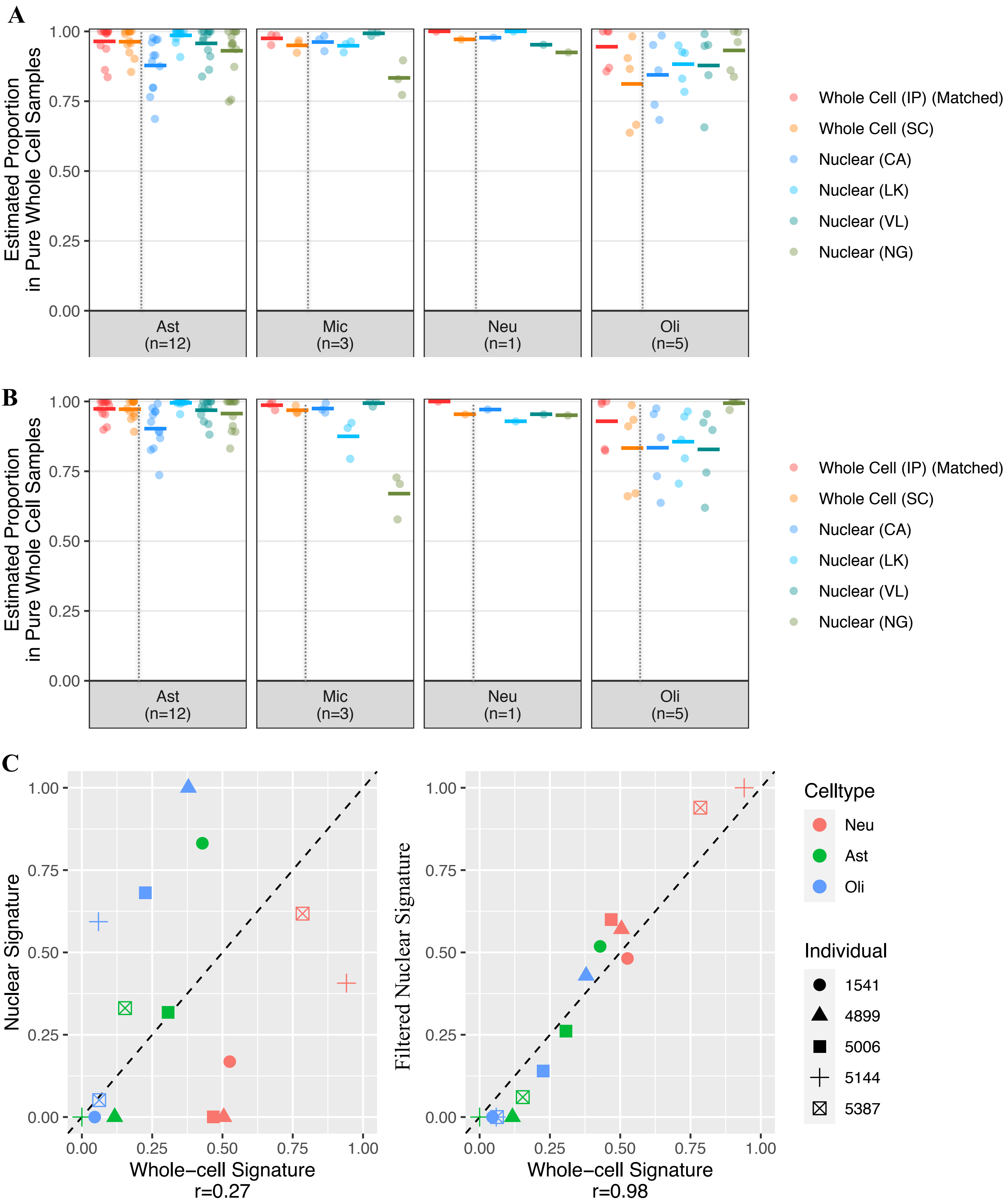

Supplementary Figure 25. The role of compartment-specific genes when using snRNA-seq signatures. A and B. CIBERSORT-estimated proportions for pure samples of immuno-panned cell-types, using whole-cell and snRNA-seq signatures. Signatures are coded by point colour; see Methods for more details about each signature. A. All genes were included in each signature. B. Compartment-specific genes were filtered-out. These 2808 genes were identified using a linear model comparing 5 paired nuclear and whole-cell brain bulk transcriptomes (Benjamini-Hochberg adjusted p-value < 0.05, absolute log2 fold-change > 1.3). *Thick horizontal line*: mean. *Dotted vertical line*: separates whole-cell from snRNA-seq signatures. *Neu*: neurons. *Ast*: astrocytes. *Oli*: oligodendrocytes. *Mic*: microglia. **C.** Scatterplot of CIBERSORT-estimated proportions for bulk brain samples using the RNA-seq signature, IP (x-axis) or the snRNA-seq signature derived from the same individual (y-axis). *Left*: all genes were included in the signature; *right*: compartment-specific genes were filtered-out. *Individual*: NICHD brain bank id number. Cell-type proportions were estimated using CIBERSORT.

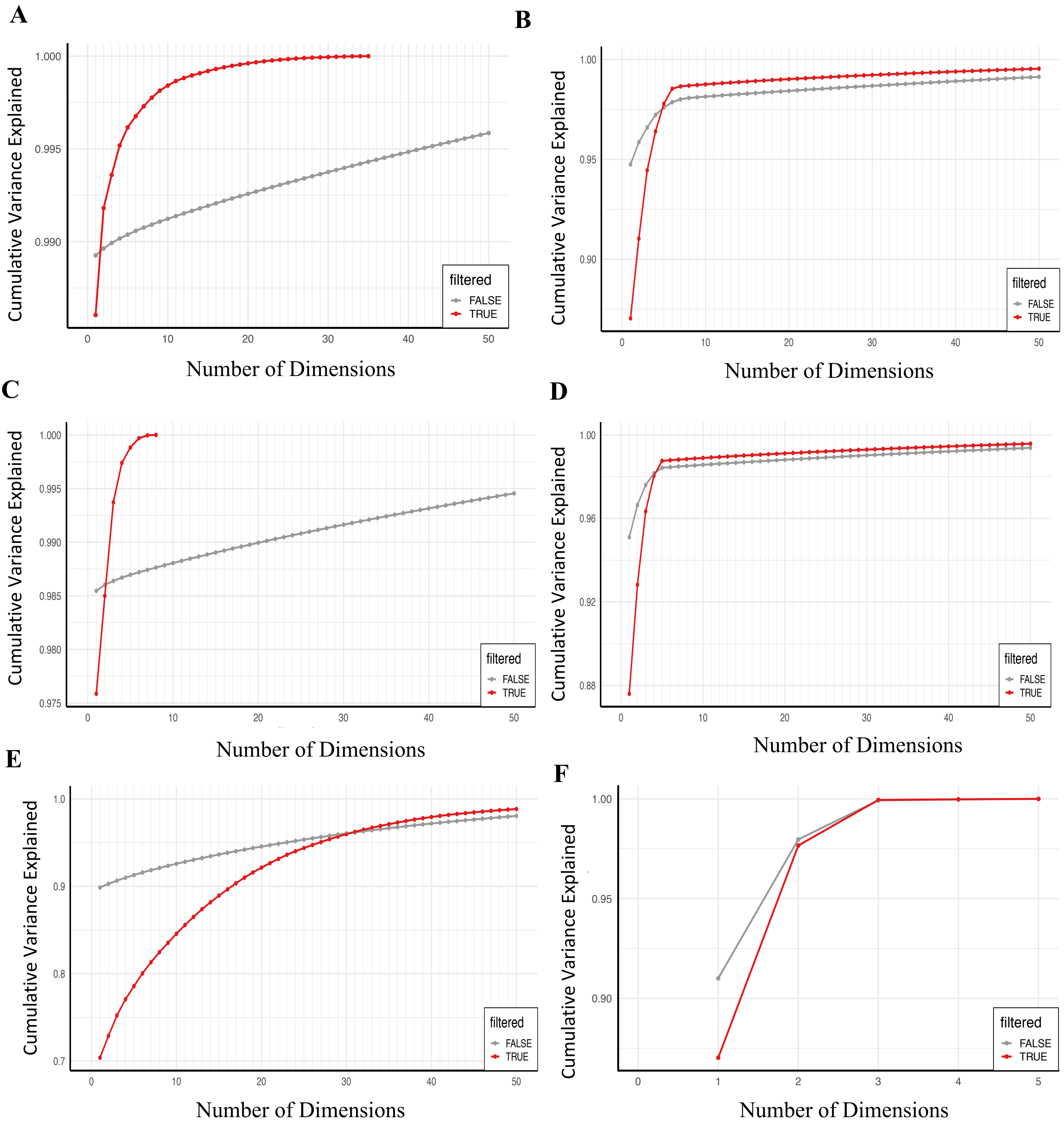

Supplementary Figure 26. Proportion of variance in gene expression explained by singular-value decomposition. Linseed proposes that the saturation point of the curve (*i.e.* the number of linearly-independent components that contribute to the mixture) is its number of constituent cell-types. **A-B.** VL-derived mixtures based on random sampling (A) and those simulated with a wide range and variance in cell-type composition (B). **C-D.** CA mixtures based on random sampling (C) and those simulated with a wide range and variance in cell-type composition (D). **E.** DM mixtures based on random sampling. **F.** RNA mixtures.

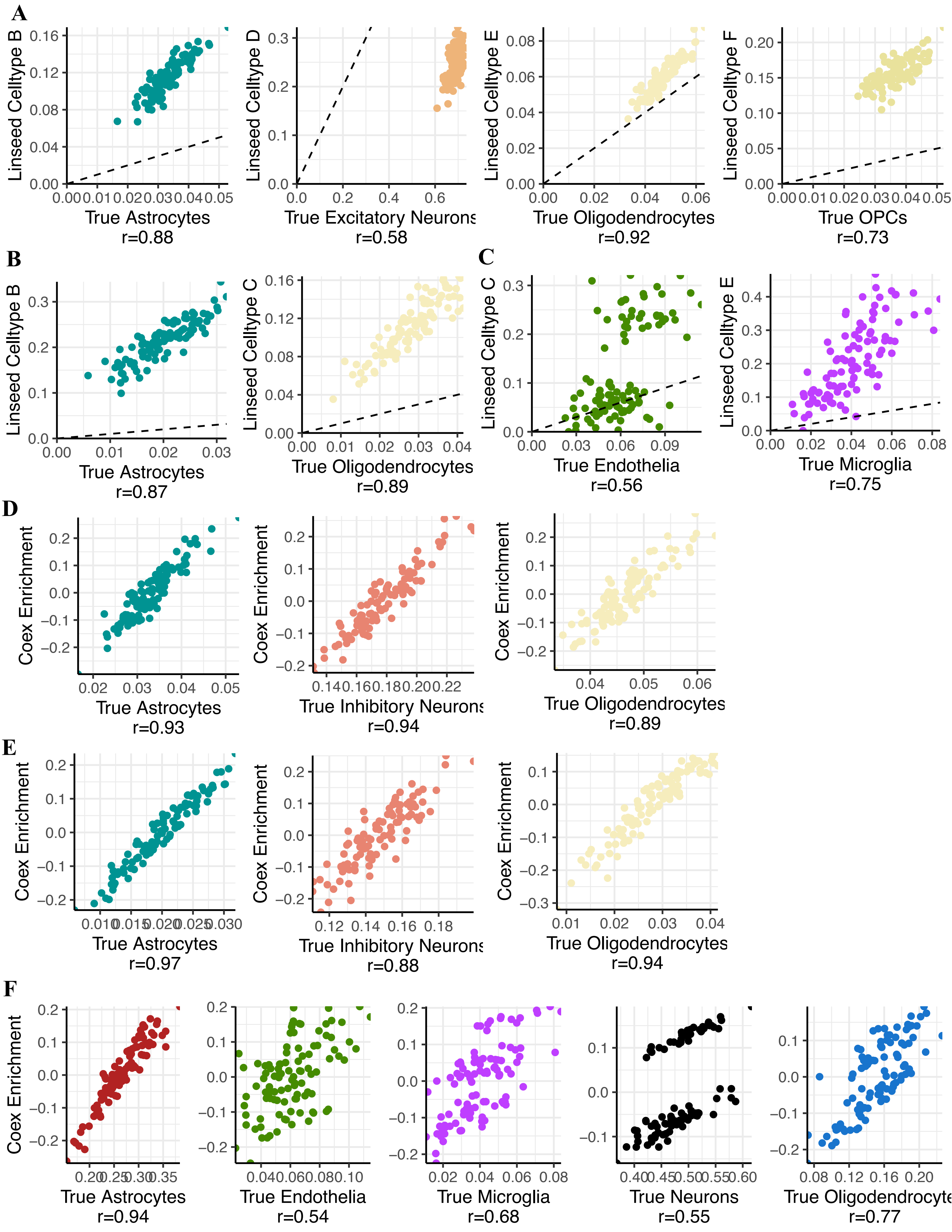

Supplementary Figure 27. Scatterplots of reference-free deconvolution estimates versus true proportion. **A-C.** Application of Linseed to three *in silico* simulated datasets with 100 samples. The parameter *k* was set to the number of known cell-types in the simluations. Plots are only shown for inferred cell-types correlated to a true cell-type at r > 0.5. A. VL-derived random simulations. B. CA-derived random simulations. C. DM-derived random simulations. *Dotted line*: y = x. **D-F.** Coex. Plots are only shown for inferred cell-types that were assigned to a true cell-type through marker enrichment analysis (Fisher Test, $p<1x10^{-5}$, odds ratio > 5; Methods) . D. VL-derived random simulations. E. CA-derived random simulations. F. DM-derived random simulations.

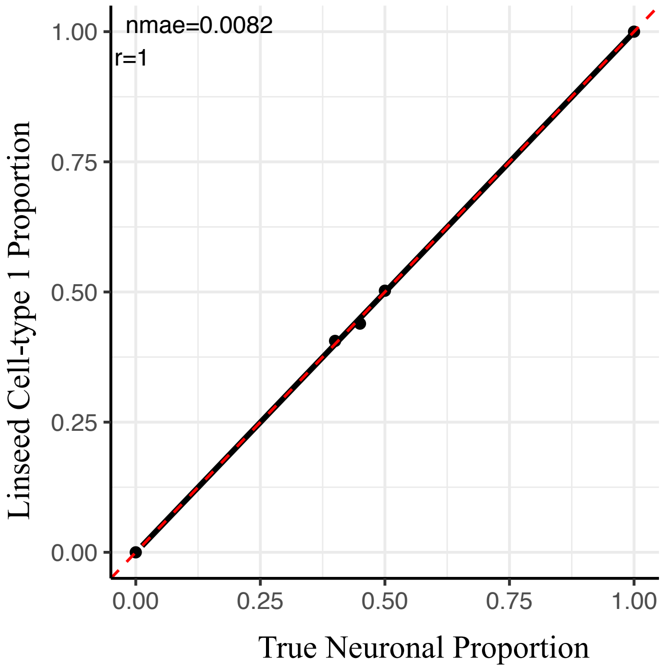

Supplementary Figure 28. Performance of Linseed on RNA mixtures. Scatterplot of proportions estimated by Linseed when applied to 5 mixtures of neuronal and astrocytic RNA (Methods). The number of cell-types was set to *k*=2. *Black line*: regression line. *Red dotted line*: y=x. *nmae*: normalised mean absolute error. *r*: Pearson correlation.

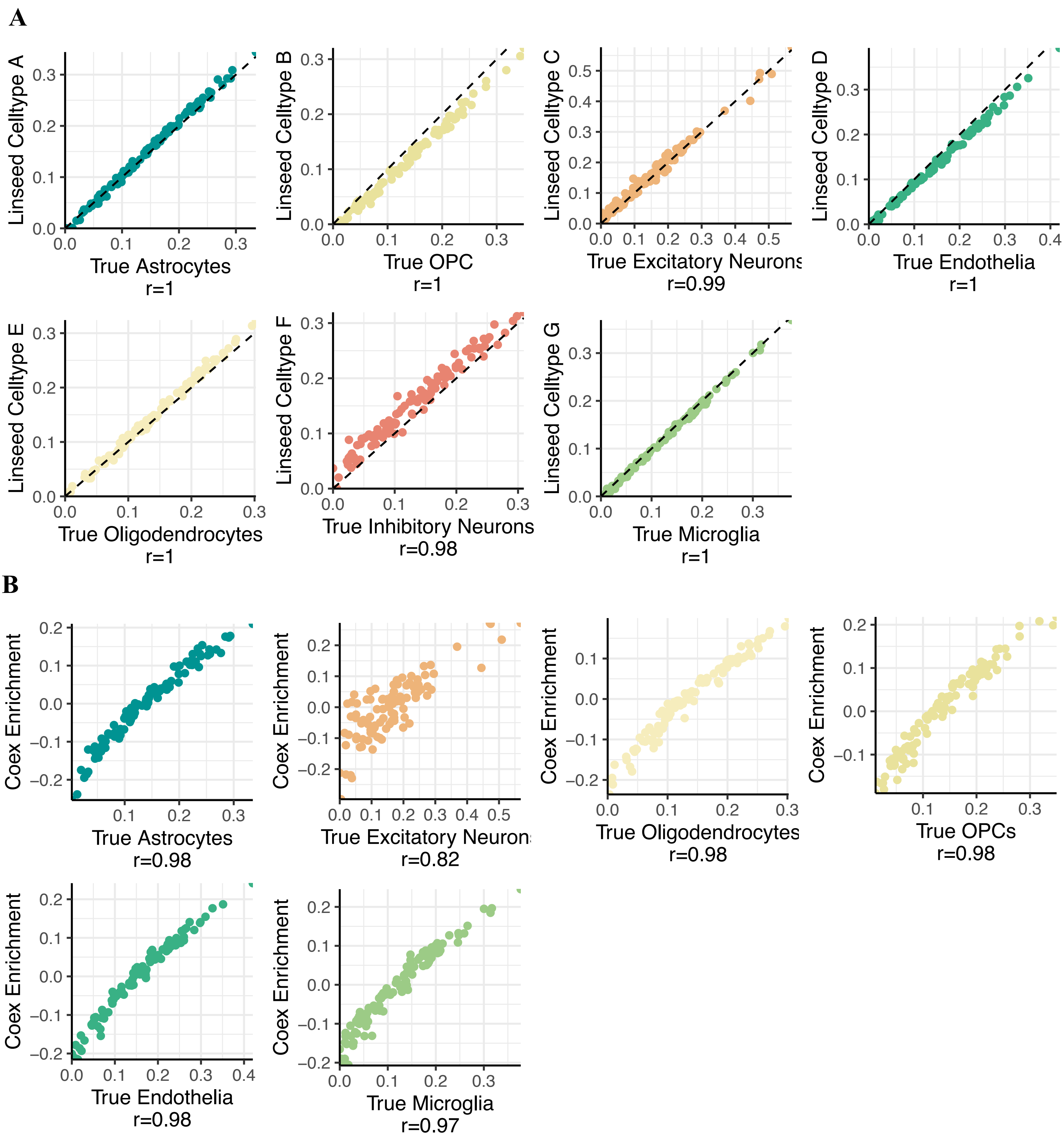

Supplementary Figure 29. Scatterplots of reference-free deconvolution estimates versus true proportion in simulations with increased cell-type variance. Simulations were based on VL single-nuclei. **A.** Linseed. **B.** Coex.

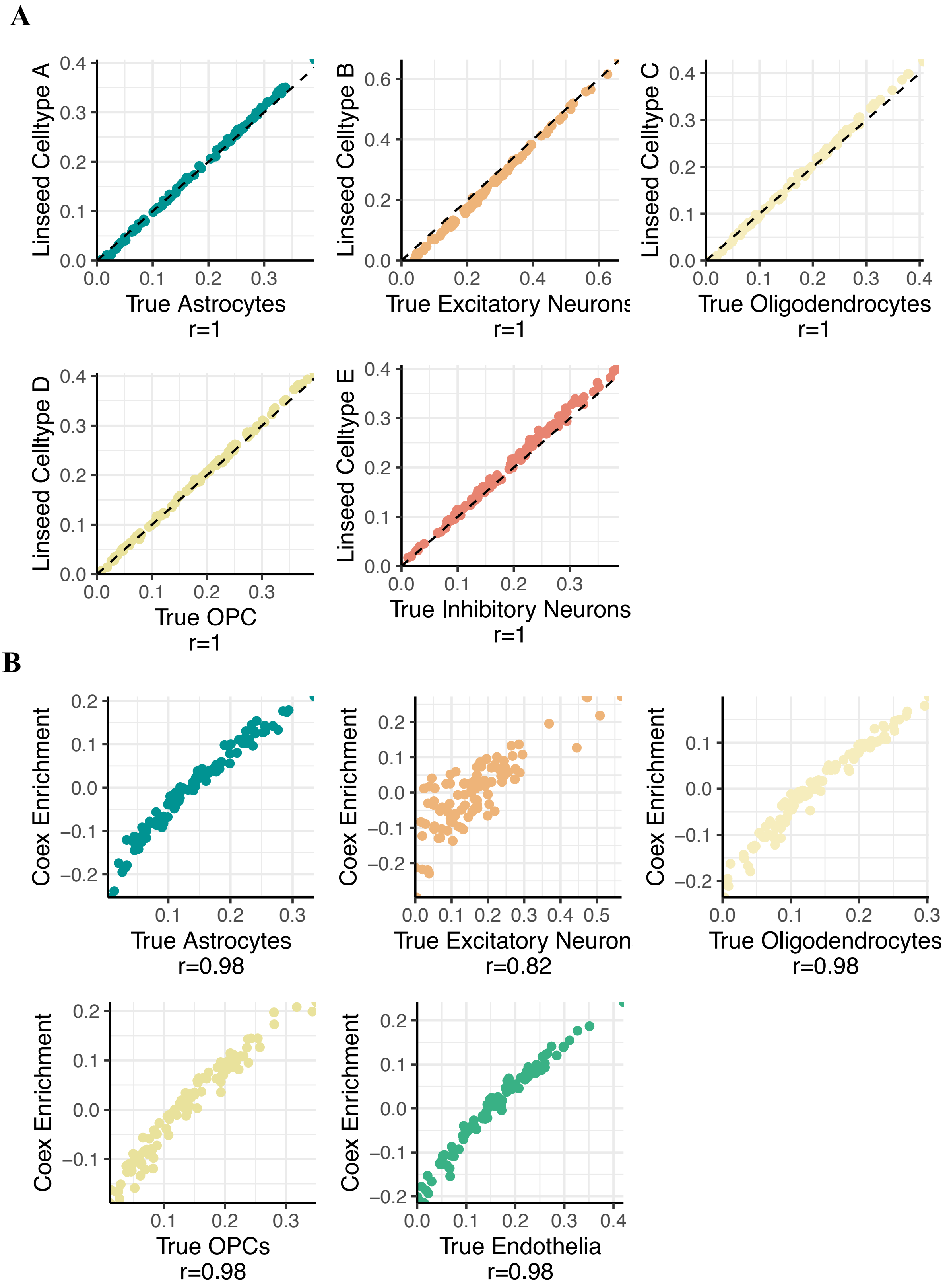

Supplementary Figure 30. Scatterplots of reference-free deconvolution estimates versus true proportion in simulations with increased cell-type variance. Simulations were based on CA single-nuclei. **A.** Linseed. **B.** Coex.

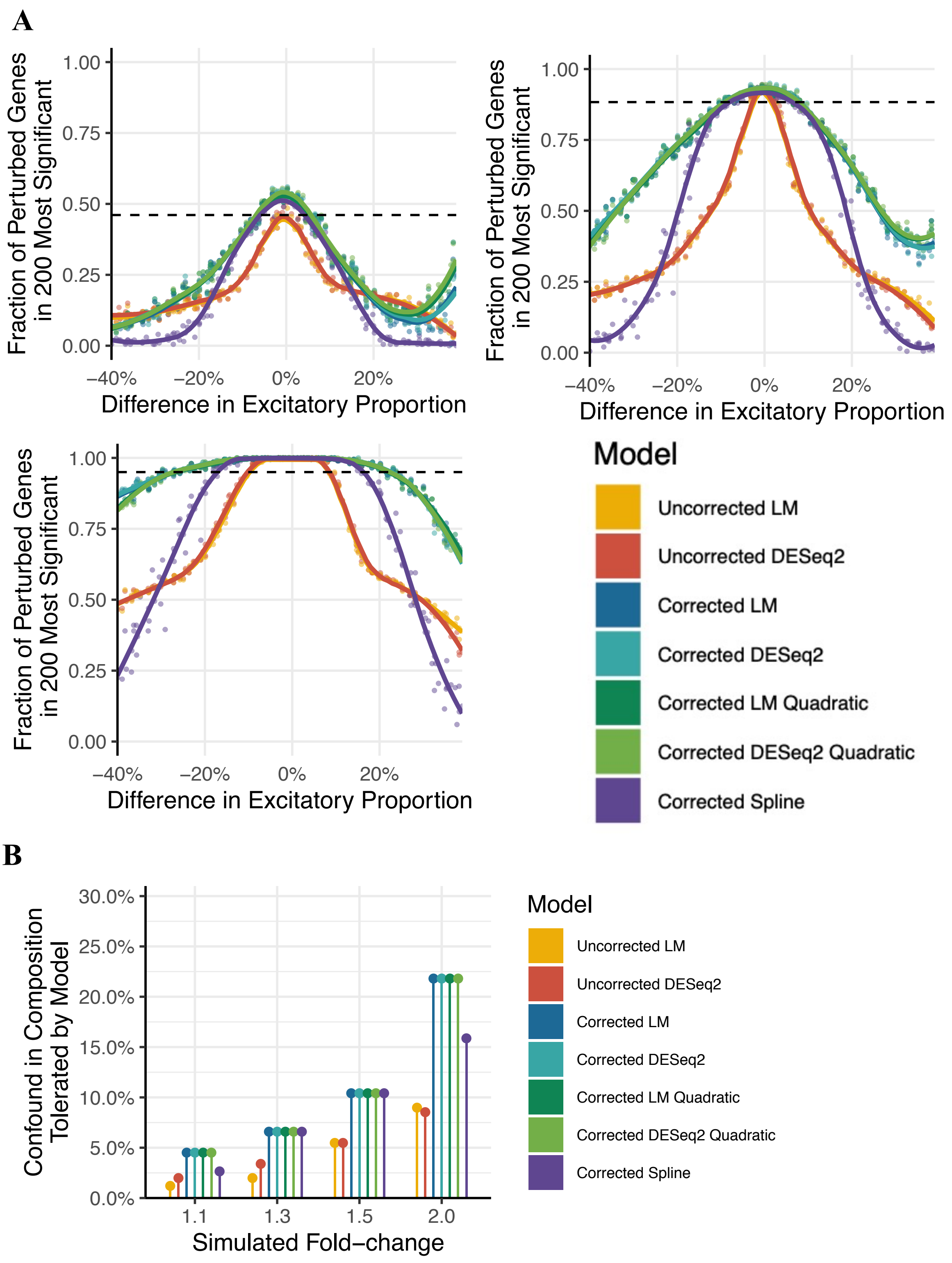

Supplementary Figure 31. Interplay between confounds in excitatory cell-type proportion and differential expression for true up- or down-regulated genes. **A.** Scatterplots show the relationship between the confound in excitatory proportion within a simulation, and the discriminatory ability (fraction of 200 known perturbed genes in the 200 genes with the smallest p-value). Each point represents the value from a simulated dataset with two groups of 50 samples. Each colour represents a different model for differential expression (methods). *Top-left*: true fold-change of 1.1. *Top-right*: true fold-change of 1.3. *Bottom-left*: true fold-change of 2. *Coloured lines*: local regression line. *Dotted line in the left* panel: 0.95 times the discriminatory ability for LM when x = 0. *LM*: linear model. **B.** Model robustness to composition confounds across fold-changes. Barplots show the smallest negative composition change where discriminatory ability fell below 0.95 of the expected value (*i.e.* that from an uncorrected linear model on a simulation with no composition confound). Labels are per A.

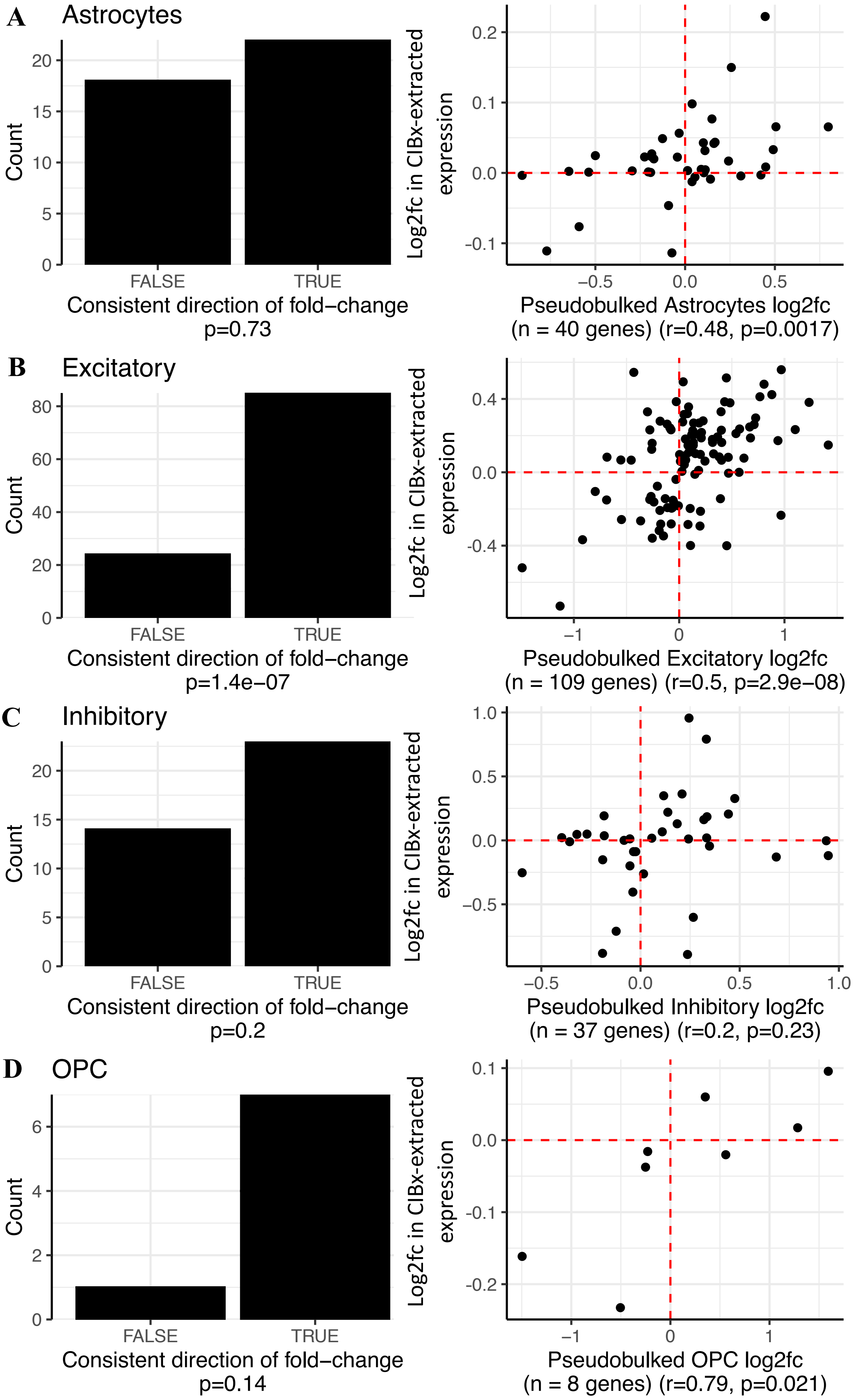

Supplementary Figure 32. Performance of CIBERSORTx on pseudo-bulked Autism and Control Single Nucleus Data. A-D. Single-nuclei for four cell-types were accessed for 15 Autism Spectrum Disorder (ASD) and 11 control individuals from Velmeshev *et al.* (2019) (Methods). Nuclei were pseudo-bulked for each individual, for each cell-type separately, or for all nuclei together regardless of cell-type. The latter was used as input for CIBERSORTx to extract cell-type-specific expression for the 250 genes with the lowest p-value for ASD, though only a subset passed filtering (Methods). All differential expression was performed using a linear model on log2 transformed counts-per-million data. *Left panel*: barplot of the number of genes with consistent directions of fold-change, with p-values calculated using a Fisher's Exact Test. *Right panel*: scatterplot of the log2 fold-change in the pseudo-bulked single-nuclei of the given cell-type (x-axis) versus the fold-change from extracted expression (y-axis). **A.** Astrocytes. **B.** Excitatory Neurons. **C.** Inhibitory Neurons. **D.** Oligodendrocyte Precursor Cells (OPC). *CIBx*: CIBERSORTx. *N*: the number of genes. *Log2fc*: log2 of the fold-change between ASD and control samples. *r*: Pearson correlation.

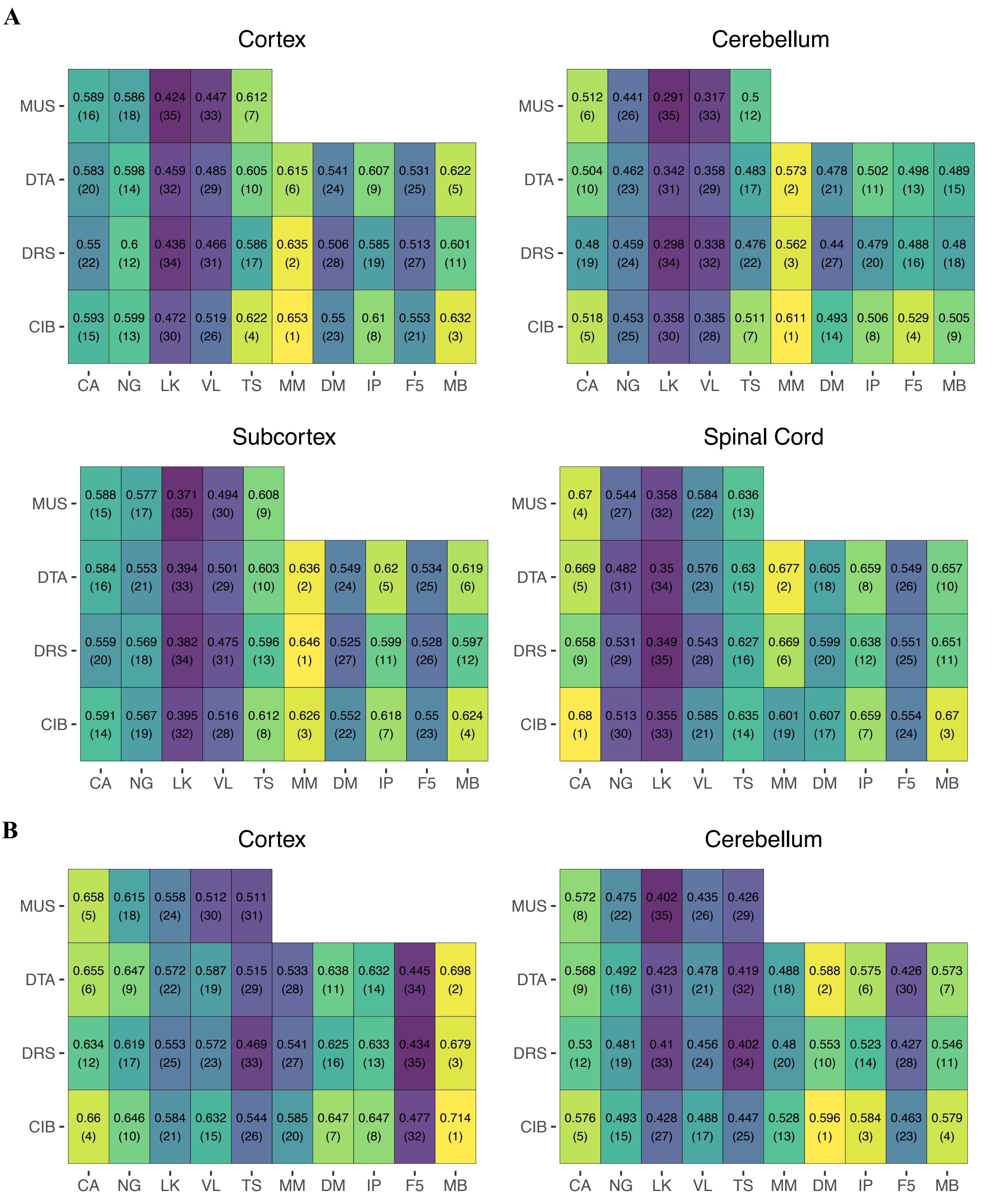

Supplementary Figure 33. Median goodness-of-fit in large bulk brain RNA-seq datasets across signatures and algorithms. A. GTEx. **B.** Parikshak *et al.*. Each panel aggregates results from samples from a given region. Rows represent signatures, and columns represent algorithms. Within each cell, the number of top is the median goodness-of-fit, while the number in parentheses below is its rank across all algorithm/signature combinations. Colours represent rank, ranging from purple (worst performance and high rank) to yellow (best performance and low rank). *CIB*: CIBERSORT. *DRS*: DeconRNASeq. *DTA*: dtangle. *MUS*: MuSiC.

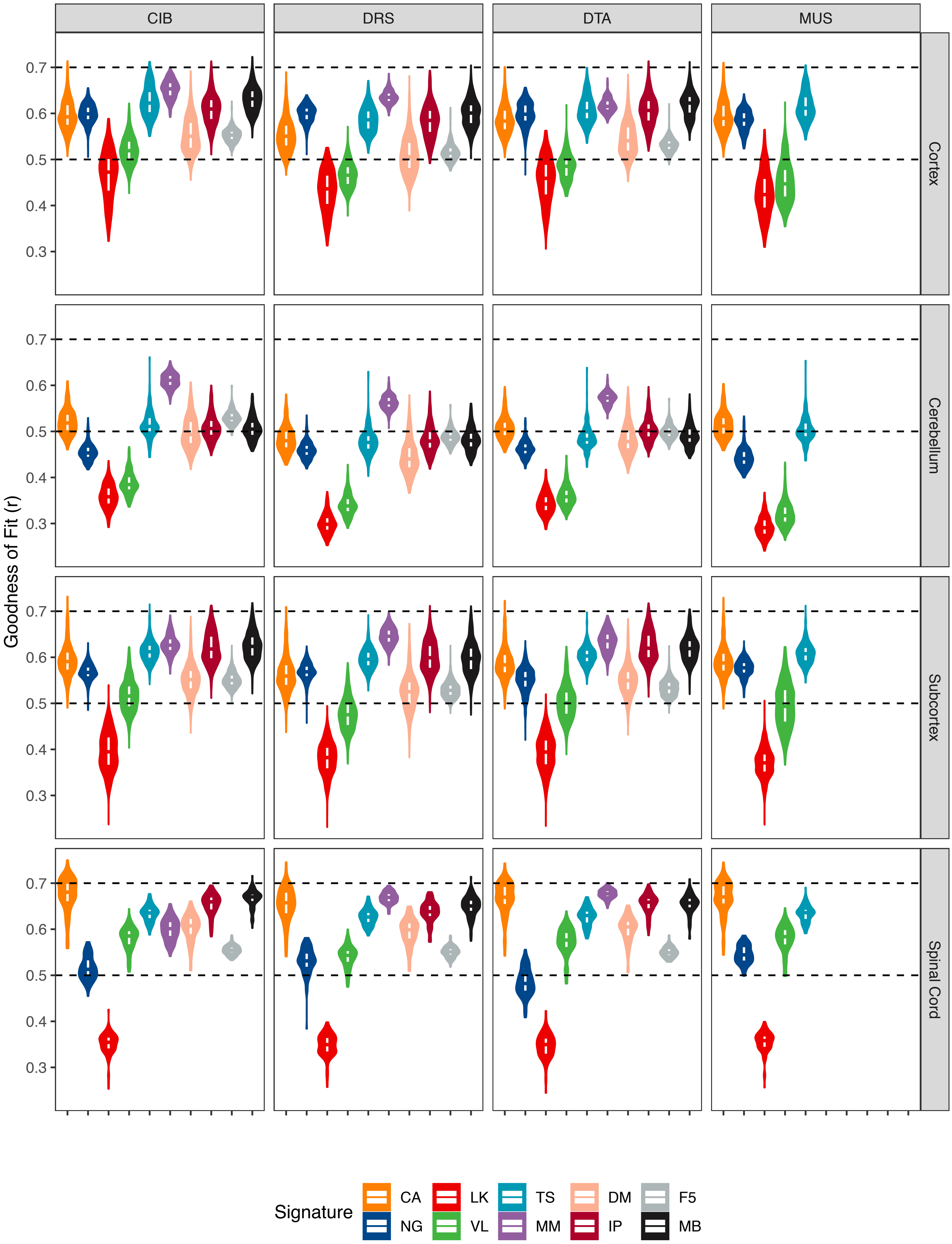

Supplementary Figure 34. Violin plots of goodness of fit across signatures and regions in GTEx data. Columns show four algorithms, while rows show four brain regions (n=408, 309, 863, and 91 for cortex, cerebellum, subcortex, and spinal cord, respectively). Colours represent different signatures, for which further information can be found in the methods. The top, middle, and bottom of the white internal boxes mark the 75th, 50th, and 25th percentiles, respectively, while the width of the coloured violin represents the distribution of points. Note that the data presented in the top-left panel (CIB/Cortex) was also shown in Figure 6A. *CIB*: CIBERSORT. *DRS*: DeconRNASeq. *DTA*: dtangle. *MUS*: MuSiC.

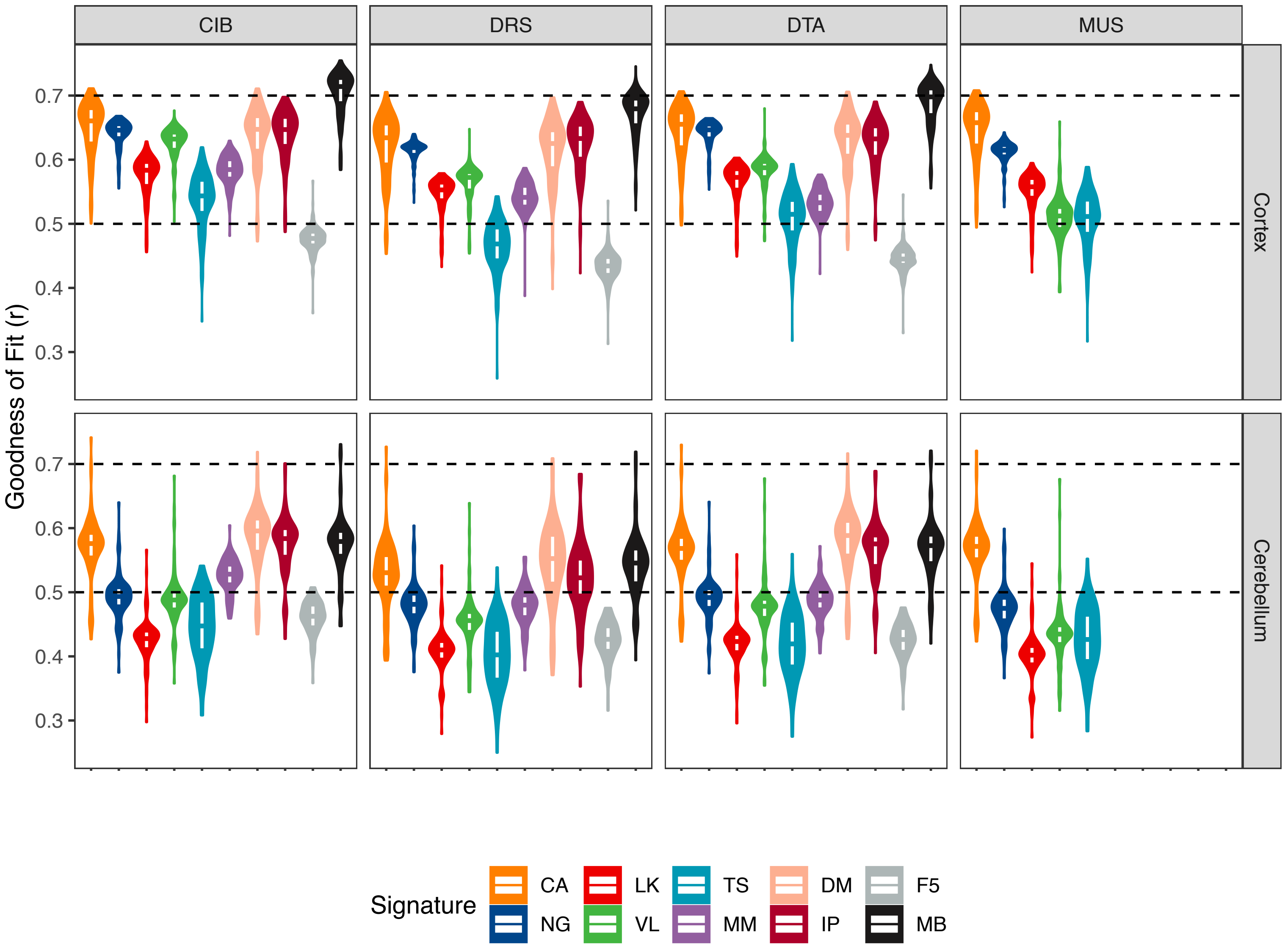

Supplementary Figure 35. Violin plots of goodness of fit across signatures and regions in the Parikshak dataset. Columns show four algorithms, while rows show two brain regions (n=167 and 84 for the cortex and cerebellum, respectively). Colours represent different signatures, for which further information can be found in the methods. The top, middle, and bottom of the white internal boxes mark the 75th, 50th, and 25th percentiles, respectively, while the width of the coloured violin represents the distribution of points. Note that the data presented in the top-left panel (CIB/Cortex) was also shown in Figure 6B. *CIB*: CIBERSORT. *DRS*: DeconRNASeq. *DTA*: dtangle. *MUS*: MuSiC.

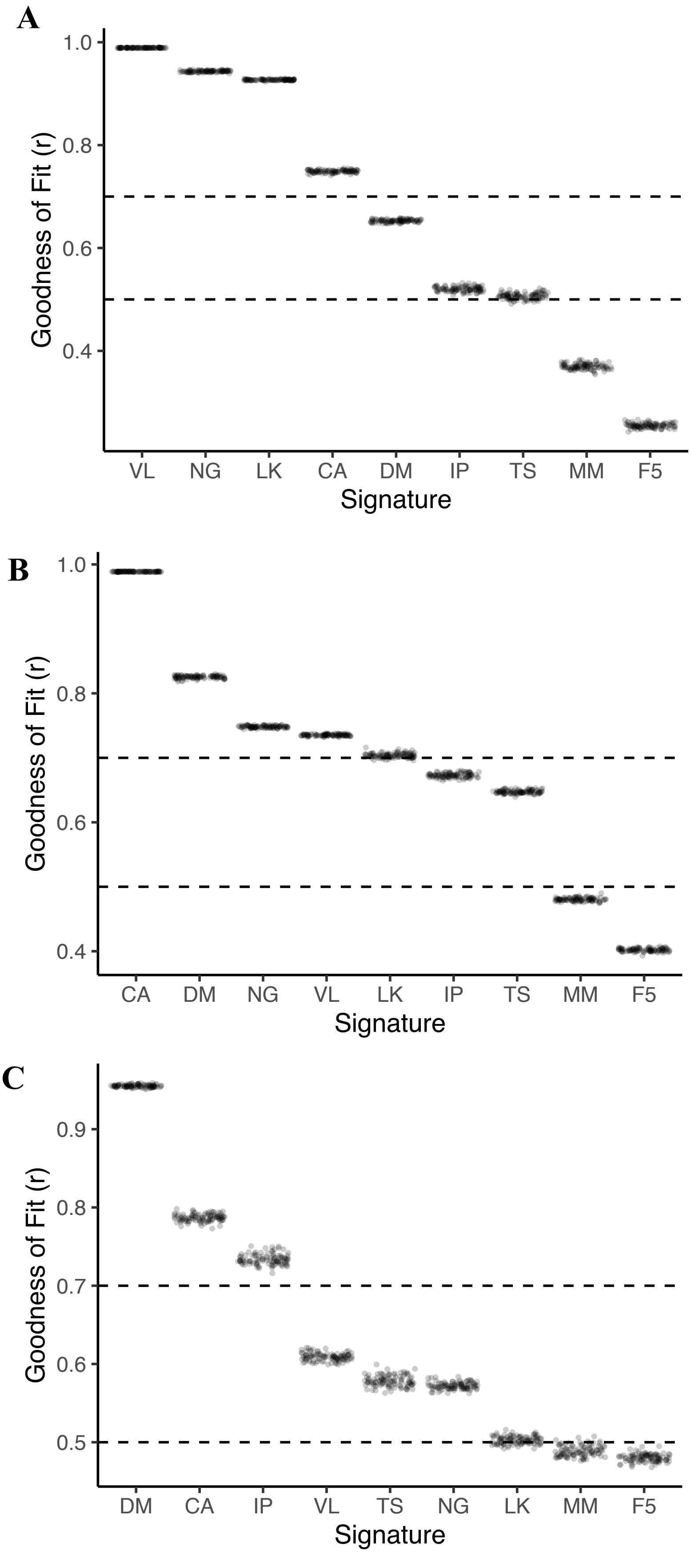

Supplementary Figure 36. Goodness-of-fit across signatures in simulated data. Goodness-of-fit was calculated after deconvolving each mixture in the dataset (n=100) with CIBERSORT and the given signature. Each point represents the goodness-of-fit of a single simulated mixture. **A.** VL-based simulation. **B.** CA-based simulation. **C.** DM-based simulation. Note that the order of signatures along the x-axis is based its median within that panel, and therefore differs between panels. *Dotted horizontal lines:* y = 0.5 and 0.7.

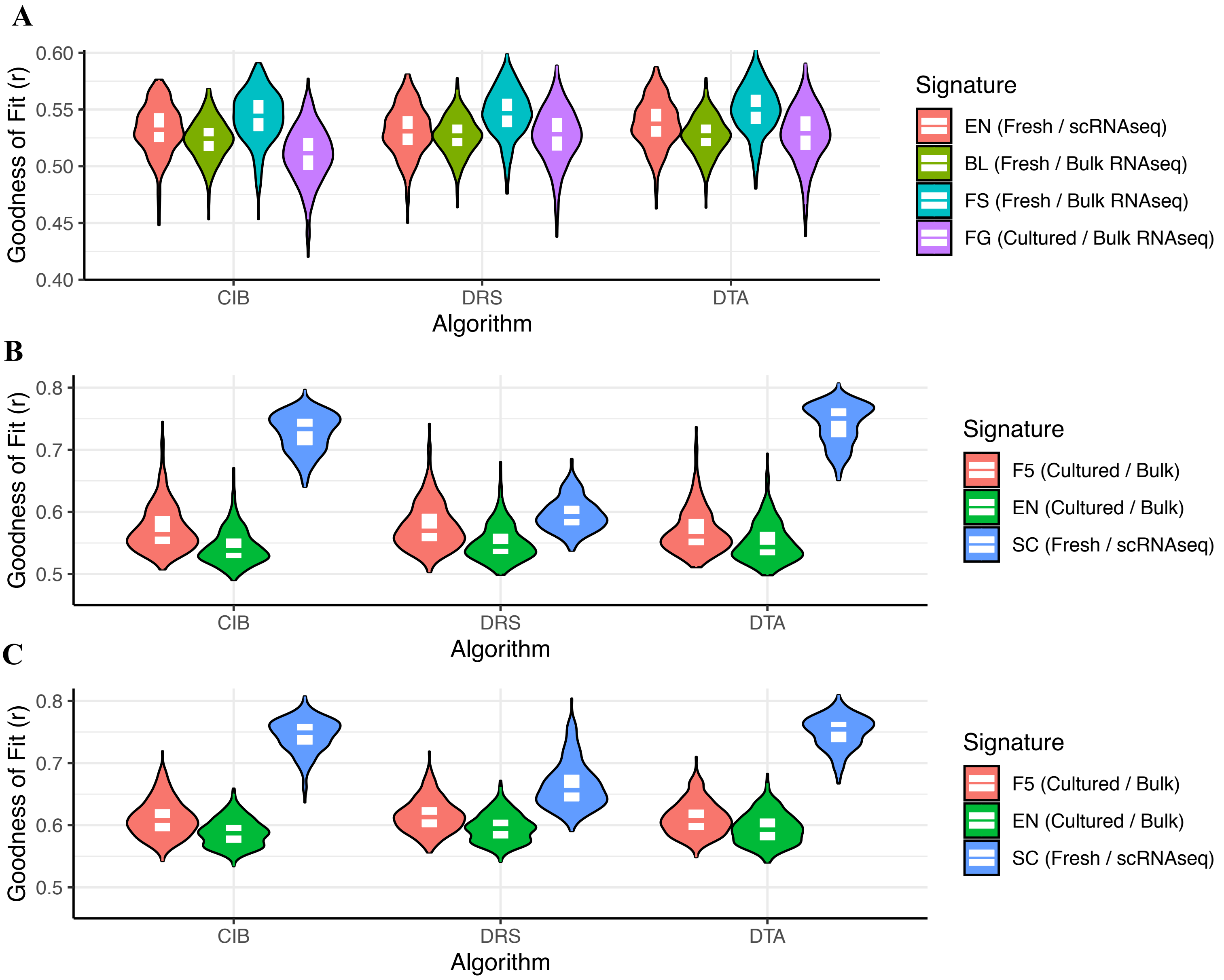

Supplementary Figure 37. Violin plots of goodness-of-fit in two non-brain tissues in the GTEx dataset. **A.** Pancreas samples (n=268). **B.** Heart left ventricle samples (n=417). **C.** Heart atrial appendage samples (n=310). The bottom, middle, and top of the white boxes mark the first, second, and third quantiles, respectively, while the width of the coloured violin represents point density. *CIB*: CIBERSORT. *DRS*: DeconRNASeq. *DTA*: dtangle. Note that the respective legend summarises relevant information about the signature. See methods for full details about each signature. *Fresh:* signature derived from freshly-processed human tissue. *Cultured:* signature derived from cultured cells. *Bulk:* sequencing was performed on a bulk of cells.

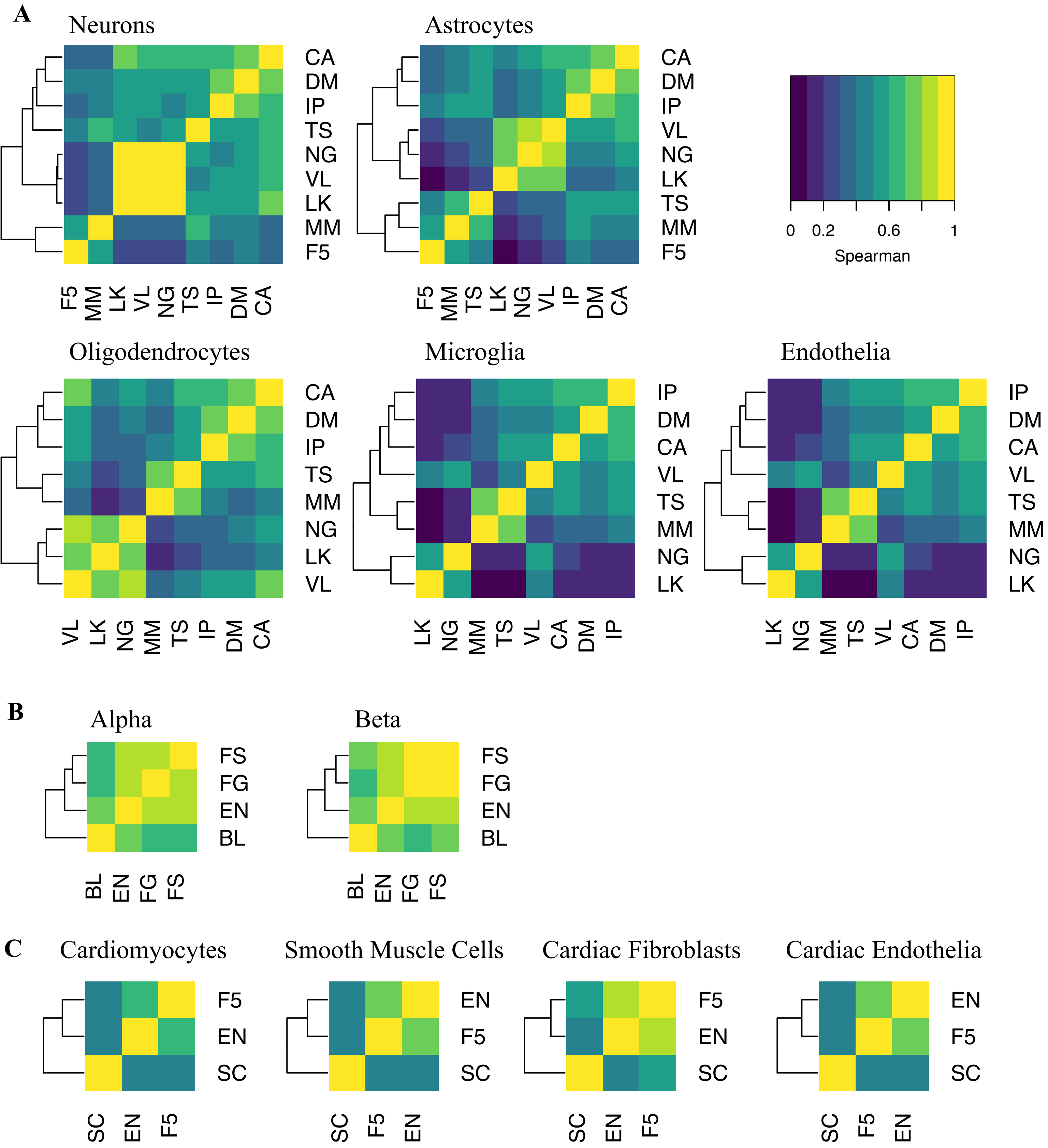

Supplementary Figure 38. Heatmaps of Spearman correlations across signatures. Colours represent the spearman correlation in global gene expression between the signature on the row and on the column. See methods for further details about each signature. **A.** Across nine brain signatures. *Top left*: Neurons. *Top middle*: Astrocytes. *Top right*: legend. *Bottom left*: Oligodendrocytes. *Bottom middle*: Microglia. *Bottom right*: Endothelia. **B.** Across four pancreas signatures. *Left*: alpha cells. R*ight*: beta cells. Legend is per the top right panel of A. **C.** Across three heart signatures. *From left to right*: cardiomyocytes, smooth muscle cells, cardiac fibroblasts, and endothelial cells. Legend is per the top right panel of A.

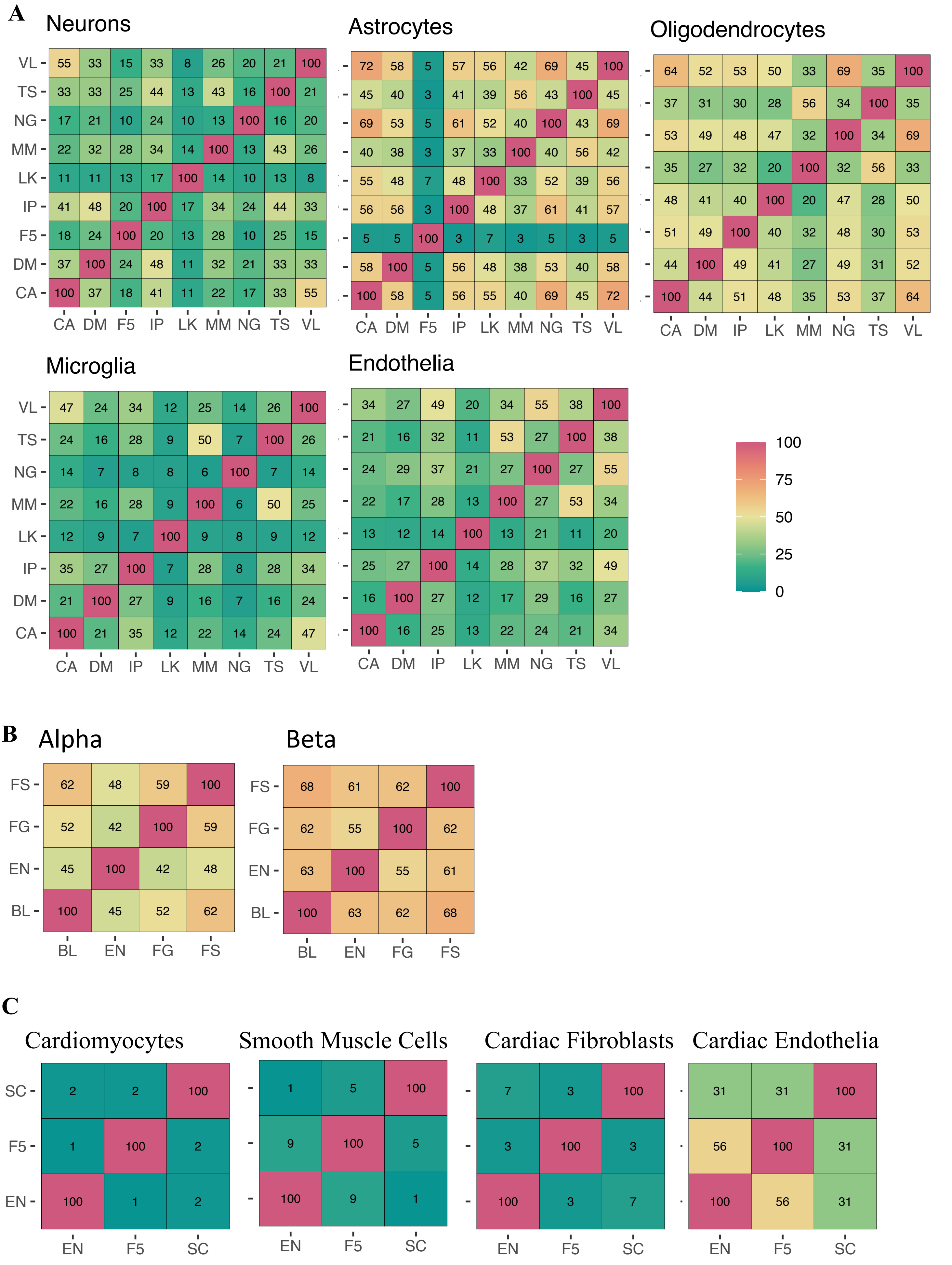

Supplementary Figure 39. Heatmaps of intersection between the top 100 cell-type marker genes across signatures. Numbers and colours indicate the number of common genes in the top markers across signatures. **A.** Across nine brain signatures. **B.** Across four pancreas signatures. Legend is per the bottom right panel of A. **C.** Across three heart signatures. Legend is per the bottom right panel of A.

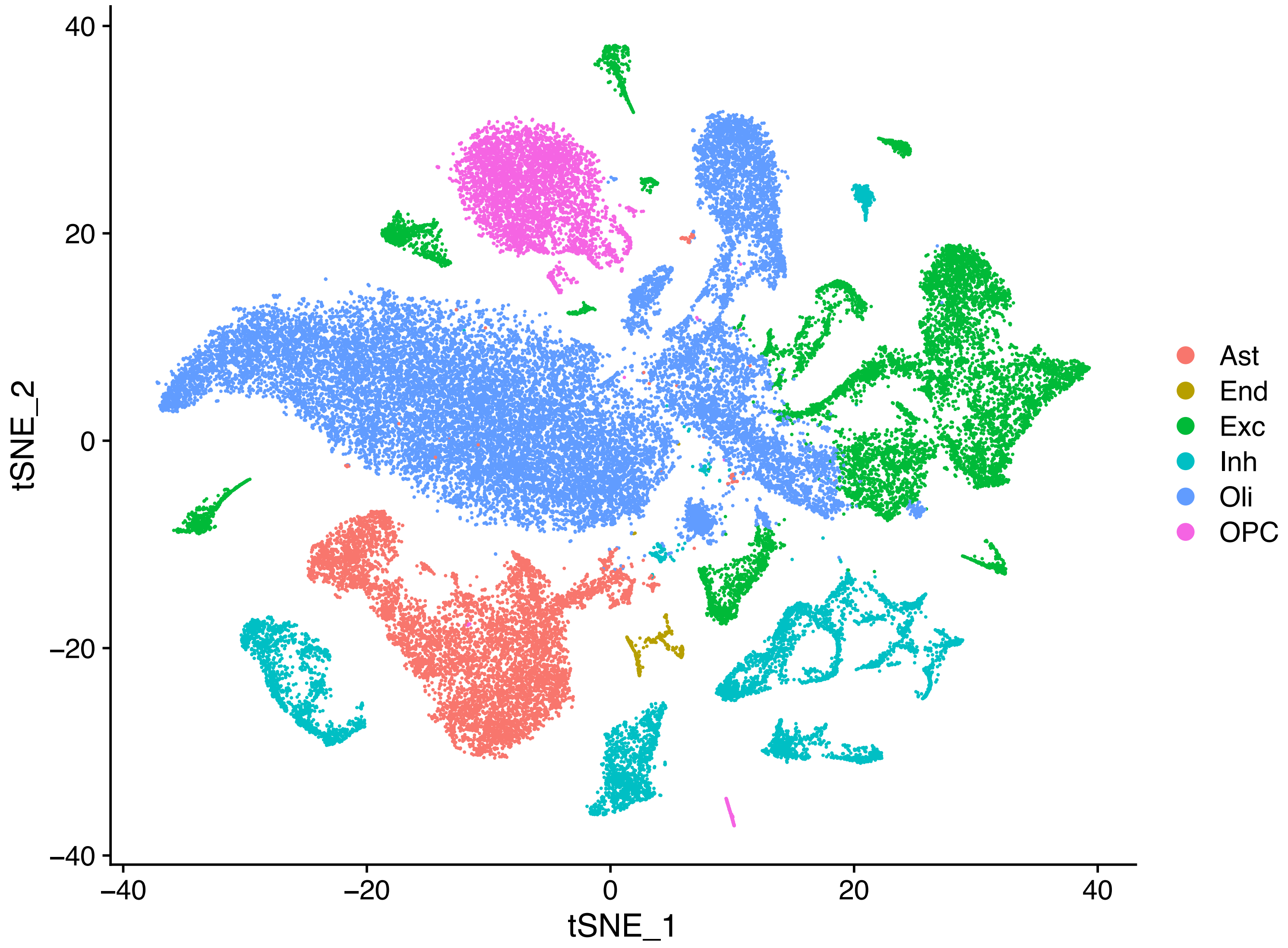

Supplementary Figure 40. tSNE dimensionality reduction plot of snRNA-seq data generated as part of the present study. Nuclei were annotated using the SingleR package to transfer labels from the NG signature. *Ast*: astrocytes. *End*: endothelia. *Exc*: excitatory neurons. *Inh*: inhibitory neurons. *Oli*: oligodendrocytes. *OPC*: oligodendrocyte precursor cells.

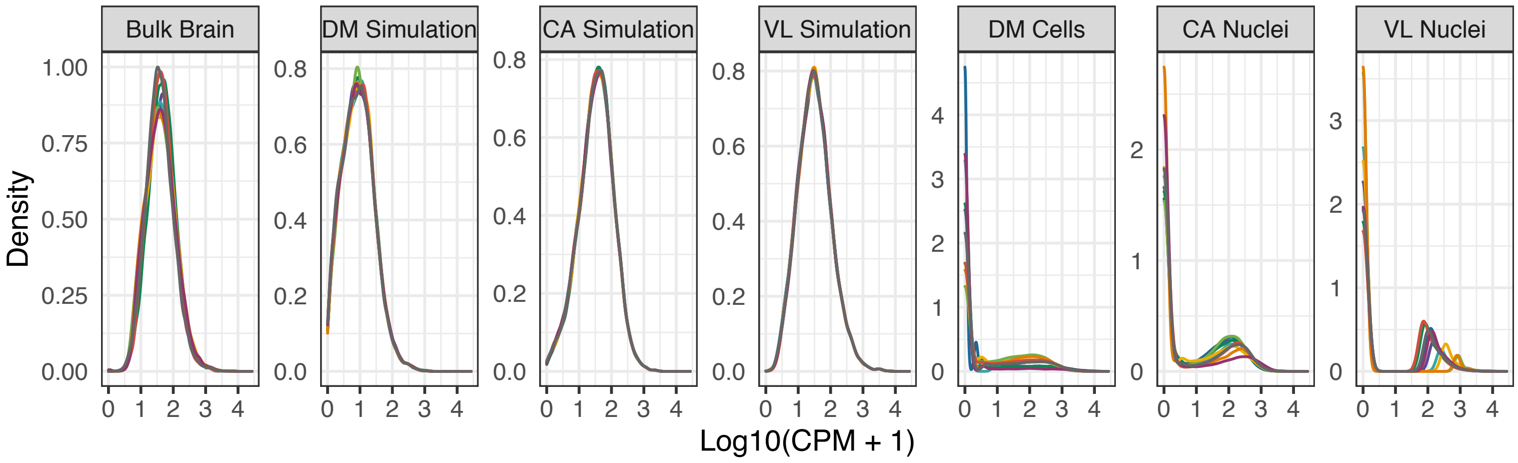

Supplementary Figure 41. Distribution of gene expression values in real and simulated brain mixtures. Each coloured line represents the distribution of log expression across all genes in a given sample. Ten samples were randomly selected for each plot to minimise overplotting. *Brain*: bulk brain RNA-seq from Parikshak et al. (2016). *Simulations*: *in silico* mixtures simulated from the corresponding dataset. *DM*: scRNA-seq from Darmanis *et al.*. *CA*: snRNA-seq data from the Human Cell Atlas. *VL*: snRNA-seq data from Velmeshev *et al.* (2019). Simulations contained 500 cells/mixtures for CA and VL, and 100 cells/mixture for DM. *Nuclei, Cells*: single-nuclei and single-cells from the corresponding dataset. Note: DM simulation is in units of RPKM rather than CPM.

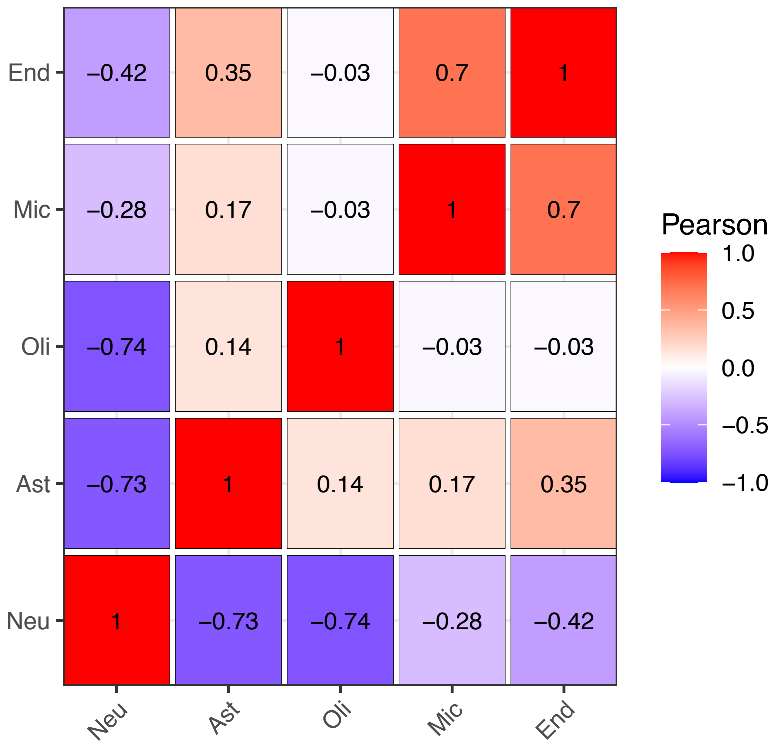

Supplementary Figure 42. Heatmap of Pearson correlations for cell-type proportion in samples used for ASD analyses. Proportions were estimated using CIBERSORT and the Multibrain signature. *Neu*: Neurons. *Ast*: Astrocytes. *Oli*: Oligodendrocytes. *Mic*: Microglia. *End*: Endothelia.

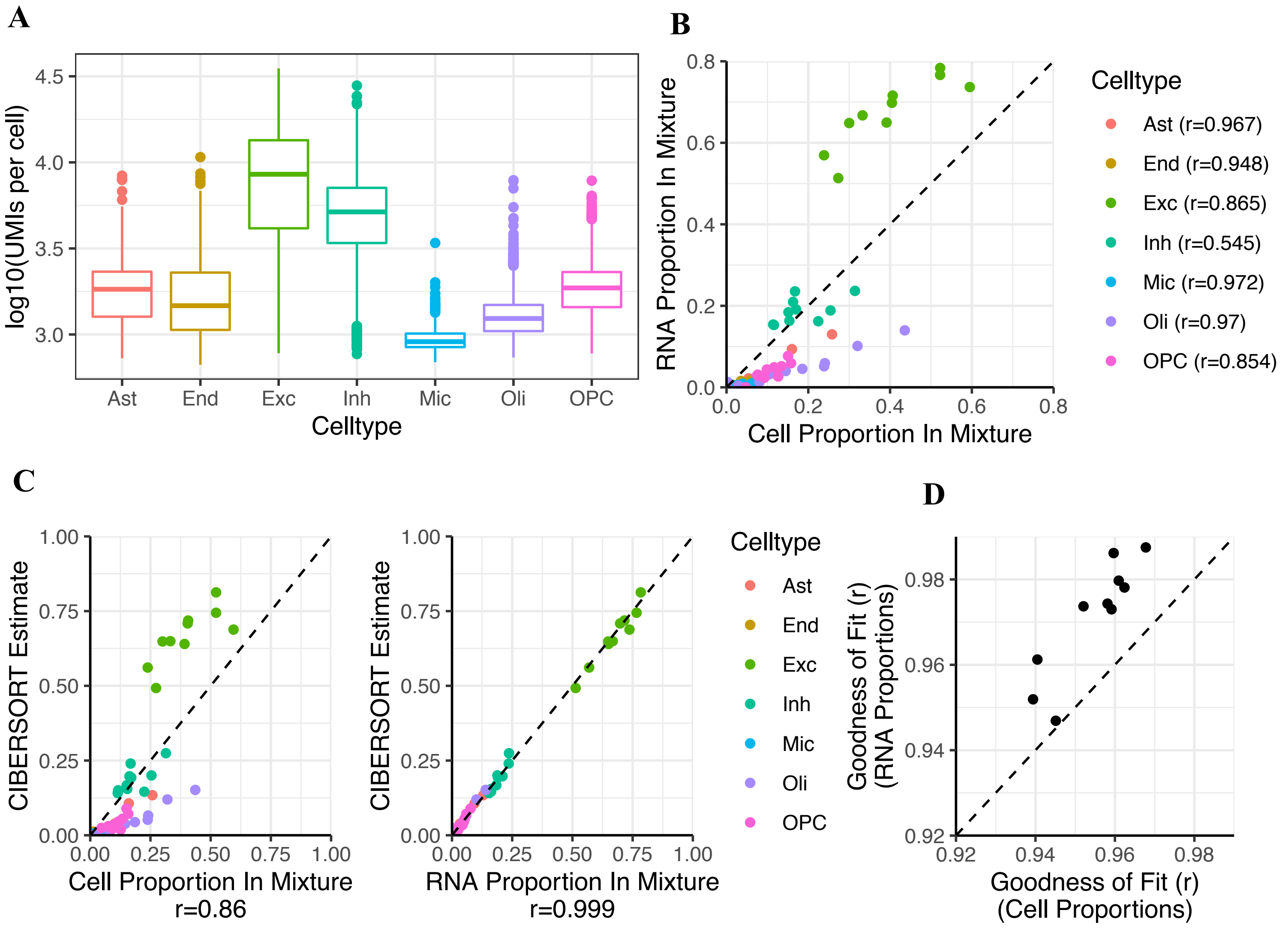

Supplementary Figure 43. The relationship between deconvolution estimates, RNA proportions (pRNA) and cell-type proportion (pCt). A. Boxplots of RNA content per cell, reflected in the number of unique molecular identifiers (UMIs) per-cell across cell types in the VL dataset. The top, middle, and bottom of each box mark the 75th, 50th, and 25th percentile of the data, respectively. Whiskers extend up or down from the box to the maximum or minimum of the data, respectively, up to 1.5x the interquartile range; outliers beyond this range are plotted individually. *Ast:* astrocytes (n=2229). *End:* Endothelia (n=523). *Exc:* Excitatory Neurons (n=9718). *Inh*: Inhibitory Neurons (n=4238). *Mic*: Microglia (n=450). *Oli*: Oligodendrocytes (n=4721). *OPC*: Oligodendrocyte Precursor Cells (n=2677). **B.** Scatterplot of pCt *vs.* pRNA in pseudo-bulk samples from 10 individuals in the VL dataset. **C.** Scatterplot of Estimated proportion (y-axis) versus true pCt (left) or pRNA (right)**. D.** Scatterplot of goodness-of-fit when reconstructing gene expression using pCt (x-axis) or pRNA (y-axis). *All dotted black lines:* y=x

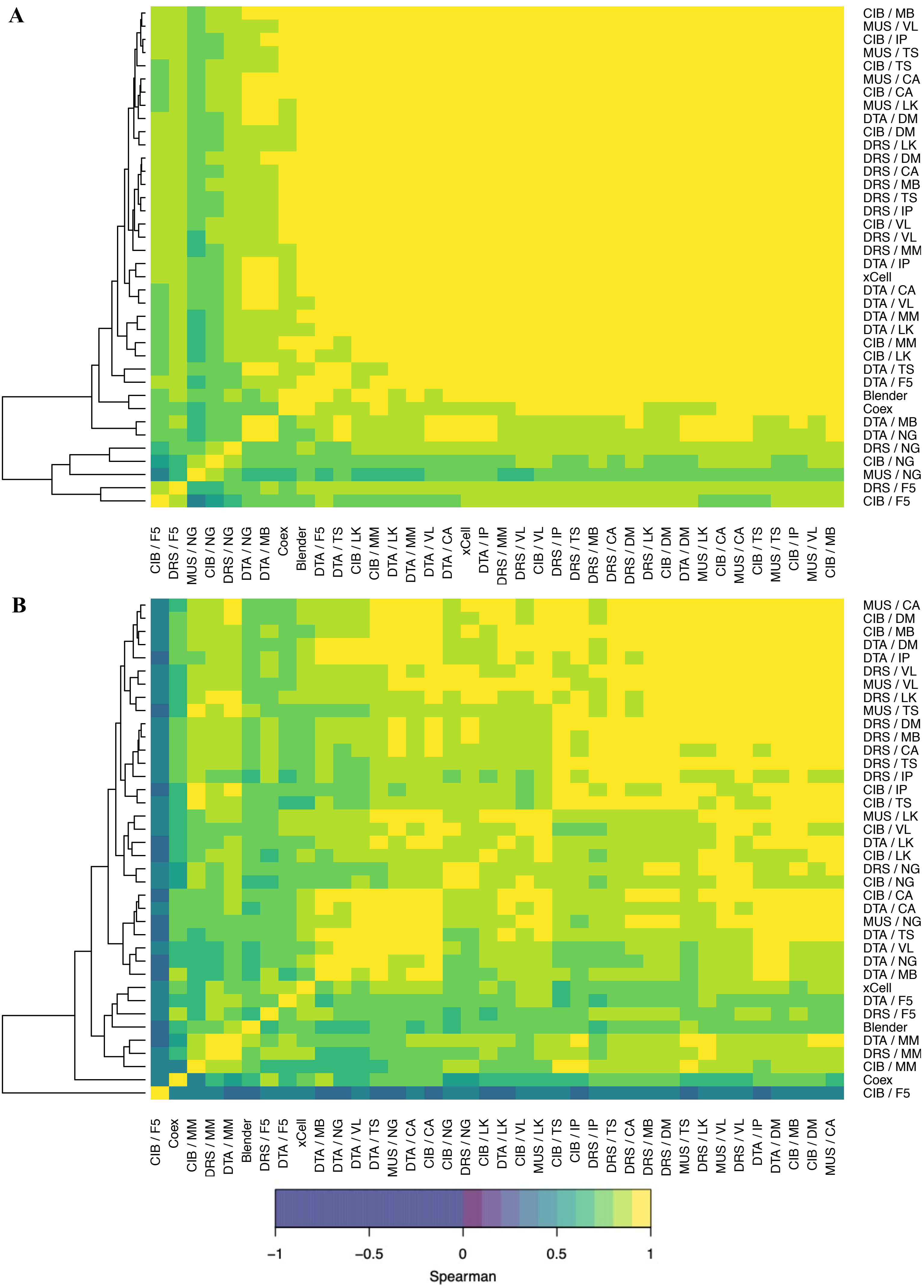

Supplementary Figure 44. Heatmap of spearman correlations in neuronal estimates across signatures and algorithms in bulk brain datasets. A. GTEx. **B.** Parikshak *et al.*. Black squares represent NA, where the cell-type estimate had a variance of 0 (typically all estimates being all 0 or all 1). *Dotted black line:* y=0.5. Row and column labels state the algorithm name followed by signature with which it was combined (if relevant). *CIB*: CIBERSORT. *DRS*: DeconRNASeq. *DTA*: dtangle. *MUS*: MuSiC. *Blender*: BrainInABlender.

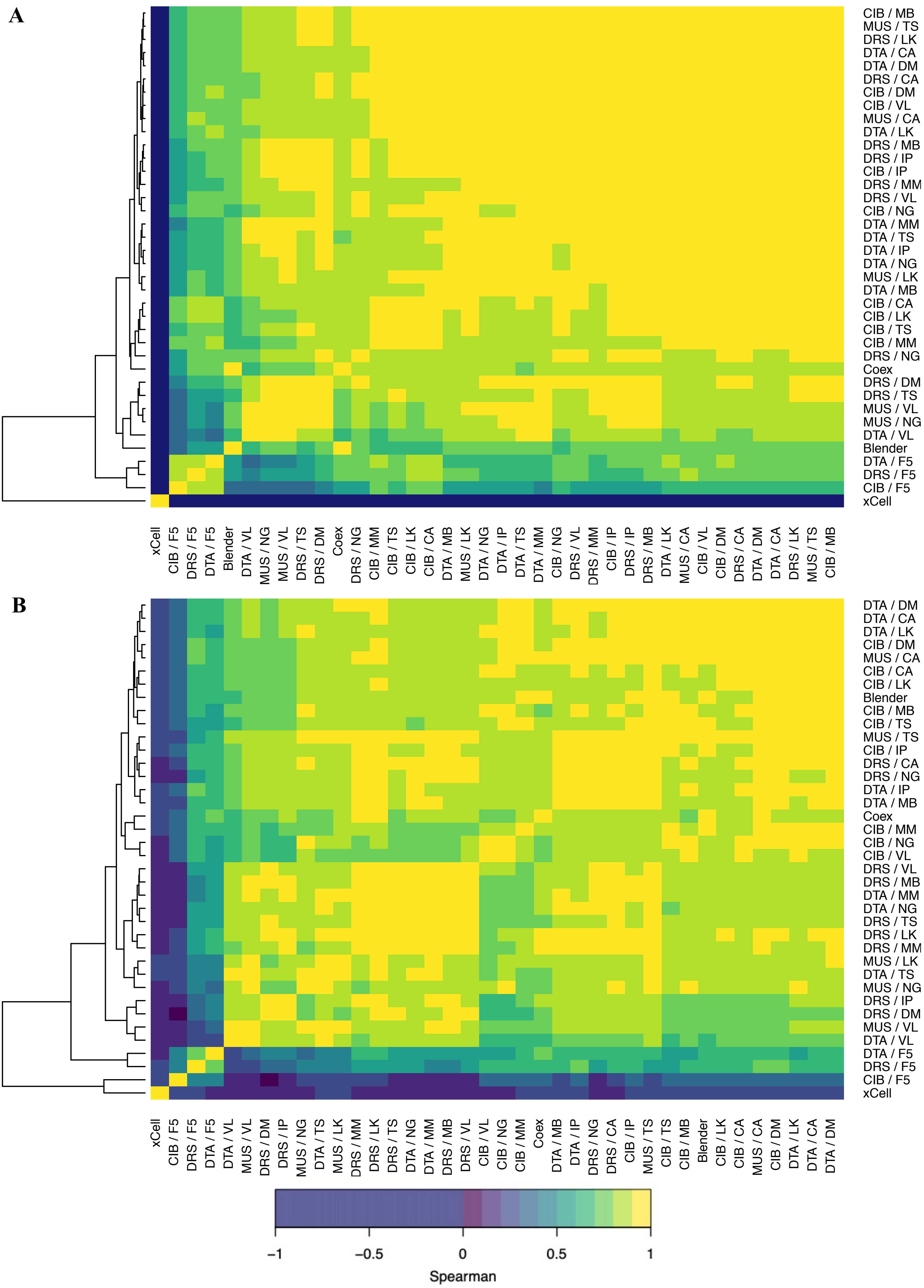

Supplementary Figure 45. Heatmap of spearman correlations in astrocyte estimates across signatures and algorithms in bulk brain datasets. A. GTEx. **B.** Parikshak *et al.*. Black squares represent NA, where the cell-type estimate had a variance of 0 (typically all estimates being all 0 or all 1). *Dotted black line:* y=0.5. Row and column labels state the algorithm name followed by signature with which it was combined (if relevant). *CIB*: CIBERSORT. *DRS*: DeconRNASeq. *DTA*: dtangle. *MUS*: MuSiC. *Blender*: BrainInABlender.

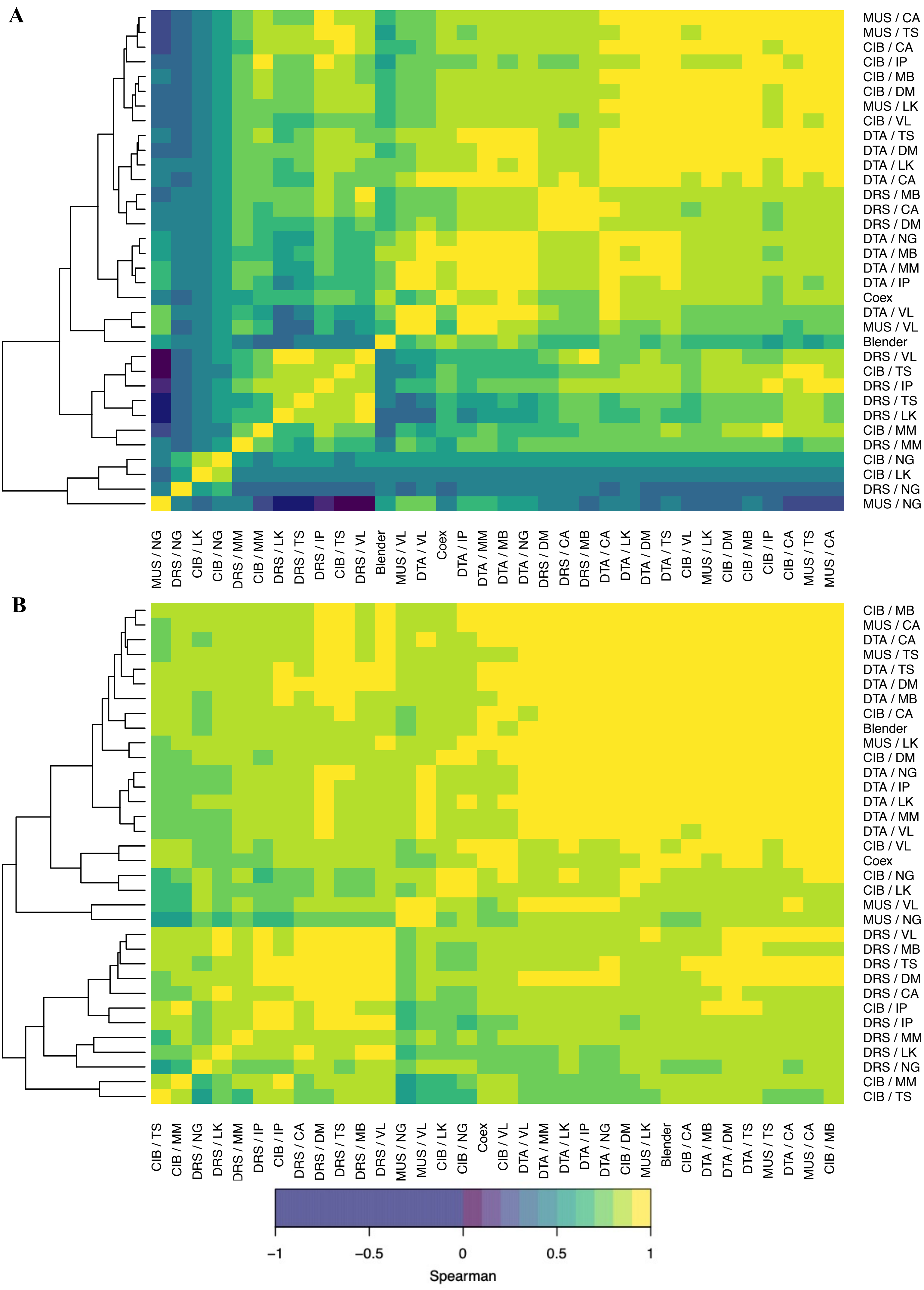

Supplementary Figure 46. Heatmap of spearman correlations in oligodendrocyte estimates across signatures and algorithms in bulk brain datasets. A. GTEx. **B.** Parikshak *et al.*. Black squares represent NA, where the cell-type estimate had a variance of 0 (typically all estimates being all 0 or all 1). *Dotted black line:* y=0.5. Row and column labels state the algorithm name followed by signature with which it was combined (if relevant). *CIB*: CIBERSORT. *DRS*: DeconRNASeq. *DTA*: dtangle. *MUS*: MuSiC. *Blender*: BrainInABlender.

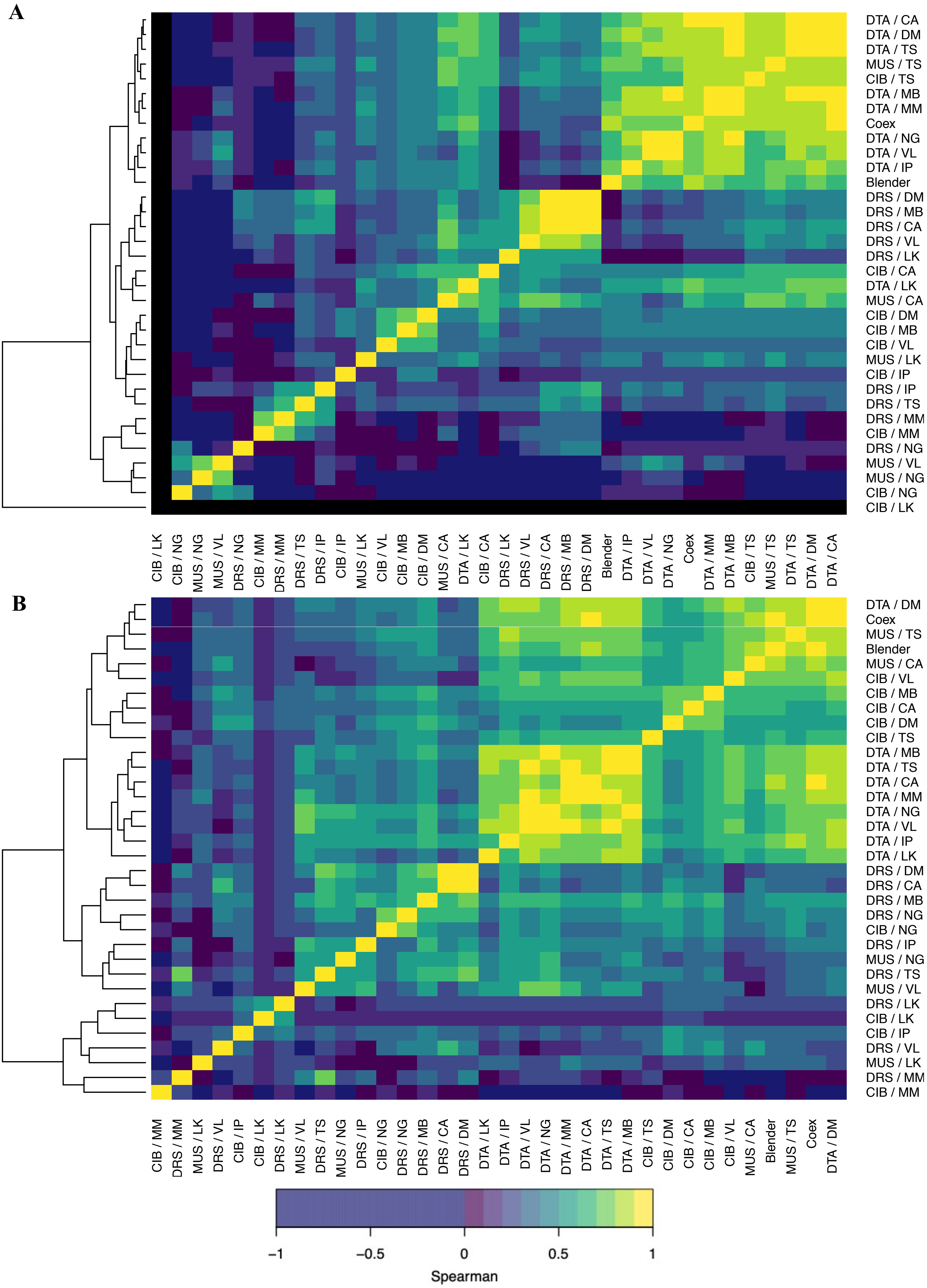

Supplementary Figure 47. Heatmap of spearman correlations in microglial estimates across signatures and algorithms in bulk brain datasets. A. GTEx. **B.** Parikshak *et al.*. Black squares represent NA, where the cell-type estimate had a variance of 0 (typically all estimates being all 0 or all 1). *Dotted black line:* y=0.5. Row and column labels state the algorithm name followed by signature with which it was combined (if relevant). *CIB*: CIBERSORT. *DRS*: DeconRNASeq. *DTA*: dtangle. *MUS*: MuSiC. *Blender*: BrainInABlender.

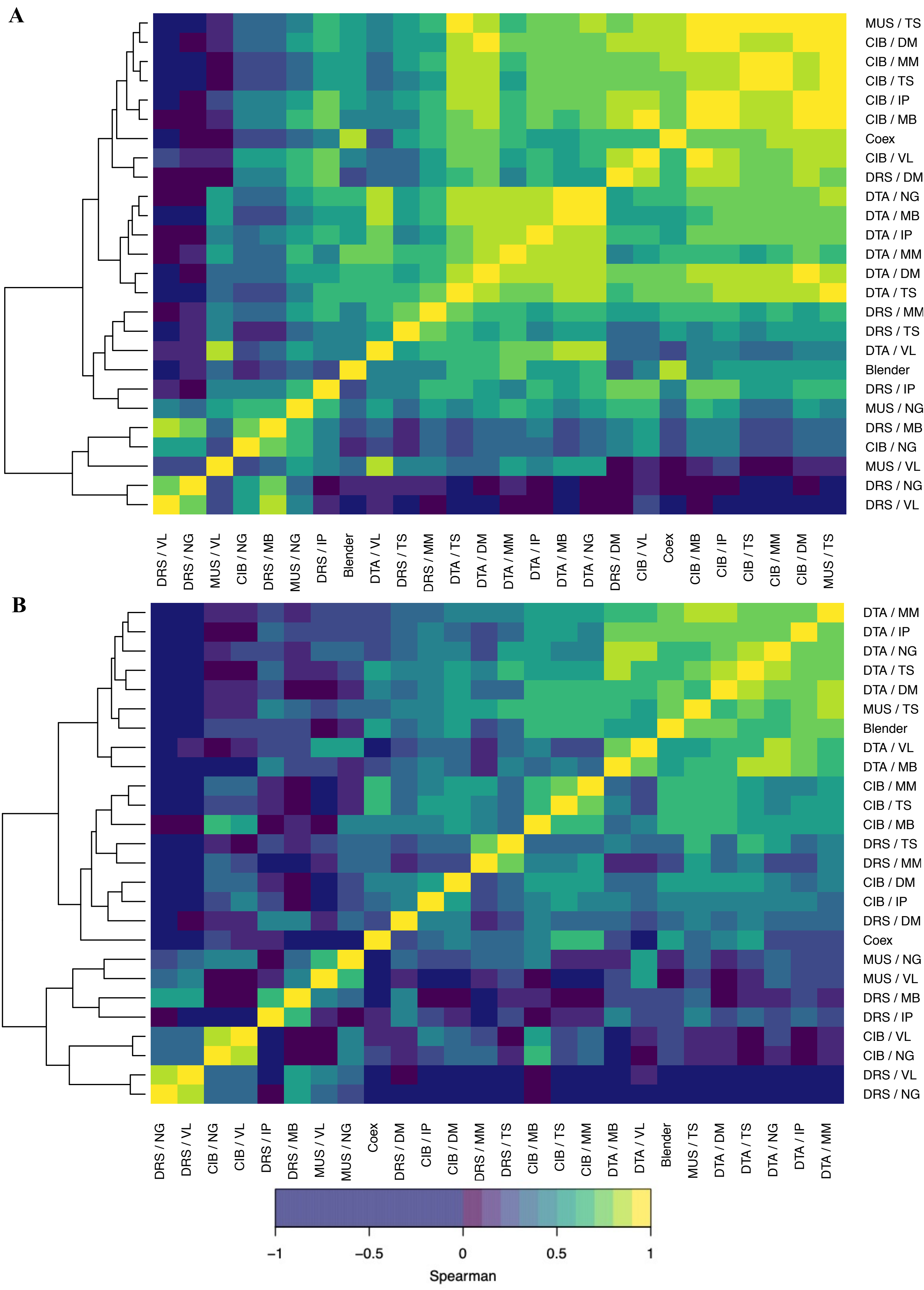

Supplementary Figure 48. Heatmap of spearman correlations in endothelial estimates across signatures and algorithms in bulk brain datasets. A. GTEx. **B.** Parikshak *et al.*. Black squares represent NA, where the cell-type estimate had a variance of 0 (typically all estimates being all 0 or all 1). *Dotted black line:* y=0.5. Row and column labels state the algorithm name followed by signature with which it was combined (if relevant). *CIB*: CIBERSORT. *DRS*: DeconRNASeq. *DTA*: dtangle. *MUS*: MuSiC. *Blender*: BrainInABlender.

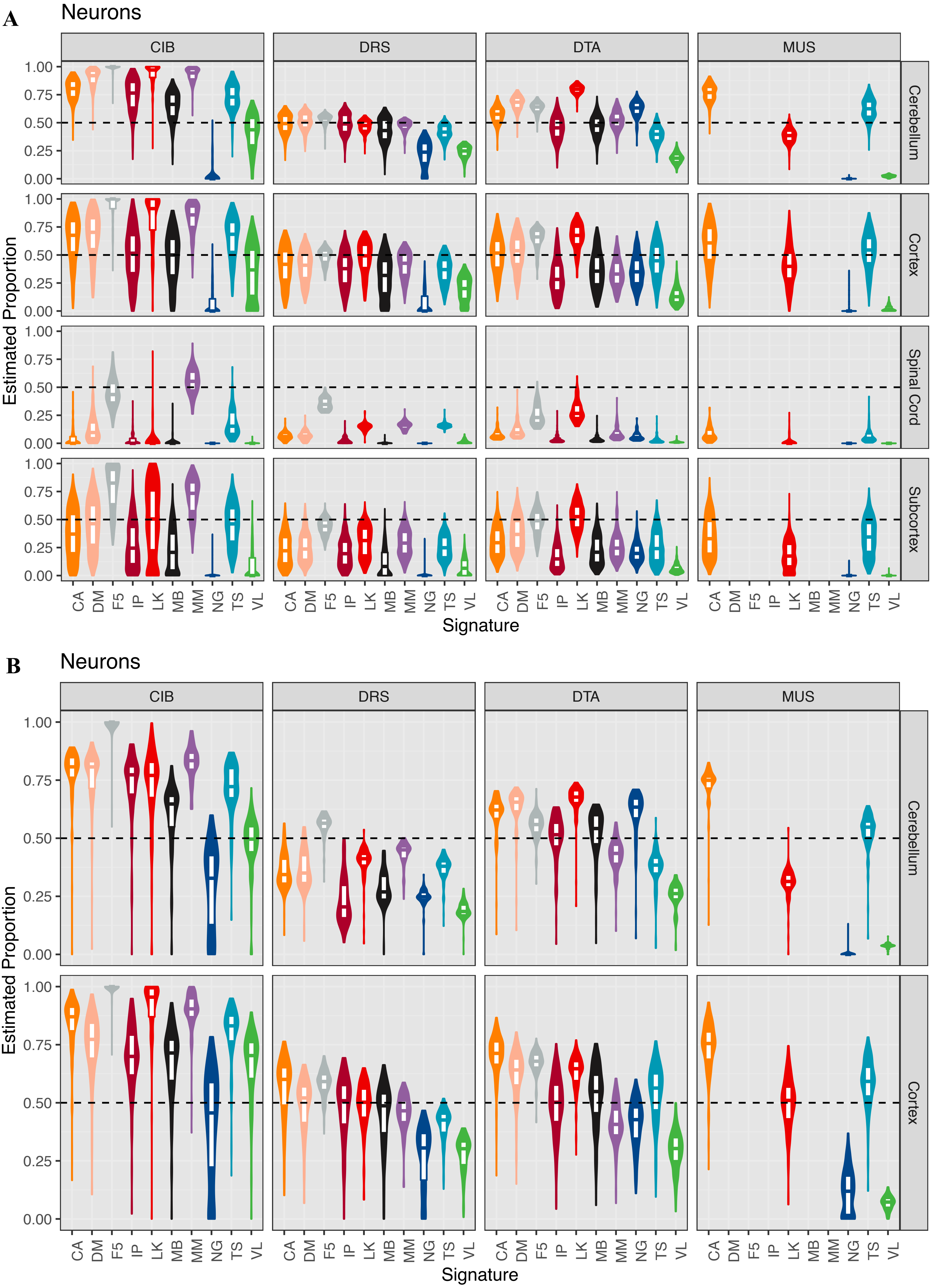

Supplementary Figure 49. Distribution of neuronal deconvolutions estimates in bulk brain datasets. Rows and columns represent different brain regions and deconvolution algorithms, respectively. **A.** GTEx cerebellum (n=309), cerebral cortex (n=408), subcortical regions (n=863) and spinal cord (n=91) samples. **B.** Parikshak *et al.* cerebellum (n=84) and cerebral cortex (n=167) samples. The width of the violin indicates point density, with the top, middle, and bottom of the white overlay box marking the 75th, 50th, and 25th percentiles, respectively. *Dotted black line:* y=0.5. *CIB*: CIBERSORT. *DRS*: DeconRNASeq. *DTA*: dtangle. *MUS*: MuSiC. See methods for further details about signatures (x-axis).

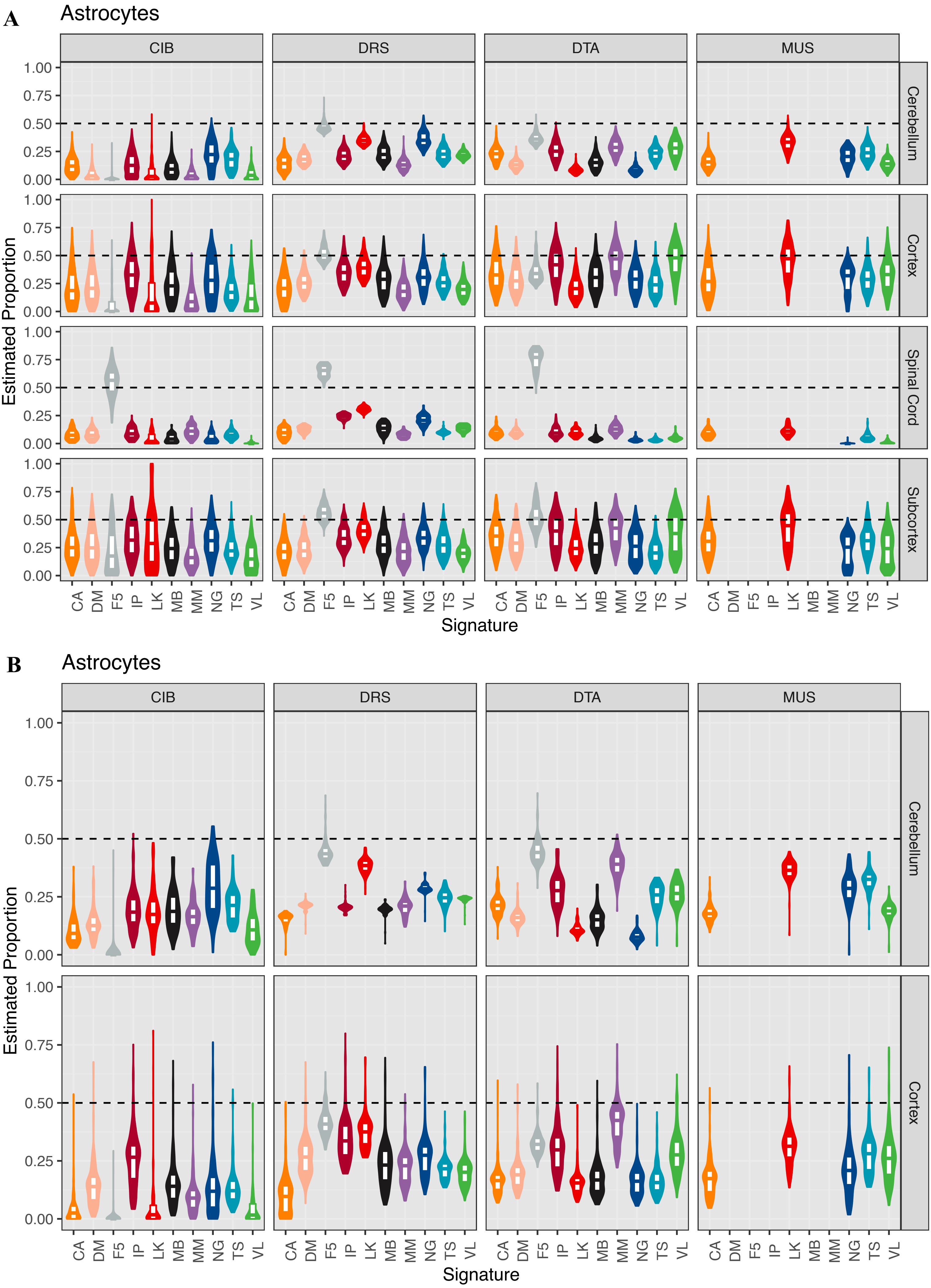

Supplementary Figure 50. Distribution of astrocyte deconvolutions estimates in bulk brain datasets. Rows and columns represent different brain regions and deconvolution algorithms, respectively. **A.** GTEx cerebellum (n=309), cerebral cortex (n=408), subcortical regions (n=863) and spinal cord (n=91) samples. **B.** Parikshak *et al.* cerebellum (n=84) and cerebral cortex (n=167) samples. The width of the violin indicates point density, with the top, middle, and bottom of the white overlay box marking the 75th, 50th, and 25th percentiles, respectively. *Dotted black line:* y=0.5. *CIB*: CIBERSORT. *DRS*: DeconRNASeq. *DTA*: dtangle. *MUS*: MuSiC. See methods for further details about signatures (x-axis).

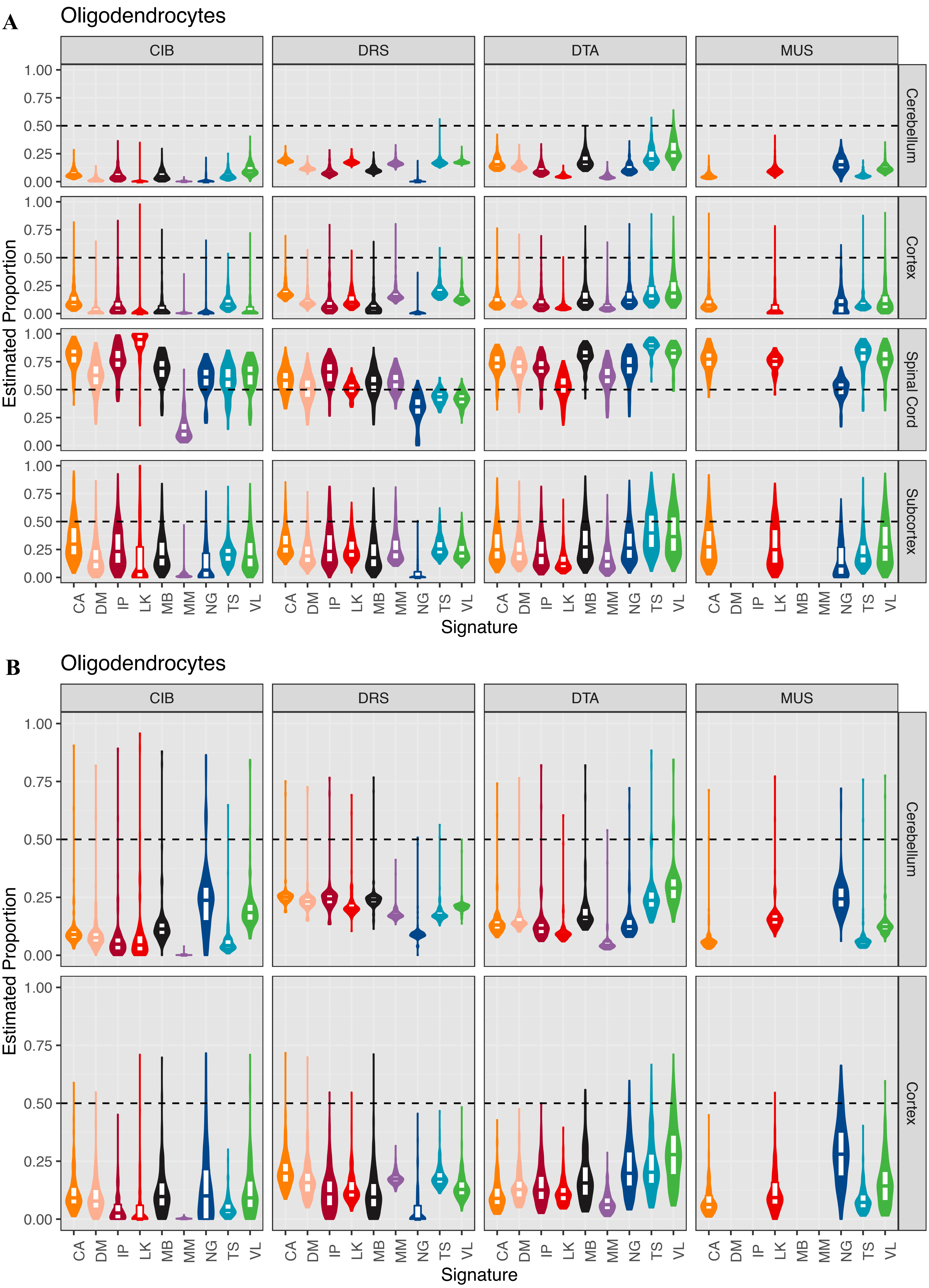

Supplementary Figure 51. Distribution of oligodendrocyte deconvolutions estimates in bulk brain datasets. Rows and columns represent different brain regions and deconvolution algorithms, respectively. **A.** GTEx cerebellum (n=309), cerebral cortex (n=408), subcortical regions (n=863) and spinal cord (n=91) samples. **B.** Parikshak *et al.* cerebellum (n=84) and cerebral cortex (n=167) samples. The width of the violin indicates point density, with the top, middle, and bottom of the white overlay box marking the 75th, 50th, and 25th percentiles, respectively. *Dotted black line:* y=0.5. *CIB*: CIBERSORT. *DRS*: DeconRNASeq. *DTA*: dtangle. *MUS*: MuSiC. See methods for further details about signatures (x-axis).

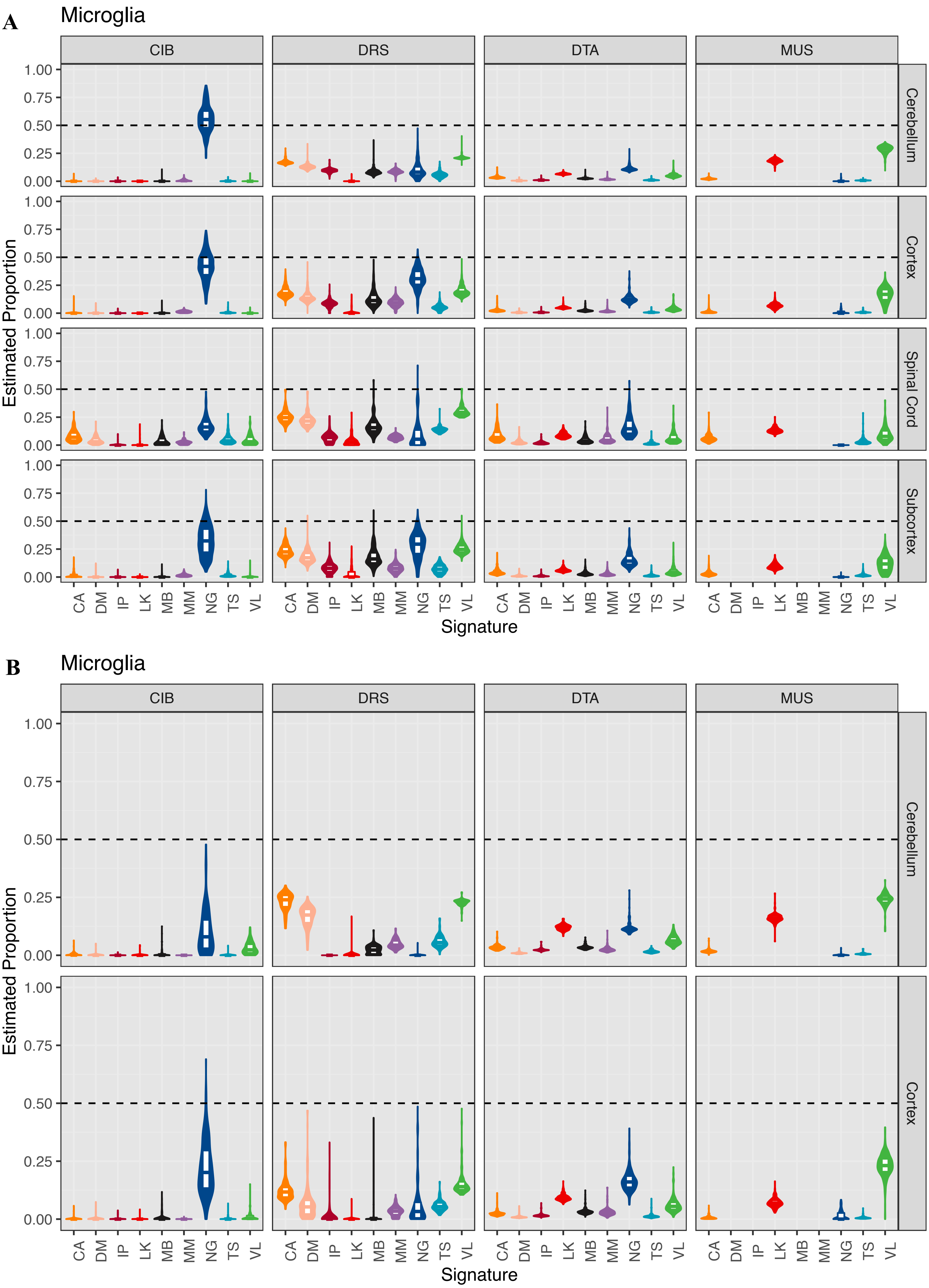

Supplementary Figure 52. Distribution of microglial deconvolutions estimates in bulk brain datasets. Rows and columns represent different brain regions and deconvolution algorithms, respectively. **A.** GTEx cerebellum (n=309), cerebral cortex (n=408), subcortical regions (n=863) and spinal cord (n=91) samples. **B.** Parikshak *et al.* cerebellum (n=84) and cerebral cortex (n=167) samples. The width of the violin indicates point density, with the top, middle, and bottom of the white overlay box marking the 75th, 50th, and 25th percentiles, respectively. *Dotted black line:* y=0.5. *CIB*: CIBERSORT. *DRS*: DeconRNASeq. *DTA*: dtangle. *MUS*: MuSiC. See methods for further details about signatures (x-axis).

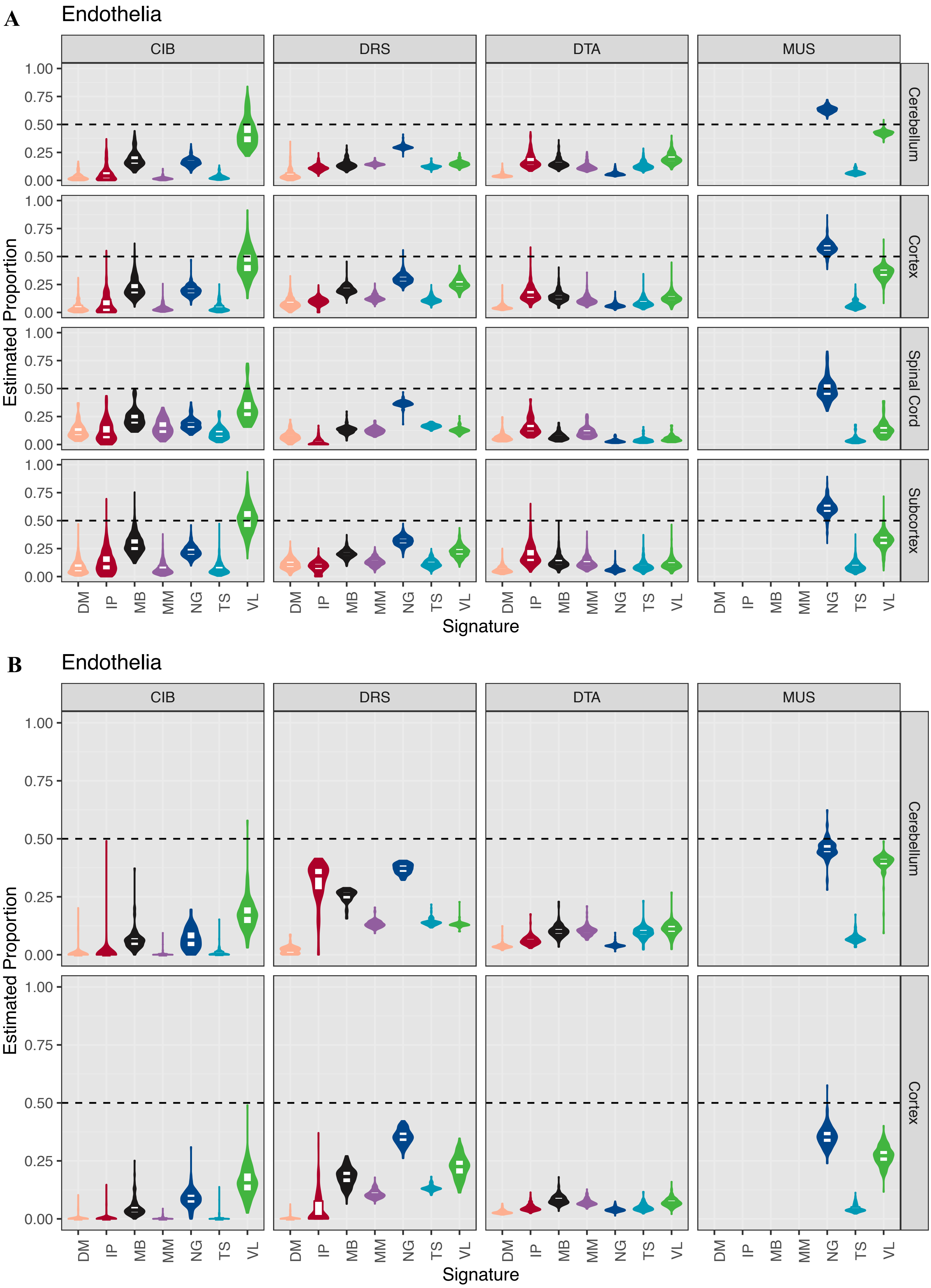

Supplementary Figure 53. Distribution of endothelial deconvolutions estimates in bulk brain datasets. Rows and columns represent different brain regions and deconvolution algorithms, respectively. **A.** GTEx cerebellum (n=309), cerebral cortex (n=408), subcortical regions (n=863) and spinal cord (n=91) samples. **B.** Parikshak *et al.* cerebellum (n=84) and cerebral cortex (n=167) samples. The width of the violin indicates point density, with the top, middle, and bottom of the white overlay box marking the 75th, 50th, and 25th percentiles, respectively. *Dotted black line:* y=0.5. *CIB*: CIBERSORT. *DRS*: DeconRNASeq. *DTA*: dtangle. *MUS*: MuSiC. See methods for further details about signatures (x-axis).
