# Supplementary material for: Comprehensive evaluation of deconvolution methods for human brain gene expression
Source: Nat Commun. 2022 Mar 15;13:1358. doi: 10.1038/s41467-022-28655-4 (PMC8924248; doi:10.1038/s41467-022-28655-4)
Supplement: Supplementary file 3 — Description of Additional Supplementary Files [file 41467_2022_28655_MOESM3_ESM.pdf]

Description of Additional Supplementary Files

File Name: Supplementary Data 1
Description: **Differential expression analysis comparing nuclear and whole-cell brain tissue preparations.** Contains differential expression results from nuclear vs. whole-cell brain tissue, including those genes deemed to be compartment-specific.

File Name: Supplementary Data 2
Description: **Composition estimates in pure immuno-panned brain cell-types using either all genes or removing compartment-specific genes.** Contains deconvolution estimates for data from Zhang et al., when using either all genes or removing compartment-specific genes from deconvolution.

File Name: Supplementary Data 3
Description: **Composition estimates in GTEx and Parikshak *et al.* bulk brain transcriptomes, across signatures and methods.** Each cell shows the estimated proportion or cell-type enrichment in bulk brain samples, as a function of signature and algorithm.

File Name: Supplementary Data 4
Description: **Differentially expression analysis results for ASD samples *vs.* controls for composition-dependent (CD) and composition-independent (CI) analyses.** Contains differential expression results and GO term enrichments from differentially-expressed genes

File Name: Supplementary Data 5
Description: **Cell-type specific gene expression signature data.** Contains expression values for each gene across signatures and cell-types.

File Name: Supplementary Data 6
Description: **List of datasets accessed and the samples included in the present study from each dataset.** Contains a list of brain datasets accessed and how each sample was used in our analyses

File Name: Supplementary Data 7
Description: **Summary of RNA-seq data generated in the current study. Tab1: Summary of RNA-seq data generated from mixtures of RNA from cultured cells.** Contains key quality control metrics for RNA-seq data generated in the current study
