# Supplementary material for: Comprehensive evaluation of deconvolution methods for human brain gene expression
Source: Nat Commun. 2022 Mar 15;13:1358. doi: 10.1038/s41467-022-28655-4 (PMC8924248; doi:10.1038/s41467-022-28655-4)
Supplement: Supplementary file 11 — Reporting Summary [file 41467_2022_28655_MOESM11_ESM.pdf]

nature portfolio

Corresponding author(s): Irina Voineagu

Last updated by author(s): Nov 18, 2021

Reporting Summary

Nature Portfolio wishes to improve the reproducibility of the work that we publish. This form provides structure for consistency and transparency in reporting. For further information on Nature Portfolio policies, see our Editorial Policies and the Editorial Policy Checklist.

Statistics

For all statistical analyses, confirm that the following items are present in the figure legend, table legend, main text, or Methods section.

n/a	Confirmed	
☐	☒	The exact sample size (*n*) for each experimental group/condition, given as a discrete number and unit of measurement
☐	☒	A statement on whether measurements were taken from distinct samples or whether the same sample was measured repeatedly
☐	☒	The statistical test(s) used AND whether they are one- or two-sided<br>*Only common tests should be described solely by name; describe more complex techniques in the Methods section.*
☐	☒	A description of all covariates tested
☐	☒	A description of any assumptions or corrections, such as tests of normality and adjustment for multiple comparisons
☐	☒	A full description of the statistical parameters including central tendency (e.g. means) or other basic estimates (e.g. regression coefficient) AND variation (e.g. standard deviation) or associated estimates of uncertainty (e.g. confidence intervals)
☐	☒	For null hypothesis testing, the test statistic (e.g. *F*, *t*, *r*) with confidence intervals, effect sizes, degrees of freedom and *P* value noted<br>*Give P values as exact values whenever suitable.*
☒	☐	For Bayesian analysis, information on the choice of priors and Markov chain Monte Carlo settings
☒	☐	For hierarchical and complex designs, identification of the appropriate level for tests and full reporting of outcomes
☐	☒	Estimates of effect sizes (e.g. Cohen's *d*, Pearson's *r*), indicating how they were calculated

Our web collection on statistics for biologists contains articles on many of the points above.

Software and code

Policy information about availability of computer code

Data collection	No software was used for data collection
Data analysis	All custom R code is available at https://github.com/Voineagulab/BrainCellularComposition.<br>The following software was used in data analysis: STAR-2.5.2b; Cell Ranger v2.1.0; the CIBERSORTx webtool at https://cibersortx.stanford.edu/, and R v3.6. Within R v3.6, the following external packages were used: Seurat v3; CIBERSORT v1.04; DeconRNASeq v1.26; MuSiC v0.1.1; dtangle v0.3.1; BrainInABlender v0.9; xCell v1.1.0; Linseed v0.99; DESeq2 v1.22.2; gProfiler2 v0.2; singleR v1.0.6; WGCNA v1.70-3.

For manuscripts utilizing custom algorithms or software that are central to the research but not yet described in published literature, software must be made available to editors and reviewers. We strongly encourage code deposition in a community repository (e.g. GitHub). See the Nature Portfolio guidelines for submitting code & software for further information.

Data

Policy information about availability of data

All manuscripts must include a data availability statement. This statement should provide the following information, where applicable:

- Accession codes, unique identifiers, or web links for publicly available datasets
- A description of any restrictions on data availability
- For clinical datasets or third party data, please ensure that the statement adheres to our policy

The sequencing data generated in the present study is available on GEO/SRA accession number GSE175772. RNA-seq data for bulk brain tissue was accessed from the following 2 resources: Parikshak et al (2016) (https://github.com/dhglab/Genome-wide-changes-in-lncRNA-alternative-splicing-and-cortical-patterning-in-

autism/releases); and GTEx v7 release (https://gtexportal.org/home/datasets). Brain cell-type specific expression was accessed from the following 9 resources: FANTOM5 (http://fantom.gsc.riken.jp/5/data/); Zhang et al. (2016) (GSE73721); Zhang et al. (2014) (https://web.stanford.edu/group/barres_lab/brain_rnaseq.html); Darmanis et al. (2015) (https://github.com/VCCRI/CIDR-examples/tree/master/Brain); Lake et al. (2018) (GSE97942); Velmeshev et al. (2019) (https://autism.cells.ucsc.edu/); The Human Cell Atlas (http://portal.brain-map.org/); Nagy et al. (2020) (GSE144136); and Tasic et al. (2018) (GSE115746). Cell-type-specific expression for non-brain tissues were accessed from the following four sources: Enge et al. (2017) (GSE81547); Blodgett et al. (2015) (GSE67543); Furuyama et al. (2019) (GSE117454); ENCODE (https://www.encodeproject.org/publication-data/ENCSR590RJC/); and Wang et al. (2020) (GSE109816)

Field-specific reporting

Please select the one below that is the best fit for your research. If you are not sure, read the appropriate sections before making your selection.

☒ Life sciences ☐ Behavioural & social sciences ☐ Ecological, evolutionary & environmental sciences

For a reference copy of the document with all sections, see nature.com/documents/nr-reporting-summary-flat.pdf

Life sciences study design

All studies must disclose on these points even when the disclosure is negative.

Sample size	No sample size calculation was performed to predetermine sample size. Instead, sample sizes for differential expression simulations (n=100, with n=50 per group for differential expression simulations) were chosen to be similar to the number of samples in a real dataset we analysed, of Autism vs. Control cortical RNA-seq from publicly-available data in Parikshak et al. (2016) (n=43 and 63, autism and control samples, respectively). Thus, the simulated sample size will have a similar power to detect changes introduced by composition, allowing us to compare our effect sizes in simulation versus those observed in real data.
Data exclusions	Four samples were excluded from the analyses of 251 RNA-seq samples from Parikshak et al., 2016 (PMID: 22730494). We note that these were the same samples as excluded in the original paper's analyses: those whose mean sample-sample correlation was > 2 standard deviations from the other samples.
Replication	Our benchmarking was replicated across a range of diverse and independent datasets: three in silico simulated datasets generated using independent single-cell / single-nucleus datasets; in vitro RNA mixtures; and two independent bulk brain RNA-seq consortium datasets. We confirmed that these replicated all major results, including the effect of signature on accuracy, and partial deconvolution being the most suitable. DE Simulations were not replicated. Analyses of ASD vs Control RNA-seq were not replicated as no similarly sized datasets were available.
Randomization	Randomisation is not relevant to previously published data used in this study for benchmarking of deconvolution methods. RNA-seq data generated in the present study from in-vitro RNA mixtures was generated in two batches: the first batch including the pure neuronal sample and the three mixture samples, and the second batch including the pure astrocyte samples. Brain tissue samples were processed individually for snRNA-seq, and as a single batch for bulk RNA-seq. Note that these samples were used for estimation of cell type proportions rather than group comparisons, thus randomisation is not relevant to the benchmarking analyses.
Blinding	Blinding is not relevant to the benchmarking analyses presented in this study as all analyses were quantitative and thus blinding would not influence levels of bias.

Reporting for specific materials, systems and methods

We require information from authors about some types of materials, experimental systems and methods used in many studies. Here, indicate whether each material, system or method listed is relevant to your study. If you are not sure if a list item applies to your research, read the appropriate section before selecting a response.

Materials & experimental systems

n/a	Involved in the study	
☒	☐	Antibodies
☐	☒	Eukaryotic cell lines
☒	☐	Palaeontology and archaeology
☒	☐	Animals and other organisms
☐	☒	Human research participants
☒	☐	Clinical data
☒	☐	Dual use research of concern

Methods

n/a	Involved in the study	
☒	☐	ChIP-seq
☒	☐	Flow cytometry
☒	☐	MRI-based neuroimaging

Eukaryotic cell lines

Policy information about cell lines

Cell line source(s)	NHA normal human astrocytes Lonza (#CC-2565)

Authentication	no authentication
Mycoplasma contamination	not tested for mycoplasma contamination
Commonly misidentified lines (See ICLAC register)	No commonly misidentified cell-lines were used in this study

Human research participants

Policy information about studies involving human research participants

Population characteristics	Brain tissue samples from 5 individuals were obtained from the NICHD Brain and Tissue Bank, and included frontal cortex samples (BA9/10) from 2 control, 2 ASD, and 1 Fragile-X premutation carrier individuals. Ages range from 7-85. 4 of the individuals are male, and 1 is female.
Recruitment	De-identified post-mortem samples were obtained from the NIH NeuroBioBank.
Ethics oversight	Post-mortem human brain tissue transcriptome analyses were undertaken under a protocol approved by the University of Western Australia Human Research Ethics Committee (RA/4/20/6394).

Note that full information on the approval of the study protocol must also be provided in the manuscript.
